# Haplotype-Based Single-Step GWAS for Yearling Temperament in American Angus Cattle

**DOI:** 10.3390/genes13010017

**Published:** 2021-12-22

**Authors:** Andre C. Araujo, Paulo L. S. Carneiro, Amanda B. Alvarenga, Hinayah R. Oliveira, Stephen P. Miller, Kelli Retallick, Luiz F. Brito

**Affiliations:** 1 Graduate Program in Animal Sciences, State University of Southwestern Bahia, Itapetinga 45700-000, Brazil; araujoa@purdue.edu; 2 Department of Animal Science, Purdue University, West Lafayette, IN 47907, USA; alvarena@purdue.edu (A.B.A.); hinayah@gmail.com (H.R.O.); 3 Department of Biology, State University of Southwest Bahia, Jequié 45205-490, Brazil; plscarneiro@uesb.edu.br; 4 Centre for Genetic Improvement of Livestock, Department of Animal Biosciences, University of Guelph, Guelph, ON N1G2W1, Canada; 5 American Angus Association, Angus Genetics Inc., 3201 Frederick Ave, St. Joseph, MO 64506, USA; SMiller@angus.org (S.P.M.); kretallick@angus.org (K.R.)

**Keywords:** candidate genes, functional analysis, haplotype block, linkage disequilibrium, livestock behavior, pseudo-SNPs, social interaction

## Abstract

Behavior is a complex trait and, therefore, understanding its genetic architecture is paramount for the development of effective breeding strategies. The objective of this study was to perform traditional and weighted single-step genome-wide association studies (ssGWAS and WssGWAS, respectively) for yearling temperament (YT) in North American Angus cattle using haplotypes. Approximately 266 K YT records and 70 K animals genotyped using a 50 K single nucleotide polymorphisms (SNP) panel were used. Linkage disequilibrium thresholds (LD) of 0.15, 0.50, and 0.80 were used to create the haploblocks, and the inclusion of non-LD-clustered SNPs (NCSNP) with the haplotypes in the genomic models was also evaluated. WssGWAS did not perform better than ssGWAS. Cattle YT was found to be a highly polygenic trait, with genes and quantitative trait loci (QTL) broadly distributed across the whole genome. Association studies using LD-based haplotypes should include NCSNPs and different LD thresholds to increase the likelihood of finding the relevant genomic regions affecting the trait of interest. The main candidate genes identified, i.e., *ATXN10*, *ADAM10*, *VAX2*, *ATP6V1B1*, *CRISPLD1*, *CAPRIN1*, *FA2H*, *SPEF2*, *PLXNA1*, and *CACNA2D3*, are involved in important biological processes and metabolic pathways related to behavioral traits, social interactions, and aggressiveness in cattle. Future studies should further investigate the role of these candidate genes.

## 1. Introduction

Behavior is a complex trait influenced by multiple factors (e.g., age, health status, life experiences, genetics) and the interaction among group-housed individuals and the environment [1]. Emotional or behavioral responses are actions resultant of feedback from the central nervous system after decodifying an external stimulus, which has been studied for a long time in humans [2]. Prior to domestication, animals presented different behavior characteristics compared to domesticated populations, indicating that behavioral traits can be genetically modified through selective breeding [1]. Livestock behavior is important due to its impact in several other relevant traits for the industry, including production, reproduction, and both animal and handler’s welfare and health [3,4,5]. Docile temperament is a desired behavior in cattle because it facilitates the handling process and it has been proven to be favorably associated with meat quality, productive efficiency, and welfare traits [6]. An indicator of temperament used for selection in North American Angus cattle is yearling temperament (YT). YT is subjectively scored by farmers/handlers when a one-year-old calf is being processed through the chute and should be an observation of how animals enter, exit, and react while being handled [7]. A previous study has shown that YT is heritable (heritability ~0.38), suggesting genetic progress can be achieved through direct selection [5]. Additionally, a multi-species systematic review reported 797 genomic regions and 383 candidate genes associated with behavioral traits in cattle [8]. Only six genes (*GRM5*, *MAML3*, *C8B*, *RUSC2*, *POMC*, *MIPOL1*, and *SLC18A2*) were in overlap among trait definitions and populations [8], suggesting a natural particularity of each population and measurement definition.

Using alternative approaches, such as haplotype-based methods, for detecting genomic regions influencing YT is of great interest to the beef cattle industry. Haplotypes are usually defined as a set of adjacent loci expected to be inherited together with a small probability of recombination [9]. Haplotype blocks (i.e., haploblocks) are sets of adjacent single nucleotide polymorphisms (SNPs) markers expected to be in higher linkage disequilibrium (LD) with the quantitative trait loci (QTL) than single SNPs [10,11]. Furthermore, haplotypes can also capture small epistatic effects within haploblocks [12,13,14], justifying the advantages of using haplotypes for both genomic prediction of breeding values [12,13,14] and genome-wide association studies (GWAS) [14,15,16]. However, due to the more complex implementation of haplotype-based methods, they have been underused compared to SNP-based methods [17,18]. For GWAS purposes, the combination of SNP- and haplotype-based methods are recommended because it might increase the possibility of capturing different types of QTL [15,16], i.e., different sizes (spanning small or large genomic regions), allelic frequency, and LD levels with SNPs due to differential recombination rates. 

Both phenotypes and genotypes are required when performing GWAS; however, not all phenotyped individuals in a population are genotyped and vice-versa [19,20]. In this context, Single-step Genomic Best Linear Unbiased Prediction (ssGBLUP) is a method that simultaneously combines information from phenotypes, pedigree, and genotypes when calculating genomic estimated breeding values (GEBV) for both genotyped and non-genotyped individuals [21,22]. Therefore, single-step GWAS (ssGWAS), which uses GEBV from ssGBLUP to derive SNP effects, is an efficient method to perform GWAS because phenotypes from genotyped and ungenotyped individuals are used to more accurately derive SNP effects [20]. However, the infinitesimal model (many loci explaining similar and small proportions of the total additive genetic variance) is an assumption of both ssGBLUP and ssGWAS [19]. As some loci (major genes) can explain major proportions of the additive variance of the traits of interest, weighted ssGBLUP (WssGBLUP) and its GWAS version (WssGWAS) were proposed to prioritize markers potentially explaining larger proportions of the total additive genetic variance [19,23].

Despite its relevance in animal breeding, to the best of our knowledge, no haplotype-based GWAS has been used to investigate the genetic background of temperament in cattle. Additionally, there is a lack of studies implementing haplotypes under ssGWAS and WssGWAS approaches. Therefore, the objective of this study was to perform a haplotype-based ssGWAS for YT in American Angus cattle. Different haplotype-based GWAS approaches were implemented and tested to uncover the potential genomic regions associated with YT, including: (1) different LD thresholds to create the LD-based haplotypes, (2) including or not including the non-LD-clustered SNPs in the association analyses, and (3) ssGWAS and WssGWAS. Understanding the genetic background of behavioral traits is of great interest for the beef cattle industry because it could enable the optimization of genetic selection for more docile animals in which the genetic progress would be permanent and cumulative over generations. Genetic or genomic selection for any trait impacts several biological mechanisms involved in the phenotypic expression of the trait under selection as well as genetically correlated traits. Therefore, it is paramount to understand these underlying biological mechanisms. 

## 2. Materials and Methods

### 2.1. Phenotypic and Pedigree Data

The American Angus Association (through Angus Genetics Inc.; St. Joseph, MO, USA) provided the phenotypic, genotypic, and pedigree datasets. In total, 266,029 animals recorded for YT, born between 2001 and 2018, were available for the analyses. The phenotypic dataset has previously been processed for quality measurements (please see Alvarenga et al. [5] for a complete description of the data). Briefly, yearling temperament is a categorical trait recorded using six scores (from 1 to 6), in which 1 represents docile and 6 represents very aggressive animals. For more details about the codification and criteria to classify the animals, please see [5,7]. From the total number of records, 71.9% were classified as docile (score 1), 22.2% as restless (score 2), 5.1% as nervous (score 3), and 0.8% as aggressive (scores 4 to 6). The scores 4, 5, and 6, which represents flighty, aggressive, and very aggressive, respectively, were grouped together as a single category (aggressive) due to their low incidence [5]. The number of animals per management class in the phenotypic data were: 147,671 bulls, 3332 steers, and 115,026 females. The pedigree data initially had 4,410,551 animals born from 1836 to 2018, and 578,819 individuals remained to construct the pedigree-based additive genetic relationship matrix (**A**), tracing back ancestors up to four generations. 

### 2.2. Genotypic Data

The genotypic dataset included 69,559 animals genotyped using a 50 K SNP panel (54,609 SNP markers) of an imputed SNP set similar to the Illumina BovineSNP50V2 and Illumina BovineSNP50V3 (Illumina, Inc., San Diego, CA, USA), designed for commercial purposes. Markers with minor allele frequency (MAF) < 0.01, call rate < 0.90, difference between observed and expected heterozygosity > 0.15 (i.e., extreme departure from Hardy-Weinberg equilibrium), and not present in the pseudo-autosomal region (PAR) in the X chromosome were removed from the genotypic data as part of the quality control (QC). PAR was considered the region above BTAX:133,300,518 bp [24]. Additionally, animals with call rates lower than 0.90 were also removed. The QC in the genotypic data was done using the PREGSf90 software from the BLUPf90 family software [25]. After QC, 42,633 markers and 69,437 animals were kept for further analyses. 

### 2.3. Haplotype Block Construction

The haplotype block (haploblock) construction process started with phasing the SNP genotypes in the FImpute software v.3.0 [26]. After phasing, haploblocks were constructed with a variable size approach using the LD values measured by the $r^{2}$ metric [27]. The Big-LD method [28] was used to construct the blocks because it is more computationally efficient and accurate in estimating the recombination hotspots than other commonly used algorithms [28]. The “gpart” package [29] implemented in the R software [30] was used to implement the Big-LD method for constructing the haploblocks. As the QTL can have different genetic structures, the LD thresholds of 0.15, 0.50, and 0.80 were used to create the haploblocks to account for different recombination levels within regions, assumed to be high, medium, and low, respectively. In other words, these LD thresholds were used to capture different block structures: bigger blocks with more SNPs in low LD (0.15), intermediary blocks with moderate LD (0.50), and smaller blocks with lower number of SNPs in high LD (0.80). Furthermore, the haploblocks used in this research followed the definition proposed by Gabriel et al. [9], being sizable regions delimited to at least two loci (SNPs).

### 2.4. Single-Step GWAS with Haplotypes

The unique haplotype alleles within the haploblocks were coded as pseudo-SNPs, which were submitted to the same QC as the SNPs (described in Section 2.2) for performing the ssGWAS with haplotypes. The pseudo-SNPs were coded as 0, 1, or 2 for the absence of both copies, presence of one copy, or presence of two copies of the reference haplotype allele [31]. Thereafter, the THRGIBBS1f90 software [25] was used to predict the GEBV for all individuals considering YT as a categorical trait (threshold model) and the pseudo-SNPs to construct the genomic relationship matrix. 

The contemporary groups (CG) and the animal model used to predict the YT GEBV were previously defined by Alvarenga et al. [5], i.e.:

(1) y=Xb+Ww+Zu+e,

where y is the vector of phenotypic records for YT, b is the vector of systematic effects (age of dam, conception type, and calf age deviation from 365 days as linear covariate), w is the random vector of CG effects with $w\;\sim N\left(0,I\sigma_{w}^{2}\right)$, u is the random vector of additive genetic effects with $u\;\sim N\left(0,H\sigma_{g}^{2}\right)$, and e is the random residual term with $e\;\sim N\left(0,I\sigma_{e}^{2}\right)$. CG was formed by concatenating birth month and year, birth herd, birth sex, weaning date, weaning herd, weaning sex, creep feeding offered or not, date of temperament measurement, YT measurement herd, sex at the YT measurement, temperament group age deviation, and presence of ultrasound records (measure of additional human-animal interaction). X, W, and Z are the incidence matrices for the systematic, CG, and additive genetic effects, respectively. A diagonal matrix with large values, $\Sigma_{b}$, was used to represent a vague prior for the systematic effects. The  H matrix is the matrix that combines the pedigree and genomic relationship matrices [21], and its inverse ($H^{-1}$) was directly computed as [22]:

(2) $$H^{-1}{=A}^{-1}+\left[\begin{array}{cc} 0 & 0\\ 0 & {\tau (\alpha G+\beta A}_{22}{)}^{-1}-{\omega A}_{22}^{-1} \end{array}\right],$$

where $A^{-1}$ is the inverse of the A matrix, G is the genomic relationship matrix computed using the pseudo-SNPs, and $A_{22}^{-1}$ is the inverse of the pedigree-based relationship matrix between genotyped individuals. The default value (1.0) was used for the scaling parameters (τ and ω), while 0.90 and 0.10 were used for the weighting parameters α and β, respectively, in the PREGSf90 package [25]. G was computed as in the first method proposed by VanRaden [32], which had the following structure:

(3) $$G=\frac{\left(M-2p\right)D{\left(M-2p\right)}^{\prime }}{2\sum \;p_{i}\left(1-p_{i}\right)}$$

where M is the *n* × *m* (number of individuals by number of markers, respectively) matrix of genotype calls (0, 1, or 2), p is a vector with the allelic frequencies $p_{i}$ for the markers, and D is a *m* × *m* diagonal matrix that corresponds to an identity matrix (I) when G is applied with the same weight (i.e., 1) for the markers (i.e., ssGWAS).

The variance components (i.e., $\sigma_{w}^{2}$, $\sigma_{g}^{2},$ and $\sigma_{e}^{2}$) for YT in the American Angus population using the above model were previously estimated by Alvarenga et al. [5] and were fixed to predict the GEBVs in this study. The chain parameters used to make the predictions were 10,000 iterations, in which 1000 were discarded as burn-in, and 10 was used as thin. After the GEBV prediction, the pseudo-SNP effects were back-solved using the POSTGSf90 software [25]. The formula to back-solve the pseudo-SNP effects is [33]:

(4) $$g\;=\;D{\left(M-2p\right)}^{\prime }G^{-1}\hat{u}$$

where g is the vector of marker effects, $G^{-1}$ is the inverted G matrix, $\hat{u}$ is the vector of predicted GEBV, and all other matrices and vectors were described above. In addition to the marker effects, the POSTGSf90 software [25] was also used to calculate the percentage of the total additive genetic variance explained by each pseudo-SNP (haploblock allele), i.e.:

(5) $$VEM{\%}_{i}=\frac{V\left(g_{i}\right)}{\sigma_{g}^{2}}\times 100=\frac{2p_{i}\left(1-p_{i}\right){\hat{\alpha }}_{i}^{2}}{\sigma_{g}^{2}}\times 100$$

where $VEM{\%}_{i}$ is the percentage of the total additive genetic variance explained by the *i*th pseudo-SNP, $V\left(g_{i}\right)\;$ is the additive genetic variance explained by the *i*th pseudo-SNP, ${\hat{\alpha }}_{i}^{2}$ is the square of the estimated allelic substitution effect, and the other components of the formula were previously defined. As the pseudo-SNPs were the alleles present in the haploblock loci, the percentage of the variance explained by each haploblock was computed as:

(6) $$VEH{\%}_{j}=\overset{n_{j}}{\underset{i=1}{\sum }}VEM{\%}_{ij}$$

where $VEH{\%}_{j}$ is the percent of the total additive genetic variance explained by the *j*th haploblock, $n_{j}$ is the number of haplotype alleles (pseudo-SNPs) present in the *j*th haploblock, and $VEM{\%}_{ij}$ are the variances explained by the *i*th pseudo-SNPs present within the *j*th haploblock. 

### 2.5. Weighted Single-Step GWAS with Haplotypes

The WssGWAS uses, for simplicity, an interactive process to estimate the weights for the markers. The first step in the WssGWAS is to perform the ssGWAS (i.e., considering equal weights for all markers). The procedure consists of three steps, starting with the prediction of GEBV from ssGBLUP, deriving the weights for the markers by back-solving SNP effects, and including the weights into the  D matrix to construct the G matrix that is combined with the pedigree-based relationship (resulting in the **H**) in the next steps in an iterative process [19]. For details of the full algorithm, please see Wang et al. [19]. The POSTGSf90 software [25] was used for back-solving the GEBV to pseudo-SNP effects and weights, and the non-linear A (NLA) method [32] was used to obtain the weights. The NLA weighting method was used because it has better statistical properties (i.e., convergence of the GEBV accuracies and control over extreme weight values) than the original method proposed by Wang et al. [19], as suggested by Fragomeni et al. [34]. In addition to the first GEBV prediction and association (ssGWAS), two iterations in WssGBLUP were completed to provide high accuracy and low bias of the GEBV used in the WssGWAS [19,34], and the results of these two iterations were compared to ssGWAS. The same genetic model, **H** matrix construction, and percentage of the variances explained by each pseudo-SNP and haploblock loci presented in the topic 2.4 were also used for the WssGWAS.

### 2.6. Scenarios Evaluated

In addition to the three initial scenarios regarding the ssGWAS method (i.e., ssGWAS, 2nd iteration WssGWAS, and 3rd iteration WssGWAS), alternative approaches were used when constructing the G matrix. The scenarios included the construction of haplotypes based on: (1) the LD thresholds of 0.15 (H0.15), 0.50 (H0.50), and 0.80 (H0.80), as mentioned in the topic 2.3.; and (2) considering only haplotypes or the haplotypes and non-LD-clustered SNP (NCSNP) from those same LD thresholds together in the construction of a single G matrix. The NCSNP were SNP not assigned to any block during the haploblock construction with a determined LD threshold. These scenarios with NCSNP were also evaluated to avoid a possible loss of ability in dissecting the genetic variation by losing the markers outside blocks described by Li et al. [35], and the use of a single G constructed with NCSNP and haplotypes provides greater GEBV accuracy and lower bias [18]. Therefore, six scenarios in the context of the G construction were investigated: H0.15, H0.50, H0.80, and NCSNP and haplotypes from blocks with LD thresholds of 0.15, 0.50, and 0.80 (NCSNP_H0.15, NCSNP_H0.50, and NCSNP_H0.80, respectively). All scenarios are described in Table 1. 

### 2.7. Empirical Selection of the Candidate Regions for Further Investigation

The percentage of the total additive genetic variance explained by the markers was evaluated to determine the regions to be further investigated. In this context, the moments of the distribution from the percentage of the variance explained by the markers were estimated first, and then the skewness-kurtosis plot proposed by Cullen and Frey [36] was utilized to select candidate distributions. The skewness-kurtosis plot [36] presented values for the moments of common distributions (Normal, Uniform, Exponential, Logistic, Beta, Log-normal, Gamma, and Weibull) to select the distribution that better fit the data. The R package “fitdistplus” [37] was used to evaluate the skewness-kurtosis plot using 10,000 bootstrap samples to choose candidate distributions for the percentage of the variance explained by the markers. The Beta or Gamma distributions were chosen based on the skewness-kurtosis plot to be candidate distributions (not the same distribution for all scenarios; Supplementary File S1). Thereafter, the theoretical and empirical probability density function (PDF), cumulative probability function (CDF), and QQ and PP plots for the Beta and Gamma distributions were evaluated. The Beta and Gamma distributions fit the data well and were used to determine the candidate regions to be further investigated. The markers that were further investigated explained the largest percentage of the additive variance and were present in the quantile 0.001% of the fitted distribution, i.e., considered to be the most relevant genomic regions (top 0.001%). To obtain candidate regions for YT, the quantile 0.001% for the top markers that explained most of the additive variance was empirically defined because it is an extreme tail of the distribution. Using greater thresholds, e.g., 0.01 or 0.05%, only increased the number of genes and QTL related to more general functions and biological processes (Supplementary Files S2 and S3).

### 2.8. Functional Analyses

The top 0.001% genomic regions for YT were used to find genes and overlapping QTL using the Biomart tool from Ensembl (www.ensembl.org/biomart/martview/ad1112a783c0e0ae22e6572189d5bead, accessed on 14 August 2021) and the Animal QTLdb [38] (www.animalgenome.org/cgi-bin/QTLdb/BT/index, accessed on 14 August 2021), respectively. These analyses were done based on the latest ARS-UCD1.2 bovine genome assembly [39,40]. Positional candidate genes overlapping with the top genomic regions were functionally annotated using the DAVID platform (https://david.ncifcrf.gov/home.jsp, accessed on 15 August 2021) with focus on the Gene Ontology biological processes (GO_BP) and metabolic pathways from the Kyoto Encyclopedia of Genes and Genomes (KEGG) using the default options.

## 3. Results

### 3.1. Statistics from Haplotype Blocking

The number of non-clustered SNPs (i.e., SNPs out of the LD-blocks) ranged from 20,849 to 36,881 when using the LD thresholds of 0.15 and 0.80, respectively (Table 2). The number of clustered SNPs ranged from 5822 to 21,784 with LD thresholds of 0.80 and 0.15, respectively. Similar to the number of clustered SNPs, the number of blocks decreased with higher LD thresholds, varying from 2721 to 9634 when 0.80 and 0.15 were used as LD thresholds, respectively. As previously defined, the minimum number of SNPs in blocks were 2 for all LD thresholds, whereas the maximum number of SNPs in blocks was equal to 9 for the LD threshold of 0.15 and 7 for 0.50 and 0.80. On average, smaller blocks were obtained when the LD threshold was 0.80 (0.030 Mb), and bigger blocks were obtained with an LD threshold of 0.15 (0.035 Mb). The minimum block size was 65 bp with the LD thresholds of 0.15 and 0.50, and 84 bp with the LD threshold of 0.80. The maximum block size ranged from 0.160 Mb to 0.201 Mb with the LD thresholds of 0.80 and 0.15, respectively. The number of pseudo-SNPs (unique haplotype alleles) before QC varied between 12,877 and 56,734 with LD thresholds of 0.80 and 0.15, respectively. After QC, the number of pseudo-SNPs ranged from 11,389 to 44,559, respectively, in the same LD thresholds. The number of NCSNP and pseudo-SNPs before QC, considering them all as genomic markers, ranged between 49,688 and 77,583 with LD thresholds of 0.80 and 0.15, respectively. After QC, the number of NCSNP and pseudo-SNPs ranged from 48,227 to 65,435, respectively, in the same LD thresholds.

### 3.2. Traditional and Weighted Single-Step GWAS Fitting Only Haplotypes

**Figure 1:**
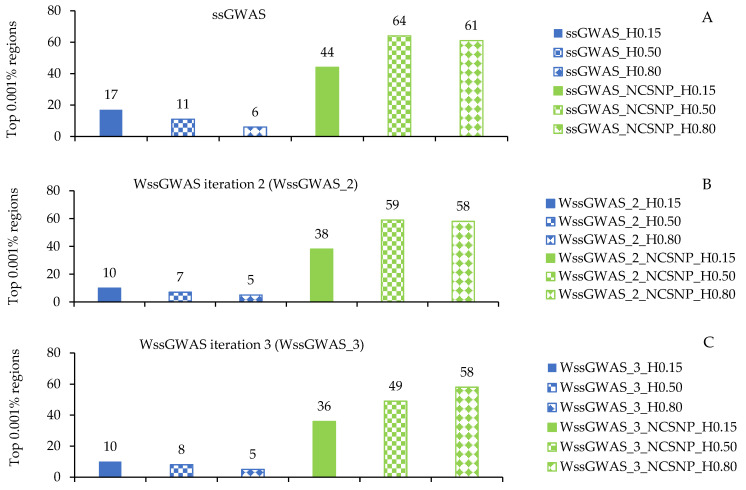
Number of top 0.001% genomic regions for yearling temperament in American Angus cattle found by non-weighted single-step GWAS (ssGWAS) (**A**) and weighted ssGWAS (WssGWAS) in the second (**B**) and third (**C**) iterations. H0.15, H0.50, and H0.80: only haplotypes from blocks with linkage disequilibrium (LD) thresholds of 0.15, 0.50, and 0.80, respectively; NCSNP_H0.15, NCSNP_H0.50, and NCSNP_H0.80: non-clustered SNPs (NCSNP) and haplotypes from blocks with LD thresholds of 0.15, 0.50, and 0.80, respectively. The column colors highlight not including (blue) or including NCSNP (green). The column filling highlights different LD thresholds (0.15, 0.50, and 0.80 with a solid, square, and diamond filling, respectively).

**Figure 2:**
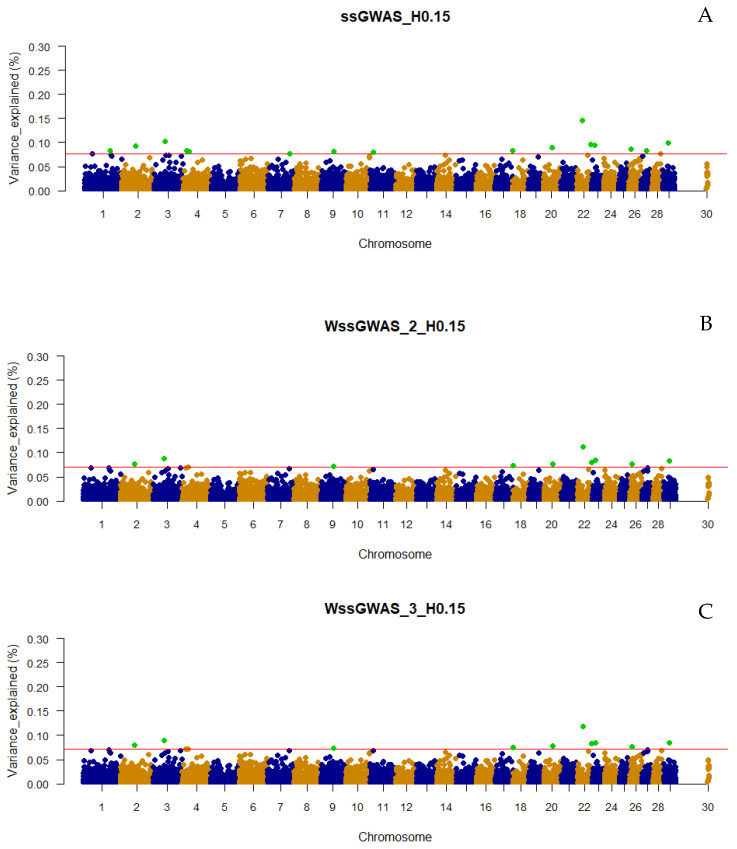
Manhattan plot of the percentage of the total additive genetic variance explained by haplotypes from haploblocks built with a linkage disequilibrium threshold of 0.15 for yearling temperament in American Angus cattle using single-step GWAS (ssGWAS_H0.15; **A**) and weighted single-step GWAS in the second (WssGWAS_2_H0.15; **B**) and third (WssGWAS_3_H0.15; **C**) iterations. Green points highlighted above the red horizontal line are the top 0.001% of markers that explained greater percentages of the total additive genetic variance for YT. The X-chromosome (PAR region) is represented by the chromosome 30.

**Figure 3:**
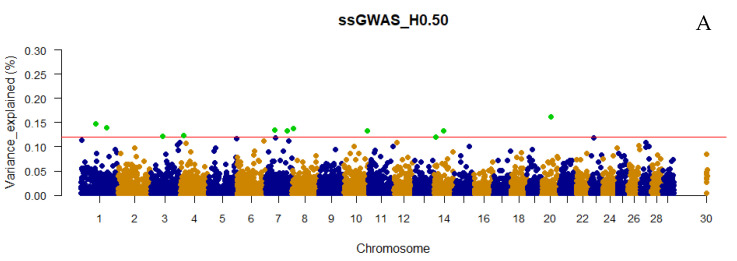

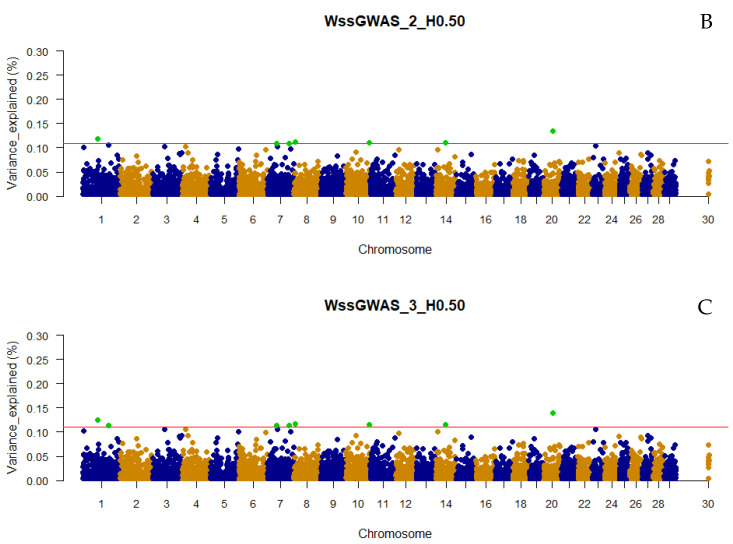
Manhattan plot of the percentage of the total additive genetic variance explained by haplotypes from haploblocks built with a linkage disequilibrium threshold of 0.50 for yearling temperament in American Angus cattle using single-step GWAS (ssGWAS_H0.50; **A**) and weighted single-step GWAS in the second (WssGWAS_2_H0.50; **B**) and third (WssGWAS_3_H0.50; **C**) iterations. Green points highlighted above the red horizontal line are the top 0.001% markers that explained greater percentages of the total additive genetic variance for YT. The X-chromosome (PAR region) is represented by the chromosome 30.

**Figure 4:**
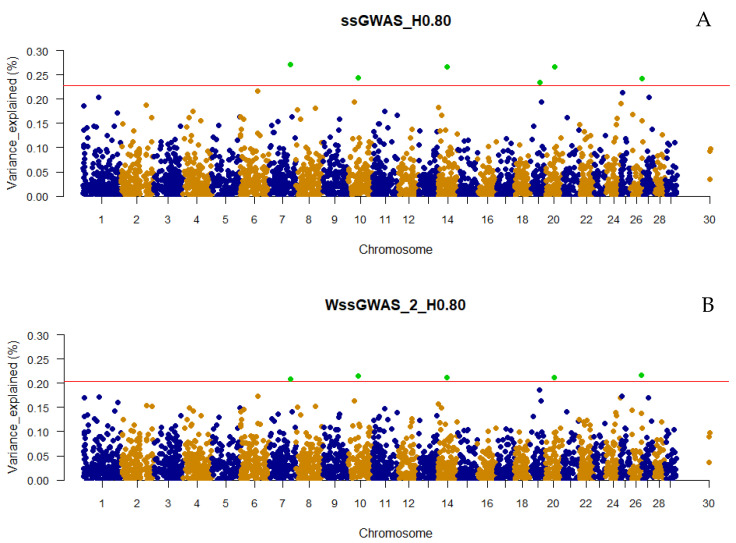

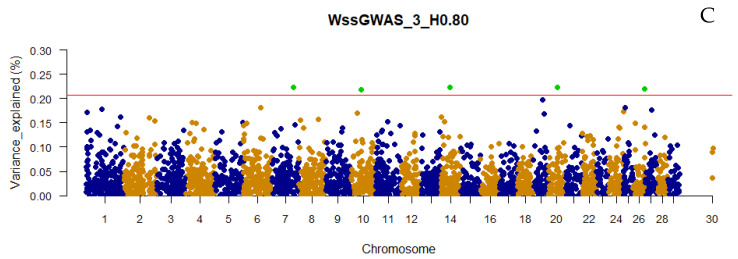
Manhattan plot of the percentage of the total additive genetic variance explained by haplotypes from haploblocks built with a linkage disequilibrium threshold of 0.80 for yearling temperament in American Angus cattle using single-step GWAS (ssGWAS_H0.80; **A**) and weighted single-step GWAS in the second (WssGWAS_2_H0.80; **B**) and third (WssGWAS_3_H0.80; **C**) iterations. Green points highlighted above the red horizontal line are the top 0.001% markers that explained greater percentages of the total additive genetic variance for YT. The X-chromosome (PAR region) is represented by the chromosome 30.

The number of top 0.001% genomic regions ranged from 5 (WssGWAS_2_H0.80 and WssGWAS_3_H0.80) to 17 (ssGWAS_H0.15) when only haplotypes were used in the ssGWAS (Figure 1). Despite the number of top 0.001% genomic regions identified being slightly lower in WssGWAS compared to ssGWAS scenarios (Figure 1), all top genomic regions highlighted by WssGWAS scenarios (Figure 2, Figure 3 and Figure 4) were present in the ssGWAS results regardless of the iteration within LD thresholds. For this reason, functional annotation was performed only for ssGWAS results (Supplementary File S2). The top haplotypes for YT were located across 14 chromosomes (BTA1, BTA2, BTA3, BTA4, BTA7, BTA9, BTA11, BTA18, BTA20, BTA22, BTA23, BTA26, BTA27, and BTA29; Figure 2) for the ssGWAS_H0.15 scenario, which presented top genomic regions more spread out than the other scenarios using only haplotypes. A total of 11, 7, and 5 candidate genes overlapped with the top 0.001% genomic regions identified by ssGWAS_H0.15, ssGWAS_H0.50, and ssGWAS_H0.80, respectively, whereas 67, 55, and 30 QTL overlapped in these same scenarios, respectively. No associations were observed in the X chromosome (PAR region) when fitting only haplotypes.

### 3.3. Traditional and Weighted Single-Step GWAS Fitting Haplotype Blocks and Non-Clustered SNP

**Figure 5:**
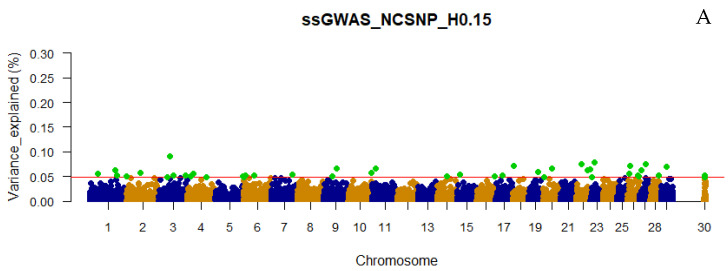

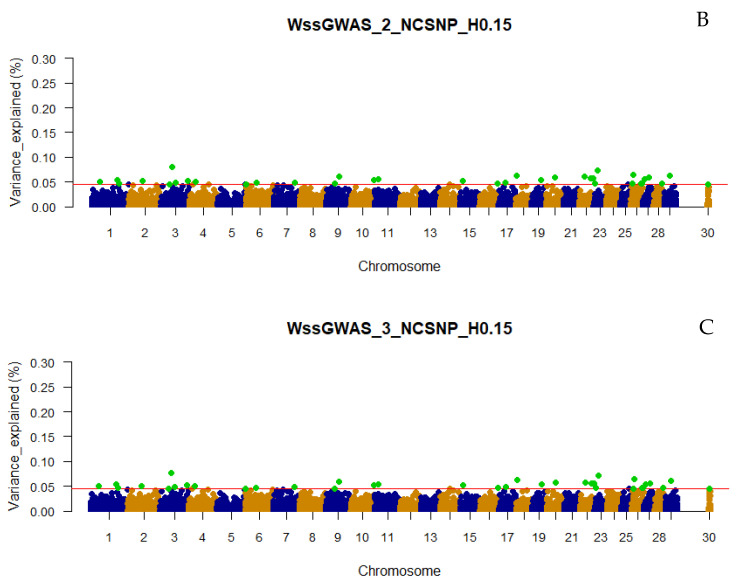
Manhattan plot of the percentage of the total additive genetic variance explained by non-clustered SNPs and haplotypes from haploblocks built with a linkage disequilibrium threshold of 0.15 for yearling temperament in American Angus cattle using single-step GWAS (ssGWAS_NCSNP_H0.15; **A**) and weighted single-step GWAS in the second (WssGWAS_2_NCSNP_H0.15; **B**) and third WssGWAS_3_NCSNP_H0.15; **C**) iterations. Green points highlighted above the red horizontal line are the top 0.001% markers that explained greater percentages of the total additive genetic variance for YT. The X-chromosome (PAR region) is represented by the chromosome 30.

**Figure 6:**
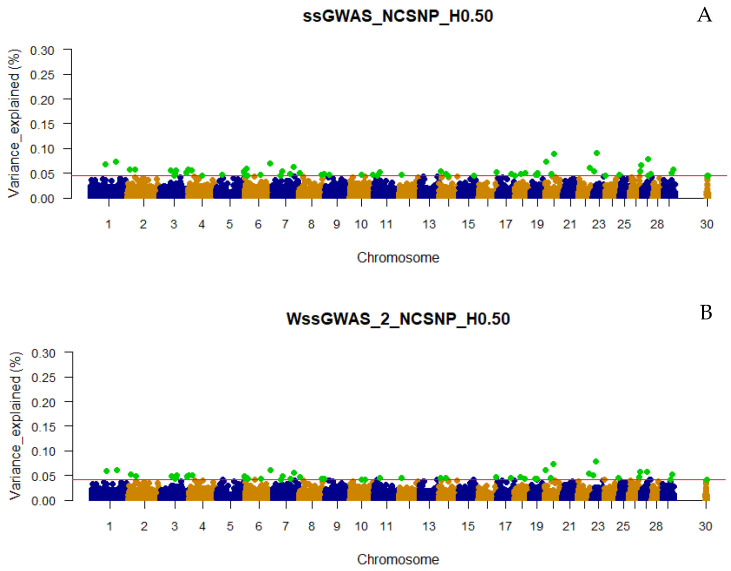

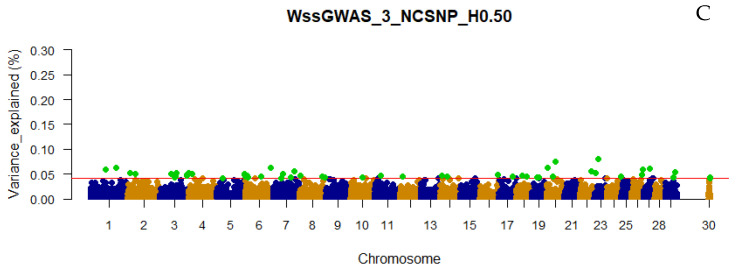
Manhattan plot of the percentage of the total additive genetic variance explained by non-clustered SNPs and haplotypes from haploblocks built with a linkage disequilibrium threshold of 0.50 for yearling temperament in American Angus cattle using single-step GWAS (ssGWAS_NCSNP_H0.50; **A**) and weighted single-step GWAS in the second (WssGWAS_2_NCSNP_H0.50; **B**) and third (WssGWAS_3_NCSNP_H0.50; **C**) iterations. Green points highlighted above the red horizontal line are the top 0.001% markers that explained greater percentages of the total additive genetic variance for YT. The X-chromosome (PAR region) is represented by the chromosome 30.

**Figure 7:**
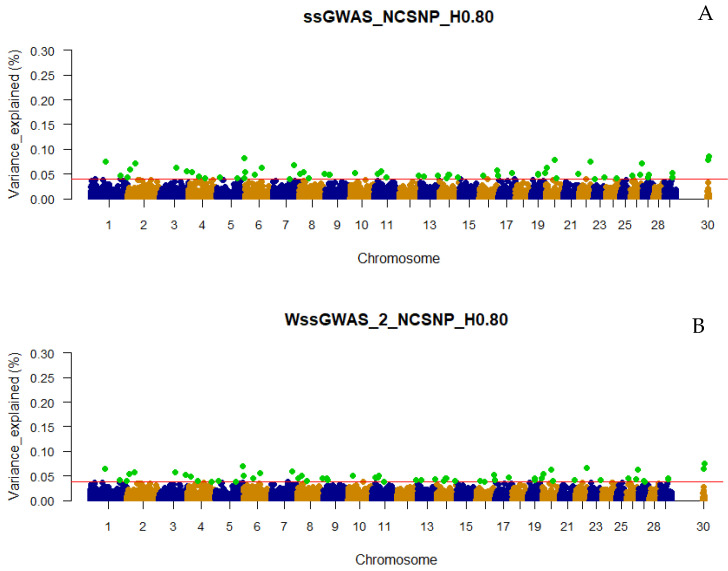

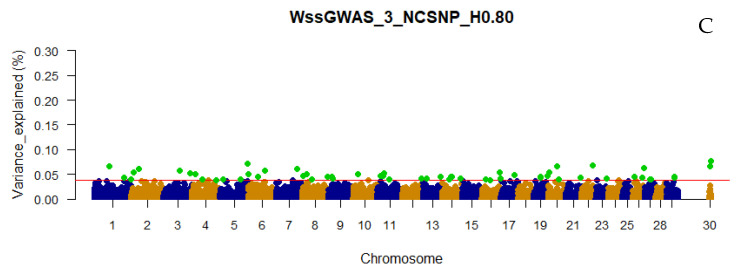
Manhattan plots of the percentage of the total additive genetic variance explained by non-clustered SNPs and haplotypes from haploblocks built with a linkage disequilibrium threshold of 0.80 for yearling temperament in American Angus cattle using single-step GWAS (ssGWAS_NCSNP_H0.80; **A**) and weighted single-step GWAS in the second (WssGWAS_2_NCSNP_H0.80; **B**) and third (WssGWAS_3_NCSNP_H0.80; **C**) iterations. Green points highlighted above the red horizontal line are the top 0.001% markers that explained greater percentages of the total additive genetic variance for YT. The X-chromosome (PAR region) is represented by the chromosome 30.

Including the NCSNP in the association analyses resulted in more top regions being captured by the haploblocks from all LD thresholds and under both ssGWAS or WssGWAS approaches. The number of top 0.001% genomic regions ranged between 36 to 64 in the WssGWAS_3_NCSNP_H0.15 and ssGWAS_NCSNP_H0.50 scenarios, respectively (Figure 1). Similar to what was observed when using only haplotypes, all top markers (pseudo-SNPs and NCSNP) highlighted by WssGWAS were present in the ssGWAS scenarios (Figure 5, Figure 6 and Figure 7), which also had more top markers in all LD thresholds (Figure 1). Functional annotation was performed only for ssGWAS in the scenarios including the NCSNP for the same reason previously described (Supplementary File S3). Different from what was observed in the ssGWAS using only haplotypes, the top 0.001% genomic regions were well distributed in the bovine chromosomes, including the PAR region of the X chromosome, with all LD the three thresholds used to create the haploblocks. The additional number of top 0.001% genomic regions using haplotypes and NCSNP together also implied more annotated genes and overlapping QTL (36, 54, and 35 genes and 159, 169, and 157 QTL for the ssGWAS_NCSNP_H0.15, ssGWAS_NCSNP_H0.50, and ssGWAS_NCSNP_H0.80, respectively; Supplementary File S3).

### 3.4. Overlapping Genomic Regions among Methods and Functional Analyses

#### 3.4.1. Overlapping Markers 

**Figure 8:**
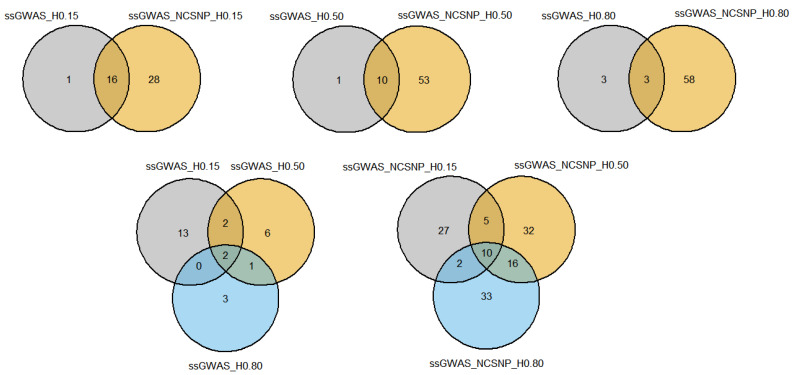
Venn diagrams showing the number of markers overlapping among different single-step genome-wide association studies (ssGWAS) with haplotypes and non-clustered SNPs. ssGWAS_H0.15, ssGWAS_H0.50, and ssGWAS_H0.80: ssGWAS using only haplotypes from blocks with linkage disequilibrium (LD) thresholds of 0.15, 0.50, and 0.80, respectively; ssGWAS_NCSNP_H0.15, ssGWAS_NCSNP_H0.50, and ssGWAS_NCSNP_H0.80: ssGWAS using non-clustered SNPs and haplotypes from blocks with LD thresholds of 0.15, 0.50, and 0.80, respectively.

Considerable overlap among many of the top markers was found by the different ssGWAS methods. The majority of the top markers identified by ssGWAS using only haplotypes were also present in the scenarios using NCSNP and haplotypes together (Figure 8; Supplementary File S4). Only one top haplotype in the ssGWAS_H0.15 and ssGWAS_H0.50 scenarios and three haplotypes in the ssGWAS_H0.80 were found exclusively when using haplotypes, i.e., all other markers were captured by their respective scenarios using NCSNP. NCSNP allowed us to identify unique top regions within each LD threshold used to build the haploblocks, with ssGWAS_NCSNP_H0.15, ssGWAS_NCSNP_H0.50, and ssGWAS_NCSNP_H0.80 detecting 28, 53, and 58 additional top regions than their respective scenarios using only haplotypes. When comparing all scenarios fitting only haplotypes, 2 regions were common between ssGWAS_H0.15, ssGWAS_H0.50, and ssGWAS_H0.80, at 89 Mb on the BTA7 and 38 Mb on BTA20. Furthermore, top regions were specifically identified in each scenario with different LD thresholds when only haplotypes were used in the ssGWAS, with 13, 6, and 3 haplotypes identified exclusively in ssGWAS_H0.15, ssGWAS_H0.50, and ssGWAS_H0.80, respectively. A total of 10 markers were found in common between all methods, including NCSNP in ssGWAS. The scenarios with NCSNP and haplotypes together also presented markers exclusively found by specific LD thresholds, with 27, 33, and 32 markers identified by the ssGWAS_NCSNP_H0.15, ssGWAS_NCSNP_H0.50, and ssGWAS_NCSNP_H0.80 scenarios, respectively. The 2 common regions between all ssGWAS scenarios using haplotypes were also present within the 10 common regions when fitting NCSNP and haplotypes together. 

#### 3.4.2. Overlapping Genes 

**Figure 9:**
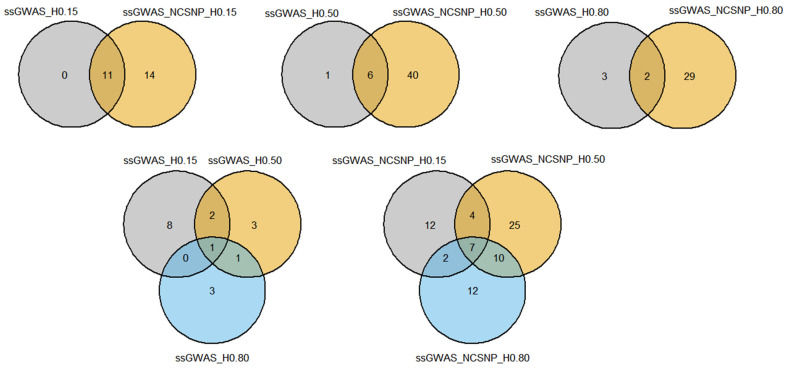
Venn diagrams showing the number of genes overlapping among different single-step genome-wide association studies (ssGWAS) with haplotypes and non-clustered SNPs. ssGWAS_H0.15, ssGWAS_H0.50, and ssGWAS_H0.80: ssGWAS using only haplotypes from blocks with linkage disequilibrium (LD) thresholds of 0.15, 0.50, and 0.80, respectively; ssGWAS_NCSNP_H0.15, ssGWAS_NCSNP_H0.50, and ssGWAS_NCSNP_H0.80: ssGWAS using non-clustered SNPs and haplotypes from blocks with LD threshold of 0.15, 0.50, and 0.80, respectively.

The overlapping markers among scenarios were also present in genes shared between ssGWAS strategies using only haplotypes built with different LD thresholds and including the NCSNP. All annotated genes identified based on the ssGWAS_H0.15 scenario were also identified by ssGWAS_NCSNP_H0.15 (Figure 9; Supplementary File S5). Only one (*COMMD10*) and three (*NID2*, *PLXDC1*, and *DOCK1*) annotated genes were identified exclusively by the ssGWAS_H0.50 and ssGWAS_H0.80, respectively, compared to the scenarios including NCSNP. Considering all ssGWAS scenarios using only haplotypes, a unique gene (*SPEF2*) was found by the three scenarios. Seven genes (*5S_rRNA*, *UMAD1*, *PTPRC*, *SPEF2*, *CACNA2D3*, *HMGCLL1*, and *MGMT*) were identified by all three ssGWAS scenarios, including NCSNP, and the unique gene overlapped by all three scenarios, including haplotype-only methods, was also present among them.

#### 3.4.3. Functional Analyses

The results from the functional analyses are presented in Table 3 and Supplementary Files S6 and S7. No clusters were significantly enriched using default parameters in the DAVID platform. For simplicity, only the candidate genes, GO_BP, and KEGG metabolic pathways from the Functional Annotation tables provided by DAVID for key candidate genes with direct or indirect implications in behavioral or docility traits such as those related to the nervous system were presented. Details about all the overlapping genes are presented in the Supplementary Files S2 and S3, and the Functional Annotation tables for all genes are presented in Supplementary Files S6 and S7. 

The gene *ATXN10* (position 116 Mb on BTA5) was annotated in the GO:0031175 term, which is associated with neuron projection development. The bta05010 KEGG metabolic pathway was annotated for the gene *ADAM10* (position 51 Mb on the BTA10) and is related to the Alzheimer disease. The gene *VAX2* (position 13 Mb on BTA 11) was annotated in the GO:0007409, GO:0007601, GO:0030900, GO:0048048, and GO:0060041 biological processes, which are axonogenesis, visual perception, forebrain development, embryonic eye morphogenesis, and retina development in camera-type eye, respectively. The biological processes in GO:00076605 and GO:0042472 included the gene *ATP6V1B1* (position 13 Mb on BTA11) and are related to sensory perception of sound and inner ear morphogenesis, respectively. GO:0060325 is related to face morphogenesis and includes the *CRISPLD1* gene (position 38 Mb on the BTA14). The *CAPRIN1* gene (position 64 Mb on the BTA15) was annotated in GO:0050775 and GO0061003, which are related to the positive regulation of dendrite and dendrite spine morphogenesis, respectively. Two biological processes included the *FA2H* gene (position 2 Mb on BTA18), which are GO:0032286 and GO:0032287, known to be related to central and peripheral nervous system myelin maintenance, respectively. The gene *SPEF2* (position 38 Mb on BTA20) was annotated in GO:0048702, GO:0048854, and GO:0069541, which are related to the embryonic neurocranium, brain morphogenesis, and respiratory system development. Four biological processes related to the nervous system included the *PLXNA1* gene, which are GO:0021785, GO: 0048841, GO:1902287, and GO:1990138, known to be related to branchiomotor neuron axon guidance, regulation of axon extensions involved in guidance, the semaphorin-plexin signaling pathway involved in axon guidance, and neuron projection extension, respectively. The *PLXNA1* gene was also annotated in the bta04360 KEGG pathway, which is related to axon guidance. Finally, the gene *CACNA2D3* was annotated in the bta04921 KEGG pathway and is related to the oxytocin signaling pathway.

### 3.5. QTL Overlapping with the Top 0.001% Markers for Yearling Temperament

**Figure 10:**
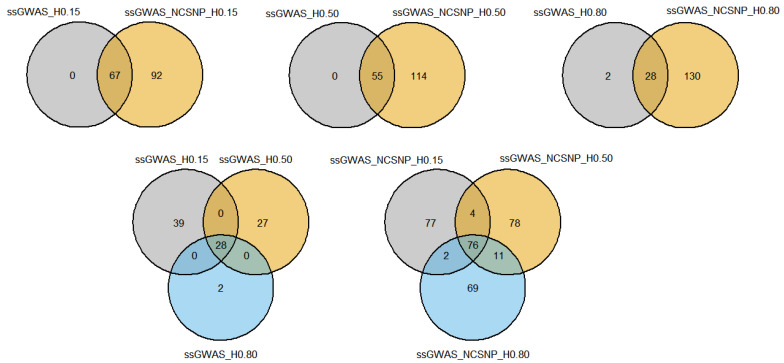
Venn diagrams showing the number of QTL overlapping among different single-step genome-wide association studies (ssGWAS) with haplotypes and non-clustered SNPs. ssGWAS_H0.15, ssGWAS_H0.50, and ssGWAS_H0.80: ssGWAS using only haplotypes from blocks with linkage disequilibrium (LD) thresholds of 0.15, 0.50, and 0.80, respectively; ssGWAS_NCSNP_H0.15, ssGWAS_NCSNP_H0.50, and ssGWAS_NCSNP_H0.80: ssGWAS using non-clustered SNPs and haplotypes from blocks with LD thresholds of 0.15, 0.50, and 0.80, respectively.

Similar to what was observed with the genes, the overlapping markers across scenarios also implied in QTL found in more than one scenario. All QTL identified using only haplotypes with LD thresholds of 0.15 and 0.50 were captured when the NCSNP were used in the ssGWAS, while only two QTL were found by ssGWAS_H0.80 and not by ssGWAS_NCSNP_H0.80 (Figure 10; Supplementary File S8). Using different LD thresholds to create the haploblocks resulted in specific QTL captured by the different block structures, with 39, 27, and 2 QTL identified exclusively by ssGWAS_H0.15, ssGWAS_H0.50, and ssGWAS_H0.80, respectively. Adding NCSNP in ssGWAS also resulted in QTL captured by specific scenarios regarding the LD threshold to create the haploblocks, with 77, 78, and 69 QTL identified exclusively by ssGWAS_NCSNP_H0.15, ssGWAS_NCSNP_H0.50, and ssGWAS_NCSNP_H0.80, respectively. A total of 28 QTL were identified by all 3 scenarios using haplotypes only and were also present among the 76 QTL identified by all 3 scenarios including NCSNP and haplotypes. 

**Figure 11:**
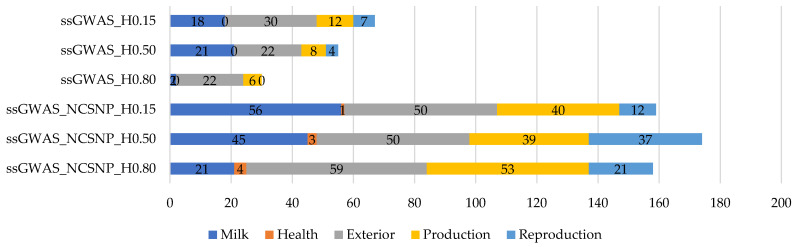
Absolute number of quantitative trait loci (QTL) by class overlapping with the top 0.001% markers for yearling temperament in American Angus cattle using the single-step GWAS fitting only haplotypes or non-clustered SNPs and haplotypes. ssGWAS_H0.15, ssGWAS_H0.50, and ssGWAS_H0.80: ssGWAS using only haplotypes from blocks with linkage disequilibrium (LD) thresholds of 0.15, 0.50, and 0.80, respectively; ssGWAS_NCSNP_H0.15, ssGWAS_NCSNP_H0.50, and ssGWAS_NCSNP_H0.80: ssGWAS using non-clustered SNPs and haplotypes from blocks with LD thresholds of 0.15, 0.50, and 0.80, respectively.

The QTL identified by the scenarios evaluated in this research belong to the classes “Milk”, “Health”, “Exterior”, “Production”, and “Reproduction” (Figure 11). The majority of the QTL identified in each scenario were related to the class “Exterior”, except for the ssGWAS_NCSNP_H0.15 scenario (for this, the class “Milk” contained the majority of the QTL). “Milk” was the class most often found among QTL after the class “Exterior”, followed by “Production”, “Reproduction”, and, lastly, “Health”. The QTL from the class “Health” were found only when NCSNP were included in the ssGWAS. 

## 4. Discussion

We have investigated the genomic architecture of YT using large phenotypic and genomic datasets from American Angus cattle. Different haploblock structures obtained by three LD thresholds to create blocks were used while including or excluding the NCSNP in order to capture different genes and QTL structures that could affect YT in American Angus. 

### 4.1. Empirical Selection of the Candidate Genomic Regions

All the steps to obtain the top 0.001% genomic regions for YT presented in the Section 2.7 were done because of the lack of a statistical method to properly test for significance of markers using ssGWAS for threshold traits. The approximated *p*-values for the markers in the ssGWAS approach was proposed under normality assumptions [41] and are not available for threshold traits. 

Only defining a fixed value to select the candidate regions, e.g., 0.50 or 1.00% of the additive variance, as most of the GWAS studies employ, would not be an equivalent comparison with scenarios using different marker densities (e.g., number of pseudo-SNPs from haploblocks with different LD thresholds or including or excluding the NCSNP; Table 2), as the percentage of the additive genetic variance explained by each marker is inversely proportional to the number of markers (Figure 2, Figure 3, Figure 4, Figure 5, Figure 6 and Figure 7). In addition, it is possible that markers with a percentage of the total additive genetic variance smaller than 0.50% are biologically associated with the traits of interest. Aguilar et al. [41] presented significant *p*-values for markers that explained ~0.10% (similar to some scenarios in this research) of the additive variance for the birth weights in American Angus using more than one million phenotypes and approximately 1.4 K genotyped sires with phenotyped progeny. 

It is well known that the significance and the additive variance of the markers are dependent on the sample size and population structure, as well as the allelic frequencies, additive values of the QTL tagged by the marker, LD between marker and QTL (assuming that the QTL is not the marker), and the accuracy of the phenotypic information [20,41]. The interaction of these components is complex and makes it difficult to define a threshold for selecting candidate markers based only on the percentage of the total additive genetic variance explained by the markers (or genomic windows). Various key candidate genes associated with GO_BP and KEGG pathways were found. These may play an important role in YT, as well as previously reported QTL. Nevertheless, further studies are needed to evaluate the method to obtain the top regions in this study, as defining false or true positive associations is not straightforward when using real datasets [20].

### 4.2. Additive Genetic Variance Explainded by Genomic Regions across Scenarios 

It was not surprising to not find regions explaining more than 1% of the total additive genetic variation for YT, since behavioral traits are highly polygenic [4,5,42]. Overall, the variances explained by each unique genomic region (i.e., haplotype or SNP) in all scenarios were small and distributed across the chromosomes (Figure 2, Figure 3, Figure 4, Figure 5, Figure 6 and Figure 7 and Supplementary Files S2 and S3), highlighting the polygenic nature of YT, which was also reported by Alvarenga et al. [5] using only SNPs. Alvarenga et al. [5] found 11 genomic windows considering five adjacent SNPs explaining about 3.33% of the total additive genetic variance for YT, and these regions were distributed across the bovine autosome chromosomes, similar to the results in the present study.

The fact that WssGWAS did not increase the variance explained by major genomic regions gives further evidence of the polygenic nature of YT (Figure 2, Figure 3, Figure 4, Figure 5, Figure 6 and Figure 7). Furthermore, a high correlation was observed between the GEBVs from ssGWAS and WssGWAS methods (greater than 0.98; Supplementary File S9) regardless of the LD threshold used to create the haploblocks (i.e., 0.15, 0.50, 0.80). An assumption in ssGBLUP, and consequently ssGWAS, is that all markers explain similar and small proportion of the total additive genetic variance. Thus, WssGBLUP was developed in order to minimize this effect by giving priorities to some markers with potentially greater effects [19,23]. In this case, one would expect that the genomic regions with a higher impact on the variance of the trait would present higher peaks based on WssGWAS compared to ssGWAS. Substantial changes in the GEBVs, higher accuracies, and lower bias of genomic predictions are also expected with WssGBLUP for those traits with major genes, i.e., regions that should receive greater weight [19,23,43]. Hence, due to the evidence of this polygenic nature, the results from the ssGWAS scenarios were used to identify candidate genes and QTL. 

### 4.3. Weighting Method in the Single-Step GWAS 

In early stages in this study, the weighting method proposed by Wang et al. [19] in the WssGWAS beyond the NLA was attempted for comparison purposes. However, problems during the iterative process using the Wang et al. [19] method regarding the inversion of the G matrix used, and it was not possible to generate results for the second and third iterations in the majority of the scenarios. The NLA method proposed by VanRaden [32] is conservative in the shrinking process due to the limits in the maximum changes in the weights applied [34]. However, it is relevant to point out that Wang et al. [19]’s assumptions on marker weights rely only on the SNP effect and allelic frequency. The weights considered by Wang et al. [19] in this study had a wider range (from ~0.5 to ~28), and are most likely less accurate, considering the polygenic nature of YT, compared to the NLA approach (~0.9 to ~1.6) after the first iteration of the weighting process (ssGWAS). 

This problem using the Wang et al. [19] method could be a result of the haplotypes being more polymorphic than SNPs [10,11,12,18], so the broader range in the weights could be due to the larger number of alleles in the same region compared to SNP tracking the actual additive genetic effect. As the GEBV accuracy can decline and bias can increase during the iterative process using the Wang et al. [19] method [34], consequently, the SNP effects and variances could be less accurate and more biased. Therefore, it is recommended to use NLA weights for WssGWAS purposes because it results in more robust weighting values.

### 4.4. Genes and QTL Overlapping the Top Genomic Regions 

Some of the genes present in the top regions for YT were previously reported in other studies to be associated with behavioral traits. The genes *7SK*, *U6*, and *5S_rRNA* were associated with behavior in cattle by Alvarenga et al. [5,8]. The *7SK* gene is a small nuclear RNA gene that belongs to a class of subunits spread in the bovine genome and that already had a unit previously associated with fertility traits [44]. The *U6* gene is a gene found in more than one BTA and also belongs to a small nuclear RNA class which was previously related to temperament [45], maternal behavior [46], and sucking reflex [47] in cattle. Beyond small nuclear RNA genes, small nucleolar RNA genes (*SNORD25*, *SNORD26*, and *SNORD27*) and long non-coding RNA genes (*lncRNA*) were found, but no previous functions related to behavior, production, reproduction, or health were found. Beyond the presence in a top region (position 41 Mb on BTA29) for YT, QTL related to milk production and reproduction (two and 11, respectively) were also annotated in the same top region (block_81_chr_29; Supplementary File S3) where those small nuclear and long non-coding RNA genes were found. The RNA genes codify transcriptional factors required for splicing [48] so they can be involved in many different processes affecting gene expression. The *5S_rRNA* gene is an RNA ribosomal gene that has also been reported to be involved in milk, meat quality, and carcass traits [49]. 

Despite the small nuclear, nucleolar, and ribosomal RNA genes found, the majority of the genes annotated in the top regions are protein coding (Supplementary Files S2 and S3). Most of the protein-coding genes are related to a broad range of more basal functions (e.g., glucose metabolism, pH regulation, transcription; Supplementary Files S6 and S7) according to the Functional Annotation Table from the DAVID platform, which could explain the absence of significant clusters. The presence of many different biological processes affecting YT is expected, as behavioral traits involve many functions between the perception of a stimulus and the reaction to a specific stress or situation [50,51]. Nevertheless, genes present in GO_PB and KEGG pathways related to nervous system development, mental disorders, stimuli perception, and respiratory development (Table 3) were also found, and these functions are related to behavioral stress responses [1,4,51]. 

The ataxin 10 gene (*ATXN10*), annotated in the neuron projection development GO_BP, was previously associated with longevity traits in Chinese Holstein cattle [52]. Longevity is a productive trait that is affected to some degree by the cattle’s temperament, as aggressiveness is an undesirable trait and is a culling criterion in American Angus [53]. The ADAM metallopeptidase domain 10 (*ADAM10*) gene was already identified as a biomarker for Alzheimer’s disease in humans, with functions related to the cleavage amyloid precursors that act during the inflammation process of senile plaques [54]. In cattle, *ADAM10* was associated with tick resistance, with its importance in the inflammation process being the most likely reason [55]. 

The ventral anterior homeobox 2 (*VAX2*) gene was annotated for biological processes related to visual perception, axonogenesis, and forebrain development, which are very important processes in behavioral responses to environmental stimuli [1]. An association with fertility-related traits was also previously reported for the *VAX2* gene in cattle [56]. Beyond visual perception, auditive-related biological processes (sensory perception of sound and inner ear morphogenesis) were annotated for the gene ATPase H^+^ transporting V1 subunit B1 (*ATP6V1B1*), indicating that vision and auditory senses are among the main functions influencing YT. The animal’s perception of the area around it is involved in behavioral responses [1], so that the presence of the handler or other individuals can affect the animal’s response, which can be positive or not depending on the interaction. The *ATP6V1B1* gene was also previously associated with carcass composition traits [57], which makes sense due to other biological processes this gene is involved in (e.g., pH regulation, ossification; Supplementary Files S6 and S7). 

The face morphogenesis biological process, annotated for the cysteine-rich secretory protein LCCL domain-containing 1 (*CRISPLD1*) gene, was an interesting result. Neural crest cells are involved in face morphogenesis by generating the craniofacial skeleton, particularly the sensory organs and subsets of cranial sensory receptor neurons, and there are common mechanisms for building faces, brains, peripheral neurons, and central neural circuits that regulate behavioral functions [58]. In addition, the face structure has already been cited as a predictor of aggressiveness in humans, with the specific facial width-to-height ratio highly correlated with aggressiveness in men [59]. However, these associations of face morphogenesis and structure are limited in domestic animals, and the only report for the *CRISPLD1* gene found in cattle was for milk fatty acid traits [60]. 

Previous studies have reported face hair whorls (FHW) in cattle to be a predictor of cattle temperament [61,62,63]. In these studies, animals with FHW above the eye line were more agitated than the ones with lower FHW; these animals escaped faster or displayed aggressive behavior. The association of FHW with cattle temperament may also be related to face morphogenesis, which could be associated with early tissue development, as the same embryonic origin is attributed to the epidermis and the nervous system [64]. Recently, genomic regions for FHW in horses were annotated [65], in which, beyond the hair follicle growth, they were related to neurological and behavioral functions. Despite the phenotypic association of cattle temperament with FHW [61,62,63], no studies underlying the genomic architecture of FHW in cattle were found.

Different dendritic spine densities were previously associated with aggressiveness in rats [66], and the dendrite and dendrite spine morphogenesis GO_BP was annotated for the cell cycle-associated protein 1 (*CAPRIN1*) gene. The *CAPRIN1* gene was previously enriched for bovine respiratory disease [67]; however, studies reporting associations for this gene in cattle are scarce. The haploblock that overlapped the *CAPRIN1* gene (block_135 position 64 Mb on BTA15) also overlapped with 2 QTL for milk fat yield. The fatty acid 2-hydroxylase (*FA2H*) gene was previously associated with carcass traits [68] and was functionally annotated to the central and peripheral nervous system myelin maintenance GO_BP in our study. Differences in the myelinization were found in mice that presented social avoidance behavior (susceptibility to behavioral stress), with less social mice having thinner myelin compared to the controls [69]. Fat is one of the main components of the myelin layers surrounding the nerves and has an important role in the electric transmission across nerve cells [70], and the presence of a fatty acid gene may suggest the involvement of fatty acid metabolism in its formation and maintenance. 

The sperm flagellar 2 (*SPEF2*) gene is a well-known gene in cattle because of its association with important traits, such as adaptation to heat stress [71], fertility [72], and milk production and composition [60]; however, no reports related to behavioral traits for this gene were found. The *SPEF2* gene was found for all scenarios investigated, and beyond the GO_BP related to immune system and fertility, the embryonic neurocranium, brain morphogenesis, and respiratory system development GO_BP were also functionally annotated. Respiration is changed during flight or fight response in animals [73] and consistent respiratory alterations were observed in highly aggressive rats, with elevated basal respiratory rates denoted for the highly aggressive animals compared to controls [74]. Knowledge about alteration of the respiratory rate as a function of behavior is limited in cattle. 

The GO_BP and metabolic pathway that the plexin A1 (*PLXNA1*) gene are involved were mainly related to axon guidance and neuron projection. Specific neuron projections during aggression were previously described in mice, with some periaqueductal gray (PAG) neurons being selective for attack action [75]. No behavior or neuron studies reporting associations of the *PLXNA1* gene were found in cattle; however, this gene was associated to fertility [76] and growth traits [77] in cattle. As the PAG brain region is conserved across species [78] and plays roles in survival behavior [75], further behavioral studies considering this gene in cattle or other species are recommended. 

The social behavior response is affected by the oxytocin signaling pathway (OSP) [79], and this KEGG metabolic pathway was annotated for the calcium voltage-gated channel auxiliary subunit α 2 delta 3 (*CACNA2D3*) gene. Reports related to OSP and social interactions in cattle are scarce. In cattle, the *CACNA2D3* gene was previously associated with intramuscular fat [80], which is in accordance with the fact that the MAPK signaling pathway was also annotated for this gene and is associated with marbling [81], but no studies related to behavior indicators were found.

The vast diversity of functions found by the genes present in the top regions for YT and the previous associations with different sorts of traits may suggest pleiotropic effects. This can also be supported by the range of trait classes presented by QTL overlapped by the top regions (Figure 11), which were related to more than 50 different traits linked to milk production, health, exterior, production, and reproduction traits (Supplementary Files S2 and S3). Three QTL for milking speed (position 43 Mb on BTA7, and positions 30 and 41 Mb on BTA14), an indicator of workability in cattle [82], were overlapped by the top regions when NCSNP and haplotypes were fitted in ssGWAS (Supplementary File S3). Additionally, for another indicator of behavior in cattle, 5 QTL for length of the productive life were found (position 71 Mb on BTA6, positions 21 and 43 Mb on BTA18, position 47 on BTA20, and position 19 on BTA 24). 

### 4.5. Use of Different Linkage Disequilibrium Thresolds and Non-Clustered SNPs in the ssGWAS

Many of the genes and QTL would not have been identified without using different LD thresholds to create the haplotype blocks or the inclusion of the NCSNP. Haplotype-based GWAS using overlapping sliding windows was suggested as more powerful than SNP-based or LD-based haplotype GWAS (considering low recombination rates within haploblocks, i.e., high LD levels), because these would be more efficient for regions with low LD and high recombination [83]. Exclusive genes and QTL were found when different LD thresholds (0.15, 0.50, and 0.80, which are low, moderate, and high, respectively) were used to create the haploblocks, suggesting the LD levels in the regions that affect YT are not consistent. Considering the complexity of the genomic organization, QTL sizes, and genetic factors experienced by the populations, it is unlikely that QTL affecting any trait would follow a specific LD pattern. Using haplotypes and NCSNP under the ssGWAS framework with a low, moderate, and high LD to create the haploblocks allowed us to find genes and biological processes involved in YT that were not reported in previous studies, which used only SNPs, for a set of behavioral traits in cattle. 

The LD-block approach considering high LD levels was previously suggested to be inefficient for association studies because many individual SNPs would be placed out of the haploblocks [84], and thus could not contribute to dissecting the genetic architecture of the trait [35]. Additionally, results from genomic predictions using haplotypes not including the NCSNP were worse regardless of the level of genetic diversity and heritability of the trait, indicating that important genomic regions were not considered without the NCSNP [18]. Our results show important genes and QTL for YT would not be considered without the inclusion of NCSNP, and using both haplotypes and NCSNP accounts for most relevant genes and QTL found based on haplotypes only. Top genomic regions in the X chromosome (PAR region) were found only when NCSNP were included in the analyses. Two genes (*MXRA5* and *CD99* in the position 138 Mb; Supplementary File S3), three QTL related to metabolic body weight (position 136 Mb), and other regions that did not have genes or QTL previously annotated (positions 134 and 135 Mb) were found in the X chromosome (PAR region) when fitting NCSNP and haplotypes together. This finding indicates that further studies including additional markers located in the X chromosome are needed, as also suggested by Alvarenga et al. [5]. Thus, association studies using LD haplotypes should also include the NCSNP. 

It is important to highlight that the density of the panel used to make the haplotype analyses can affect the results, as the amount of QTL variance explained tend to be higher with denser haploblocks [85,86]. The precision in the estimation of the recombination hotspots also tend to be higher with denser SNP panels [87], which can affect the accuracy of haplotype phasing [88]. The 50 K SNP panel used in this research was designed to be similar to the Illumina BovineSNP50V2 and Illumina BovineSNP50V3 SNP panels, with SNPs 50.6 kb apart on average (www.illumina.com/Documents/products/datasheets/datasheet_bovine_snp5O.pdf, accessed on 11 December 2021), presenting SNPs as further apart compared to the high-density panel available for cattle (~777 K SNPs). However, as the haplotype blocks in cattle are ~70 kb [89], the 50 K panel provides reasonable resolution for capturing the extent of LD in the population investigated. Nevertheless, further studies using denser SNP panels are also recommended to investigate ssGWAS using haplotypes for YT, as well as other economically important traits and alternative indicators of cattle temperament.

### 4.6. Future Studies

Additional research should be conducted next to further explore the results obtained in this current study. For instance, it would be valuable to repeat these analyses based on whole-genome sequence data in North American Angus cattle as well as in other worldwide Angus cattle populations. The key candidate genes should also be validated in vitro or based on gene editing and gene knock-out experiments. Furthermore, additional polymorphisms located in the candidate genes identified in this study could be added to existing SNP panels to increase the accuracy of genomic predictions for docility. From a practical point of view, these results obtained could be incorporated in current genomic prediction models for YT in North American Angus. We are also evaluating the genetic trends of docility using the traditional and genomic estimated breeding values for YT and correlated responses in other important traits. Another area of research in our research group is the definition of novel indicators of cattle temperament, especially based on data derived from sensors and other precision technologies.

## 5. Conclusions

Yearling temperament in cattle is a highly polygenic trait, with genes and QTL broadly distributed across the whole genome. Association studies using LD-based haplotypes should include the non-LD-clustered SNPs, as well as different thresholds to increase the likelihood of finding the genomic regions affecting the phenotype of interest. The key candidate genes *ATXN10*, *ADAM10*, *VAX2*, *ATP6V1B1*, *CRISPLD1*, *CAPRIN1*, *FA2H*, *SPEF2*, *PLXNA1*, and *CACNA2D3* are involved in important biological processes and metabolic pathways related to behavioral traits, social interactions, and aggressiveness. Further studies investigating the role of these genes in behavioral traits are recommended. 

## Figures and Tables

**Table 1:** Scenarios used to evaluate the traditional and weighted single-step GWAS (ssGWAS and WssGWAS, respectively) using haplotypes for yearling temperament in American Angus cattle.

Method	Marker Information ^1^	Scenario Abbreviation
ssGWAS	H0.15	ssGWAS_H0.15
	H0.50	ssGWAS_H0.50
	H0.80	ssGWAS_H0.80
	NCSNP_H0.15	ssGWAS_NCSNP_H0.15
	NCSNP_H0.50	ssGWAS_NCSNP_H0.50
	NCSNP_H0.80	ssGWAS_NCSNP_H0.80
WssGWAS iteration 2 (WssGWAS_2)	H0.15	WssGWAS_2_H0.15
	H0.50	WssGWAS_2_H0.50
	H0.80	WssGWAS_2_H0.80
	NCSNP_H0.15	WssGWAS_2_NCSNP_H0.15
	NCSNP_H0.50	WssGWAS_2_NCSNP_H0.50
	NCSNP_H0.80	WssGWAS_2_NCSNP_H0.80
WssGWAS iteration 3 (WssGWAS_3)	H0.15	WssGWAS_3_H0.15
	H0.50	WssGWAS_3_H0.50
	H0.80	WssGWAS_3_H0.80
	NCSNP_H0.15	WssGWAS_3_NCSNP_H0.15
	NCSNP_H0.50	WssGWAS_3_NCSNP_H0.50
	NCSNP_H0.80	WssGWAS_3_NCSNP_H0.80

^1^ H0.15, H0.50, and H0.80: haplotypes from blocks with linkage disequilibrium (LD) thresholds of 0.15, 0.50, and 0.80, respectively; NCSNP_H0.15, NCSNP_H0.50, and NCSNP_H0.80: non-clustered SNP and haplotypes from blocks with LD thresholds of 0.15, 0.50, and 0.80, respectively.

**Table 2:** Descriptive statistics of the haplotype blocks with different linkage disequilibrium (LD) thresholds used in each scenario, before and after quality control (QC), in American Angus cattle.

Descriptive	LD_0.15	LD_0.50	LD_0.80
Number of non-clustered SNPs	20,849	30,501	36,811
Number of clustered SNPs	21,784	12,132	5822
Number of blocks	9634	5617	2721
Minimum number of SNP in blocks	2	2	2
Maximum number of SNP in blocks	9	7	7
Average (SD ^1^) block size (Mb)	0.035 (0.020)	0.032 (0.014)	0.030 (0.013)
Minimum block size (bp)	65	65	84
Maximum block size (Mb)	0.201	0.161	0.160
Number of pseudo-SNPs ^2^ before QC	56,734	27,324	12,877
Number of pseudo-SNPs after QC	44,559	23,918	11,389
Number of non-clustered and pseudo-SNPs before QC	77,583	57,825	49,688
Number of non-clustered and pseudo-SNPs after QC	65,435	54,444	48,227

^1^ Standard deviation. ^2^ Pseudo-SNPs are the unique haplotype alleles from the combination of phased SNPs within haplotype blocks.

**Table 3:** Gene ontology biological terms (GO_BP) and metabolic pathways from the Kyoto Encyclopedia of Genes and Genomes (KEGG) access of genes overlapped by top 0.001% markers for docility in American Angus cattle ^1^.

Chromosome	Gene	S_P (Mb) ^2^	E_P (Mb) ^3^	GO_BP	KEGG
BTA5	*ATXN10*	116.029	116.169	GO:0031175	-
BTA10	*ADAM10*	51.536	51.679	-	bta05010
BTA11	*VAX2*	13.483	13.509	GO:0007409, GO:0007601, GO:0030900, GO:0048048, GO:0060041	-
BTA11	*ATP6V1B1*	13.454	13.480	GO:00076605, GO:0042472	-
BTA14	*CRISPLD1*	38.295	38.346	GO:0060325	-
BTA15	*CAPRIN1*	64.662	64.697	GO:0050775, GO0061003	
BTA18	*FA2H*	2.151	2.206	GO:0032286, GO:0032287	-
BTA20	*SPEF2*	38.369	38.573	GO:0048702, GO:0048854, GO:0069541	-
BTA22	*PLXNA1*	60.240	60.280	GO:0021785, GO: 0048841, GO:1902287, GO:1990138	bta04360
BTA22	*CACNA2D3*	45.925	46.819	-	bta04921

^1^ Only genes with a GO_BP or metabolic pathway related to a behavior or docility trait are presented. Details for all genes harboring the top markers are presented in Supplementary Files S6 and S7. ^2^ Start position. ^3^ End position.

## Data Availability

The phenotypic and genomic data used in this study are the property of the industry partner that contributed to the study and therefore are not readily available due to its commercial sensitivity. Requests to access the datasets should be directed to the American Angus Association. The computing pipelines used in this research are available by request to the corresponding authors.

## Supplementary Materials

The following supporting information can be downloaded at: https://www.mdpi.com/article/10.3390/genes13010017/s1, File S1: Distributions of the percentage of the additive variance explained by makers from single-step GWAS using haplotypes for yearling temperament in American Angus cattle. File S2: Genes and QTL overlapped with top regions using haplotypes in single-step GWAS for yearling temperament in American Angus cattle. File S3: Genes and QTL overlapped with top regions using haplotypes and non-clustered SNPs in single-step GWAS for yearling temperament in American Angus cattle. File S4: Overlapped top regions among scenarios using haplotypes or non-clustered SNPs in single-step GWAS for yearling temperament in American Angus cattle. File S5: Overlapped top genes among scenarios using haplotypes or non-clustered SNPs in single-step GWAS for yearling temperament in American Angus cattle. File S6: Functional annotation tables for genes overlapped by top regions using haplotypes in single-step GWAS for yearling temperament in American Angus cattle. File S7: Functional annotation tables for genes overlapped by top regions using haplotypes and non-clustered SNPs in single-step GWAS for yearling temperament in American Angus cattle. File S8: Overlapped QTL among scenarios using haplotypes or non-clustered SNPs in single-step GWAS for yearling temperament in American Angus cattle. File S9: Correlations between the GEBV used in the ssGWAS and WssGWAS with haplotypes and non-clustered SNPs for yearling temperament in American Angus.
