# Supplementary material for: Haplotype-Based Single-Step GWAS for Yearling Temperament in American Angus Cattle
Source: Genes (Basel). 2021 Dec 22;13(1):17. doi: 10.3390/genes13010017 (PMC8775055; doi:10.3390/genes13010017)
Supplement: Supplementary file 1 [file genes-13-00017-s001.zip › genes-1492001-supplementary/Supplementary_File_S1.pdf]

genes MDPI

Statistics: percentage variance explained by haplotypes from blocks with LD_0.15
WssGWAS iteration 1 (ssGWAS)

summary statistics
------
min: 0   max: 0.14619
median: 0.00696
mean: 0.01038186
estimated sd: 0.01104523
estimated skewness: 2.434889
estimated kurtosis: 13.02973

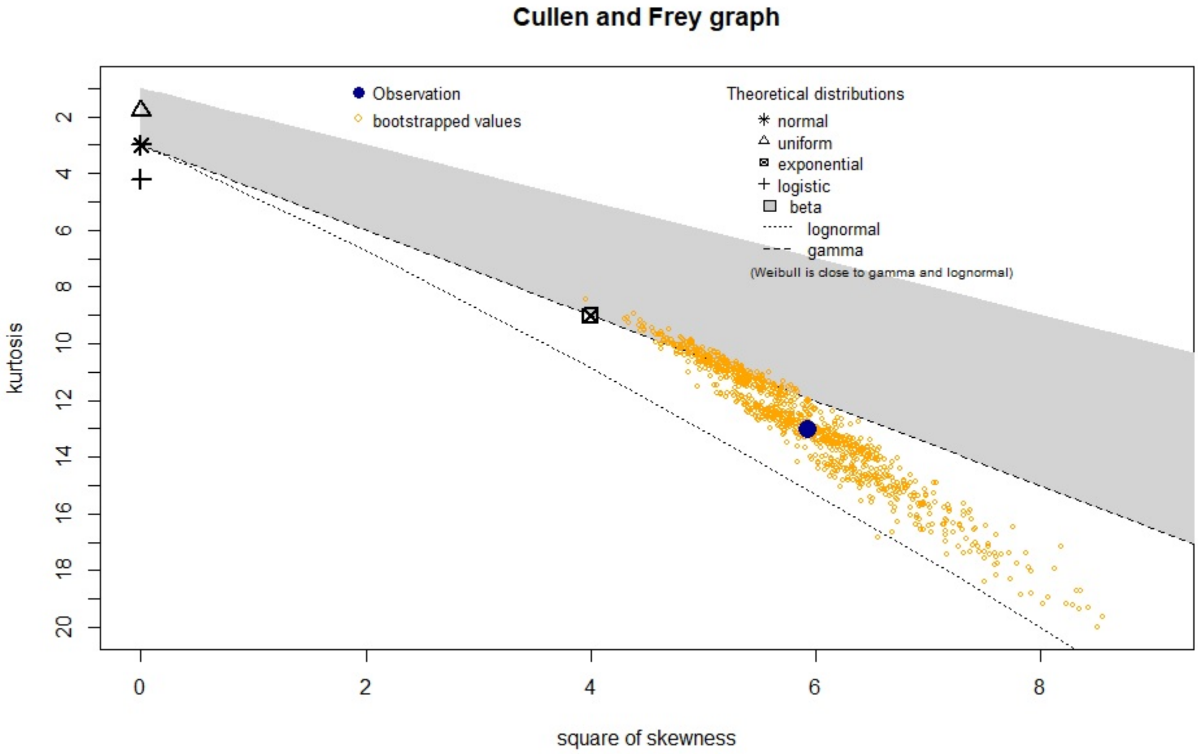

Fitting of the distribution ' beta ' by matching moments
Parameters :
estimate
shape1 0.8640248
shape2 82.3604749

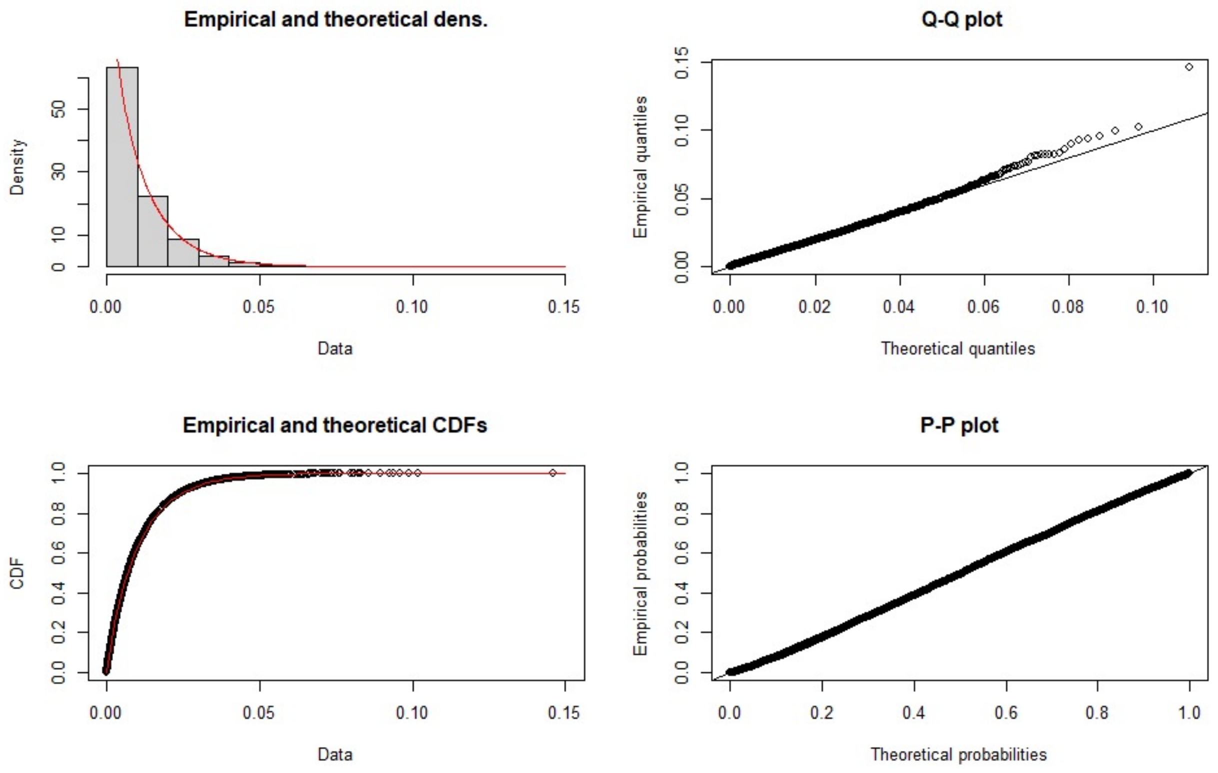

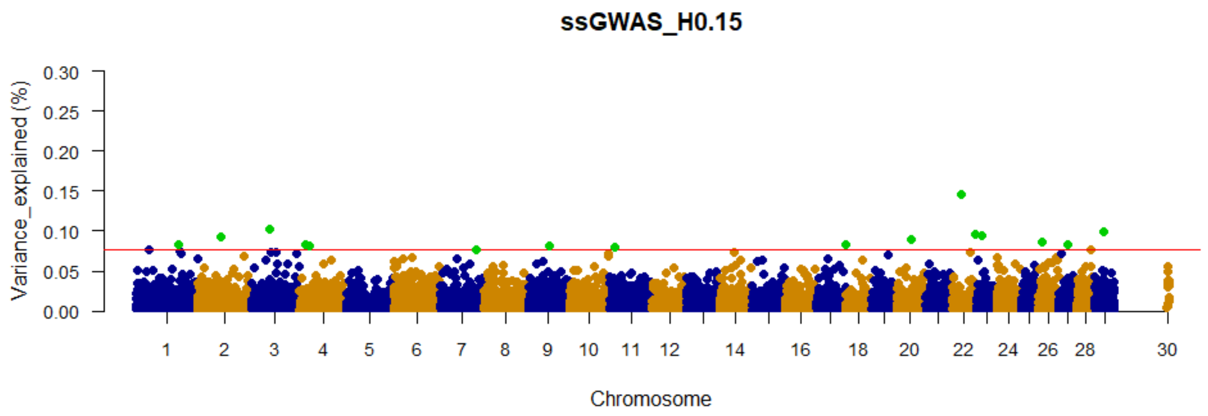

Statistics: percentage variance explained by haplotypes from blocks with LD_0.15
WssGWAS iteration 2 (WssGWAS)

summary statistics
------
min: 0   max: 0.11174
median: 0.00722
mean: 0.01038182
estimated sd: 0.0103068
estimated skewness: 2.014777
estimated kurtosis: 9.237767

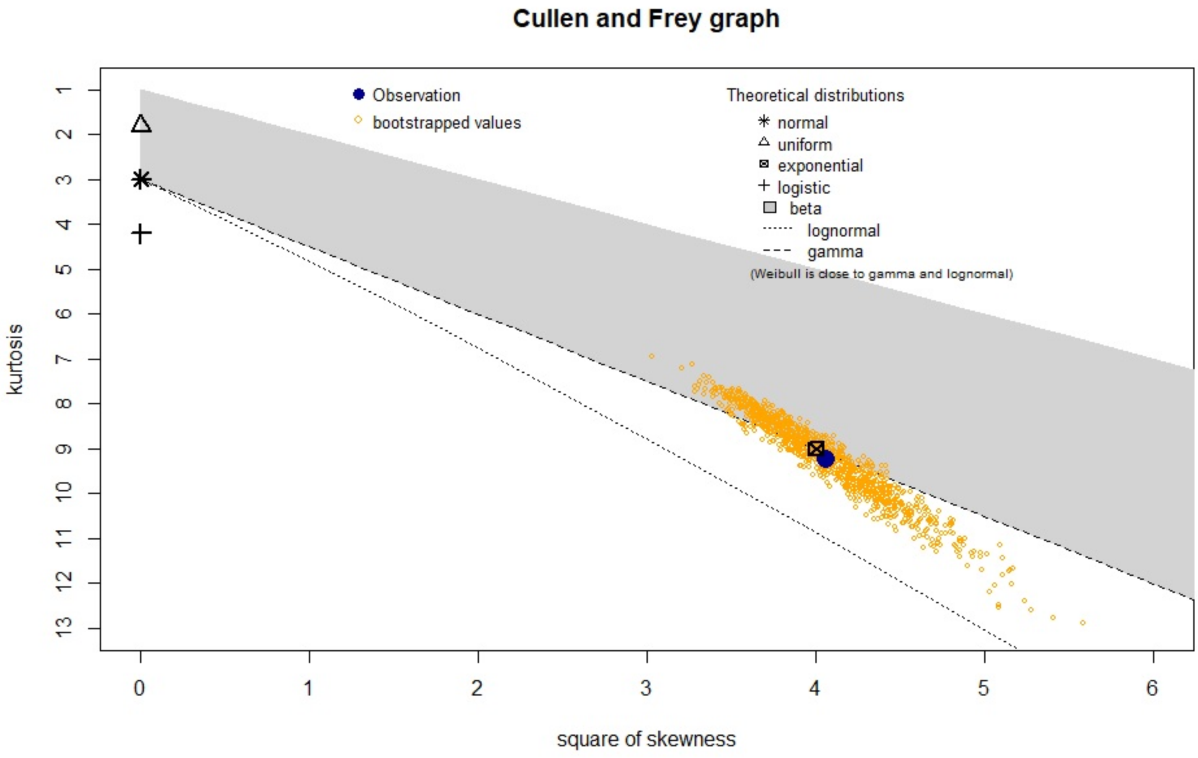

Fitting of the distribution ' beta ' by matching moments
Parameters :
estimate
shape1 0.9938006
shape2 94.7312646

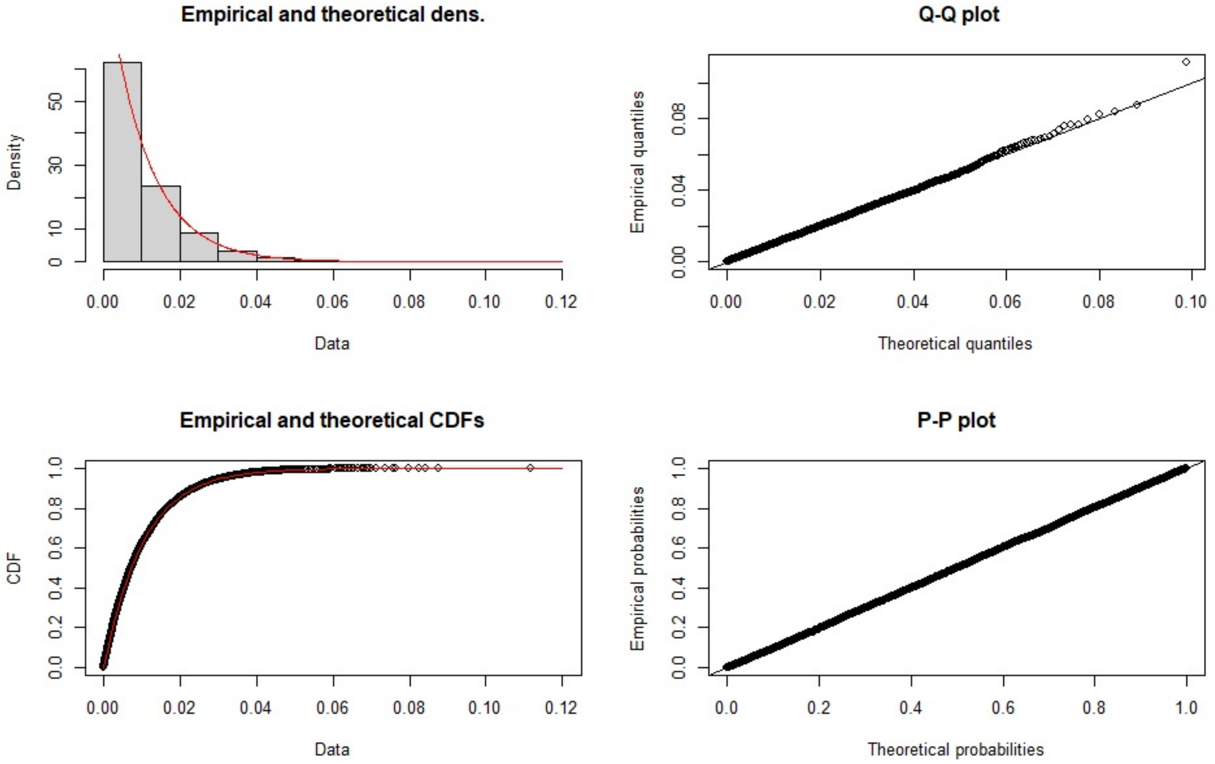

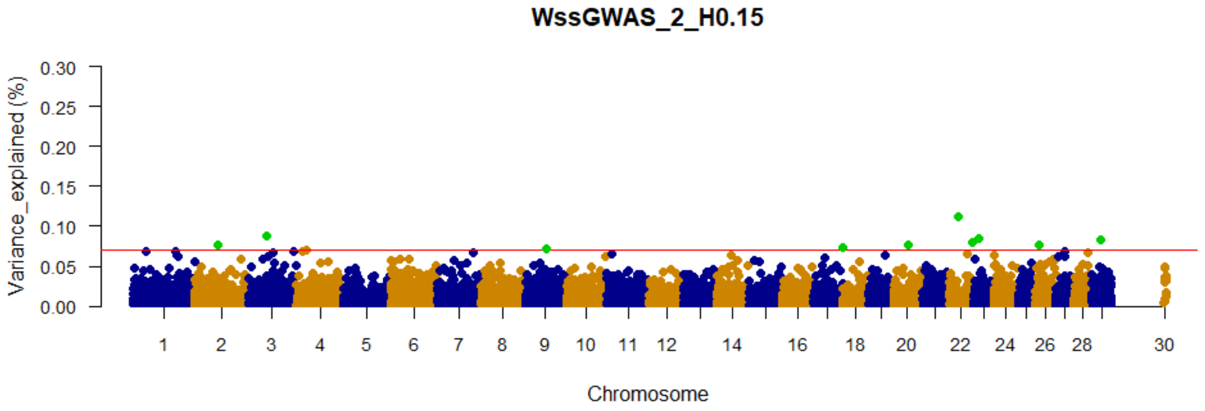

Statistics: percentage variance explained by haplotypes from blocks with LD_0.15
WssGWAS iteration 3 (WssGWAS)

summary statistics
------
min: 0   max: 0.11876
median: 0.0072
mean: 0.01038177
estimated sd: 0.01039503
estimated skewness: 2.085039
estimated kurtosis: 9.878912

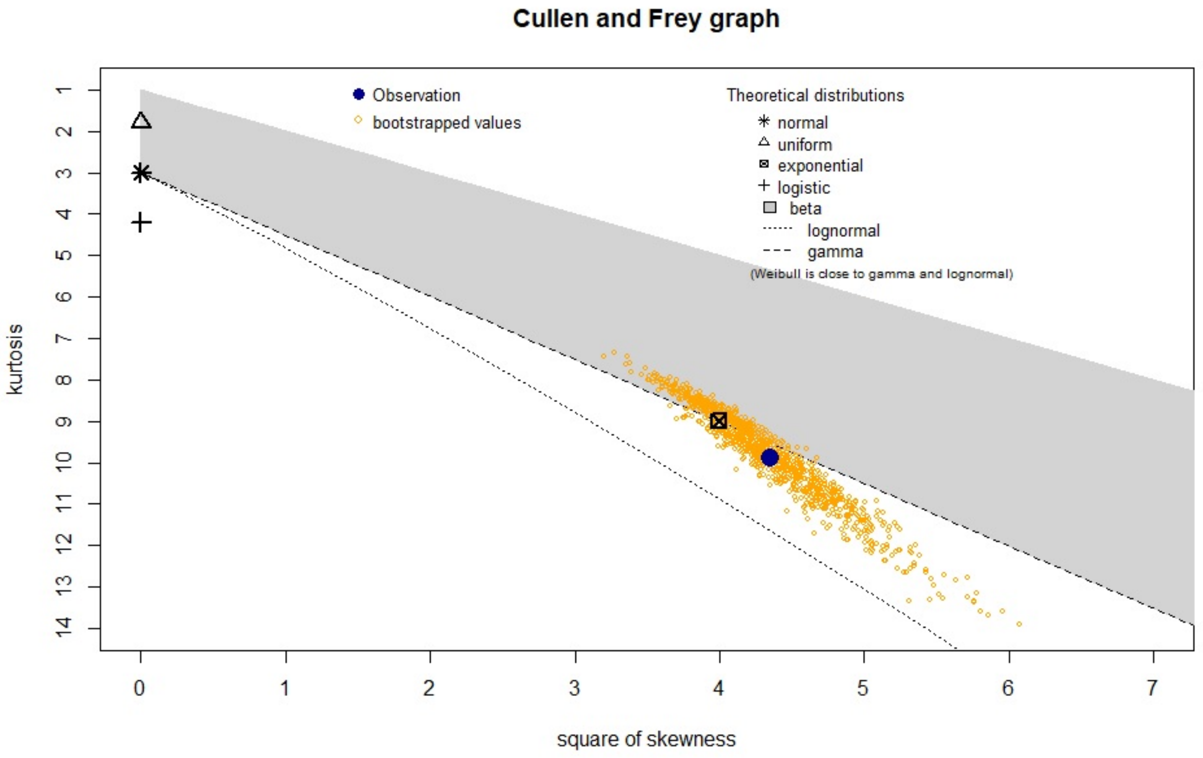

Fitting of the distribution ' gamma ' by matching moments
Parameters :
        estimate
shape 0.9975547
rate 96.0871396

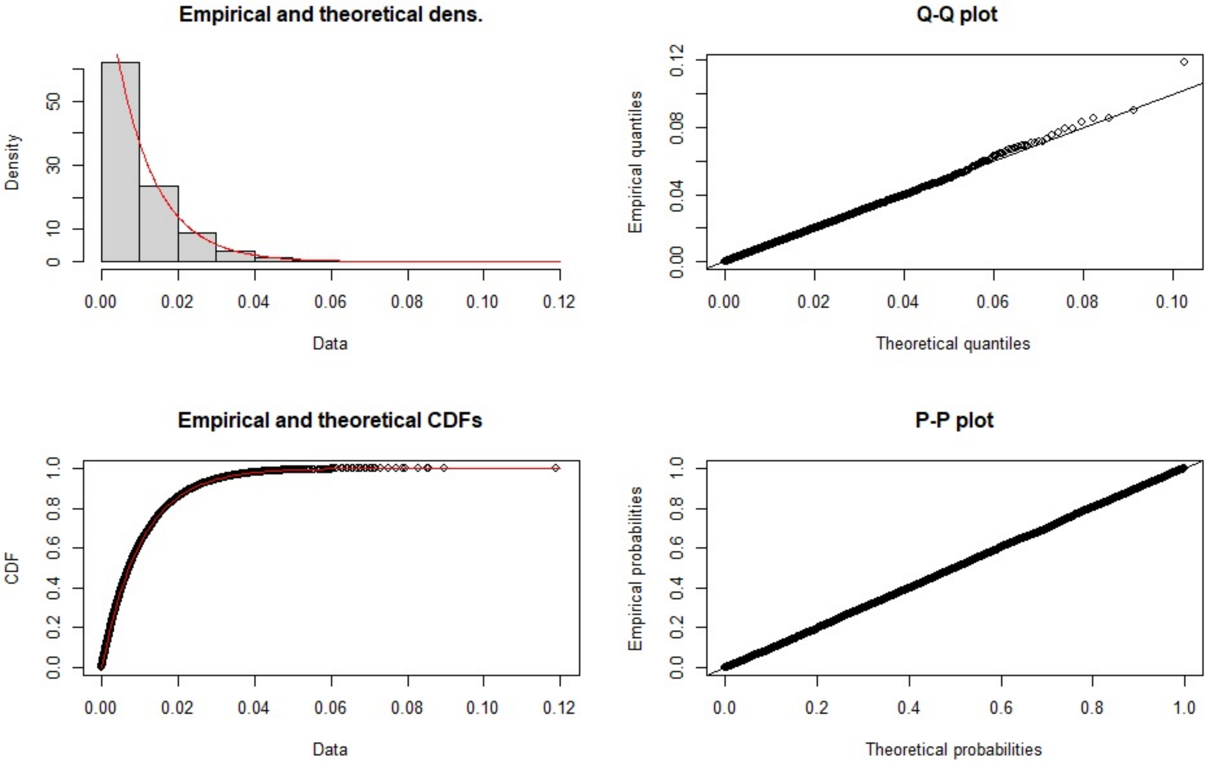

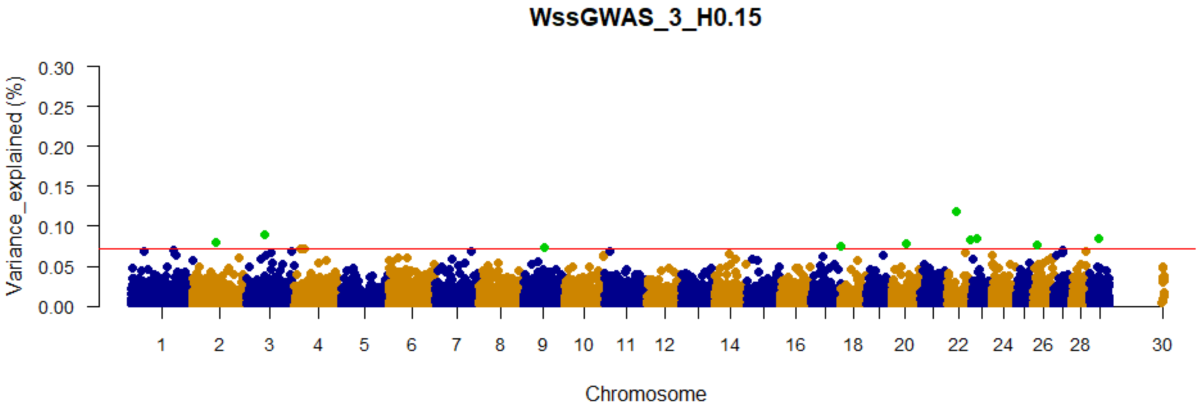

Statistics: percentage variance explained by haplotypes from blocks with LD_0.50
WssGWAS iteration 1 (ssGWAS)

summary statistics
------
min: 0 max: 0.1623
median: 0.01234
mean: 0.01780299
estimated sd: 0.01780225
estimated skewness: 2.127498
estimated kurtosis: 9.980306

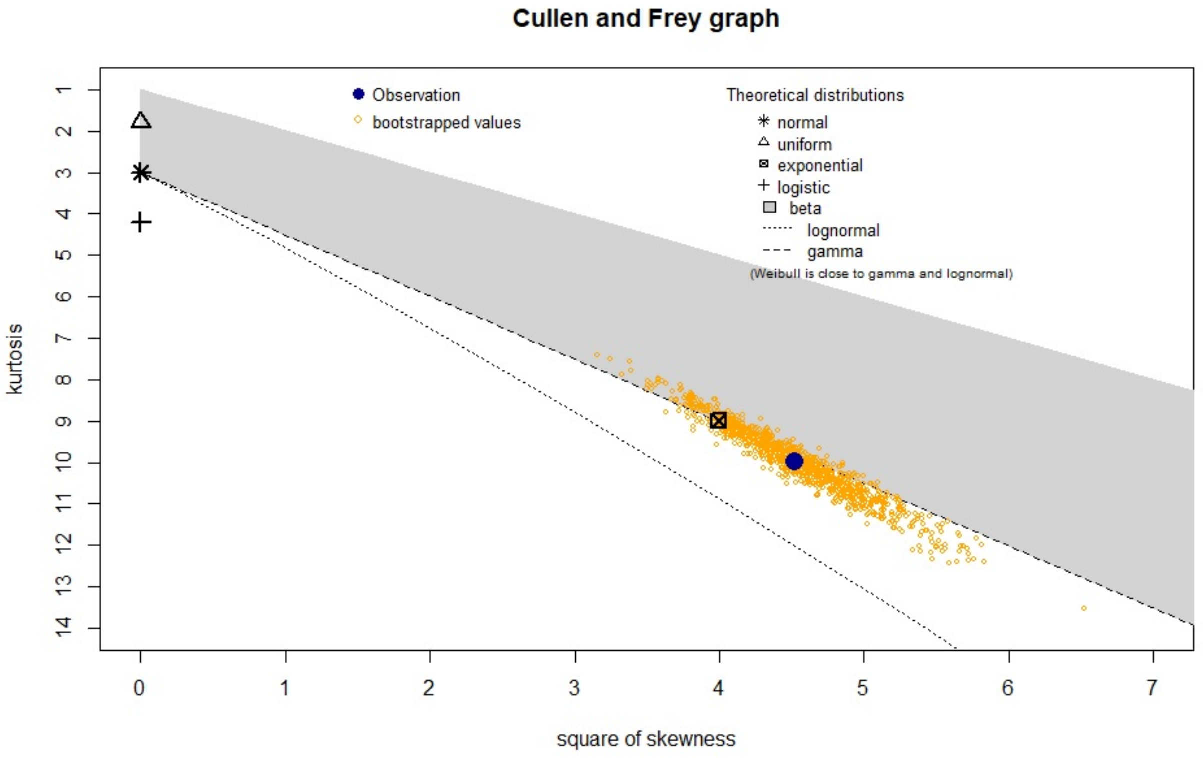

Fitting of the distribution ' beta ' by matching moments
Parameters :
estimate
shape1 0.9646514
shape2 53.2201335

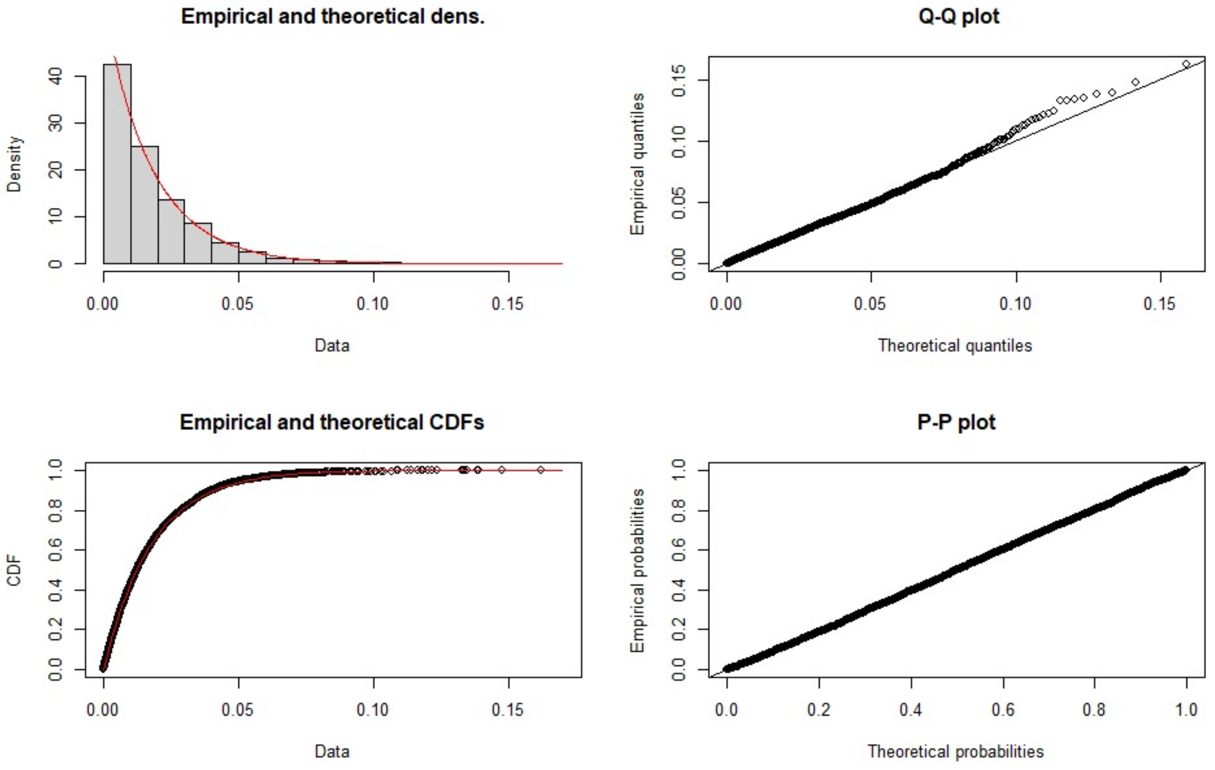

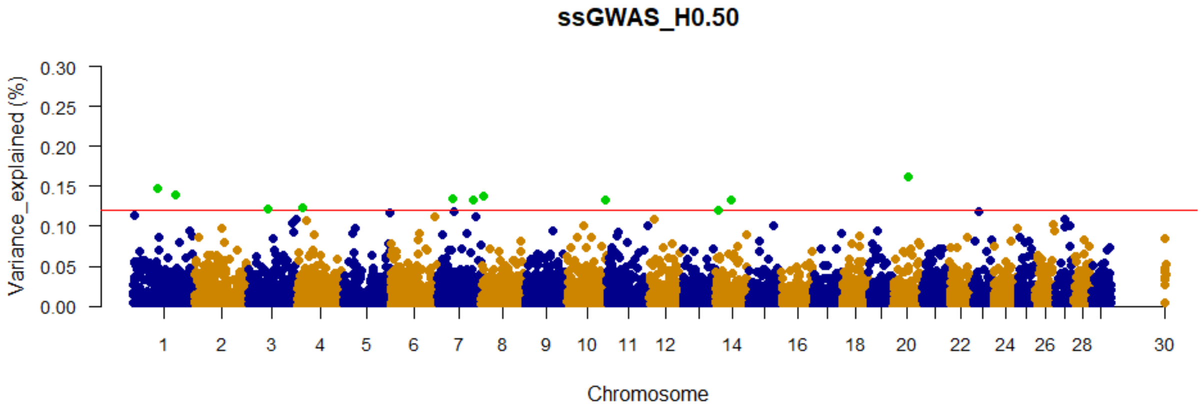

Statistics: percentage variance explained by haplotypes from blocks with LD_0.50
WssGWAS iteration 2 (WssGWAS)

summary statistics
------
min: 0 max: 0.13383
median: 0.01303
mean: 0.01780287
estimated sd: 0.01635826
estimated skewness: 1.69526
estimated kurtosis: 7.043574

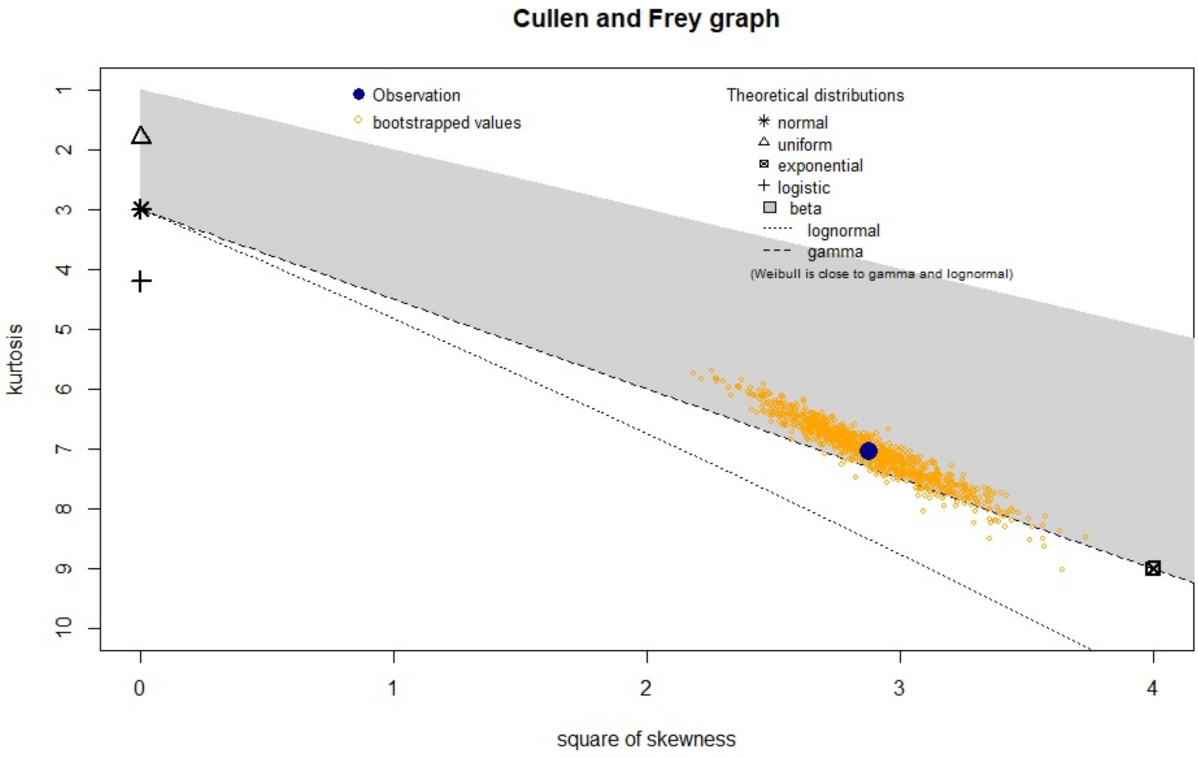

Fitting of the distribution ' beta ' by matching moments
Parameters :
estimate
shape1 1.145738
shape2 63.211191

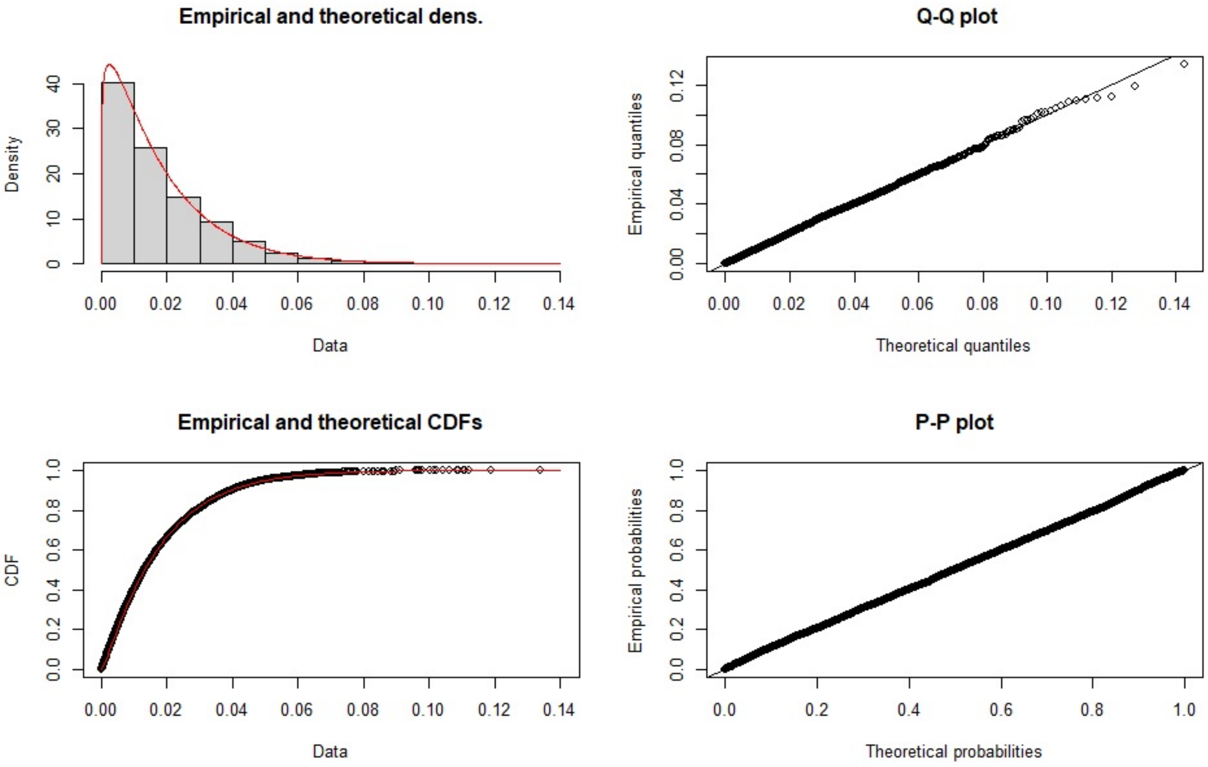

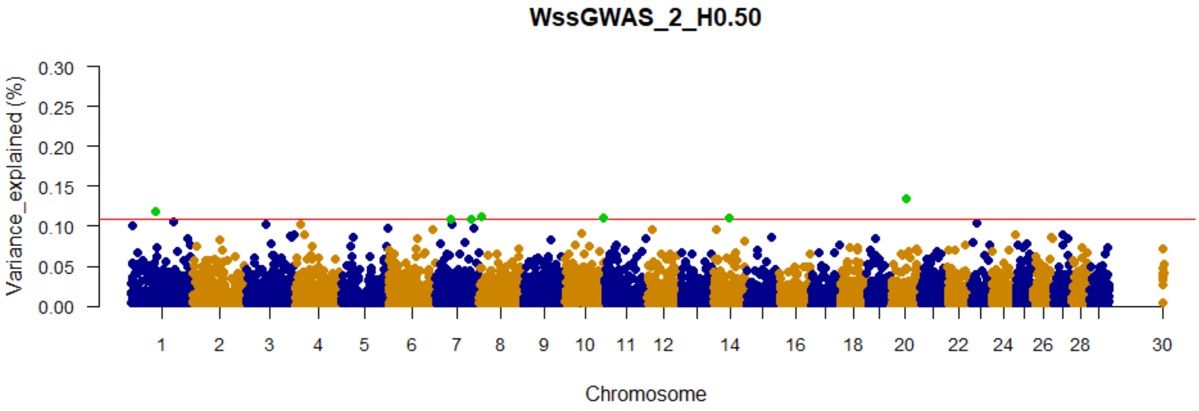

Statistics: percentage variance explained by haplotypes from blocks with LD_0.50
WssGWAS iteration 3 (WssGWAS)

summary statistics
------
min: 0 max: 0.13975
median: 0.01295
mean: 0.01780288
estimated sd: 0.01654919
estimated skewness: 1.775167
estimated kurtosis: 7.591097

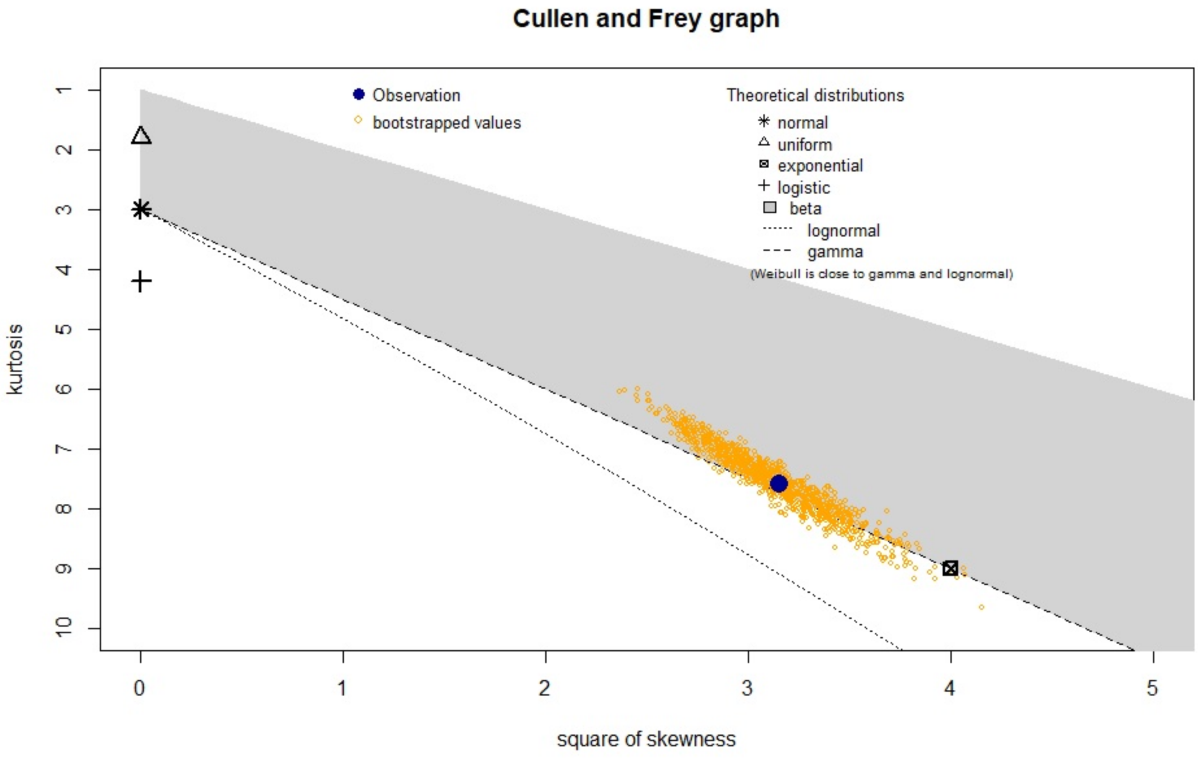

Fitting of the distribution ' beta ' by matching moments
Parameters :
estimate
shape1 1.119046
shape2 61.738552

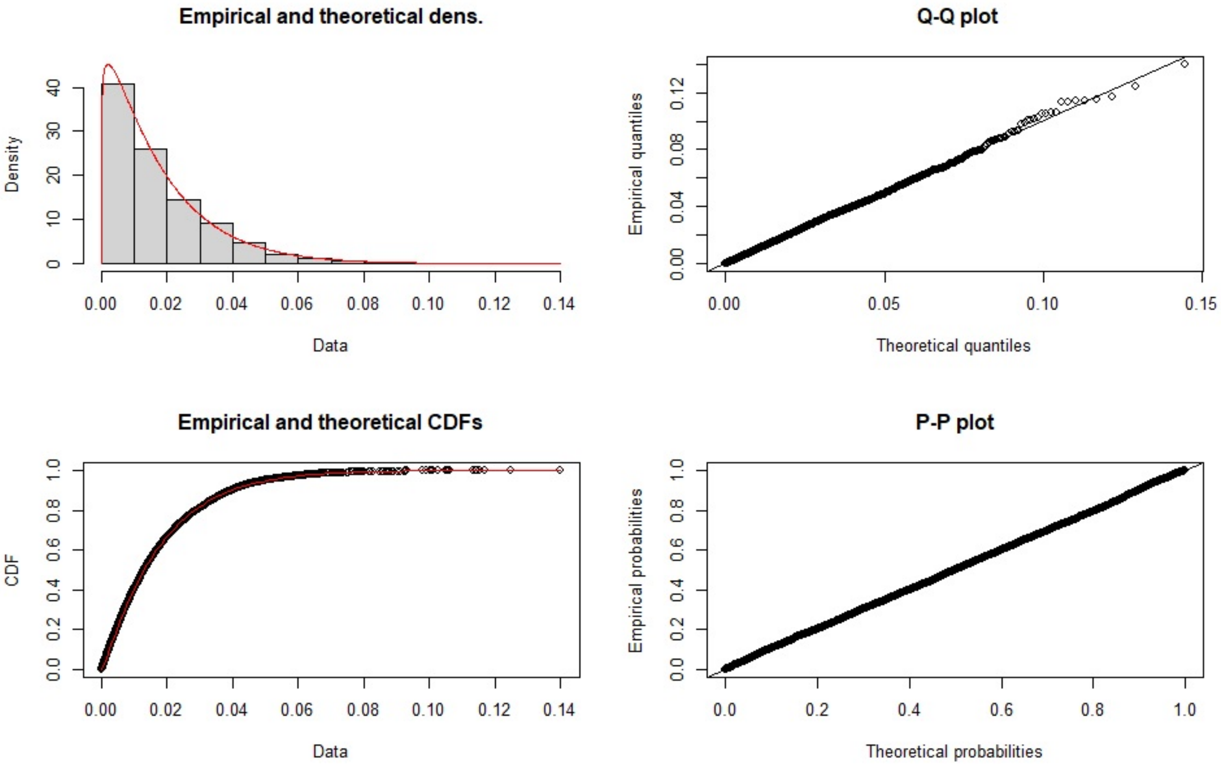

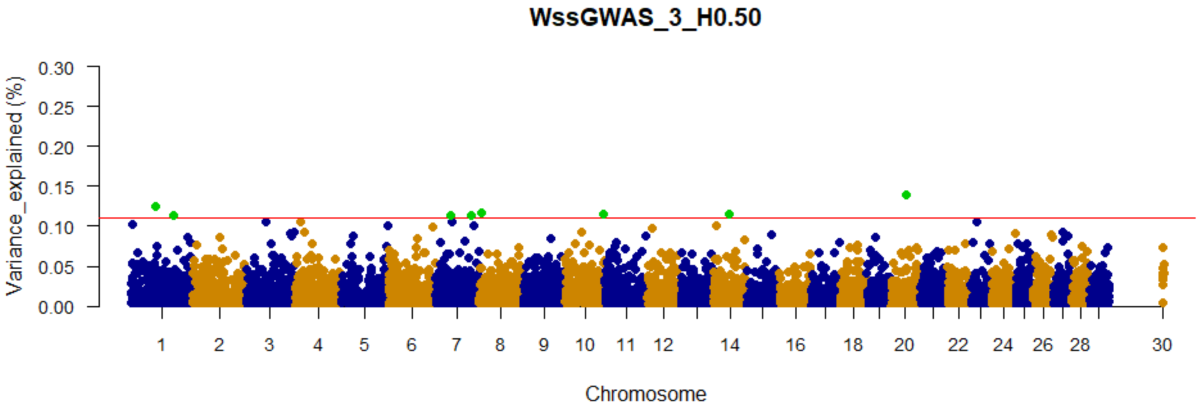

Statistics: percentage variance explained by haplotypes from blocks with LD_0.80
WssGWAS iteration 1 (ssGWAS)

summary statistics
------
min: 0 max: 0.27178
median: 0.02626
mean: 0.03675104
estimated sd: 0.0349454
estimated skewness: 1.951988

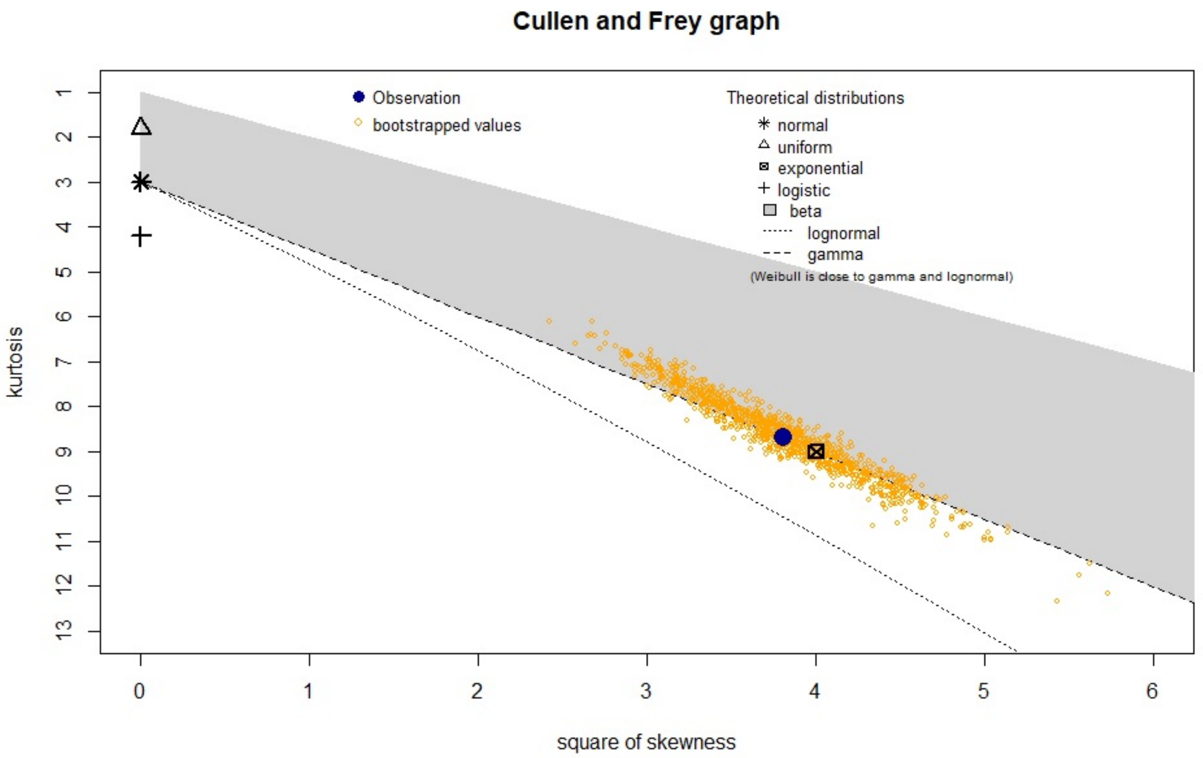

Fitting of the distribution ' beta ' by matching moments
Parameters :

	estimate
shape1	1.029004
shape2	26.970321

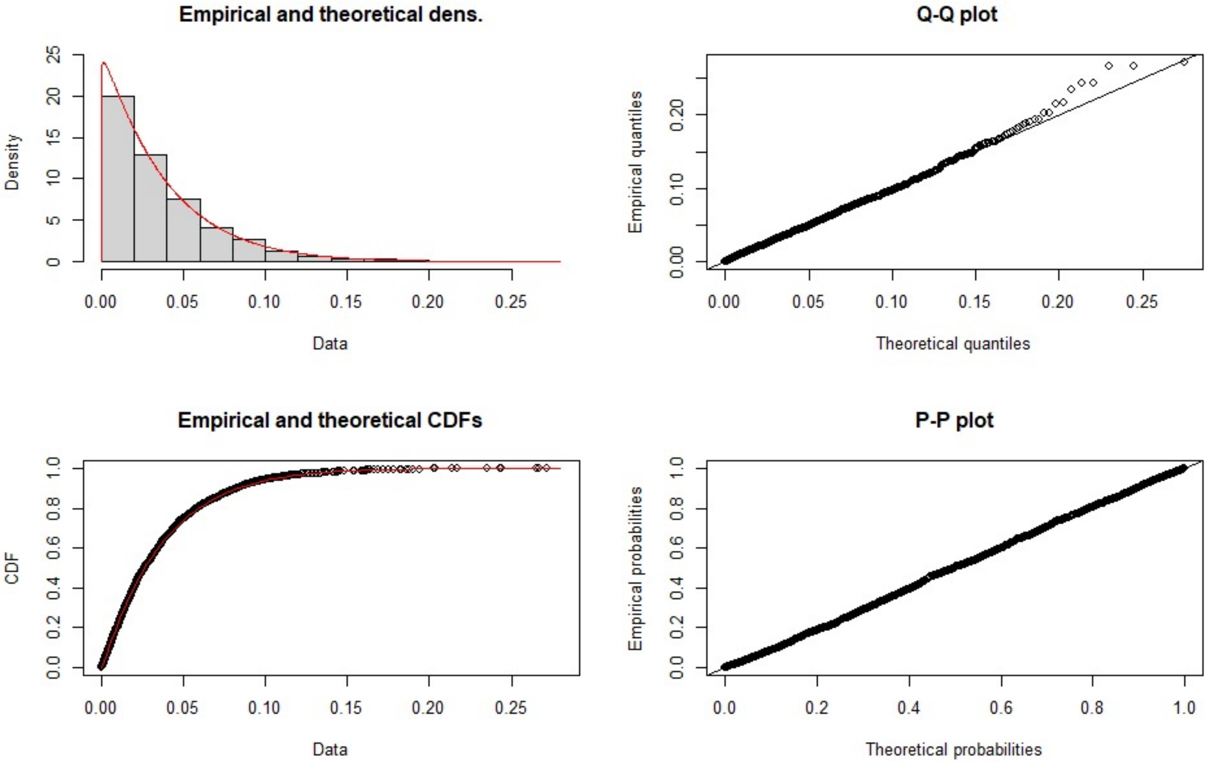

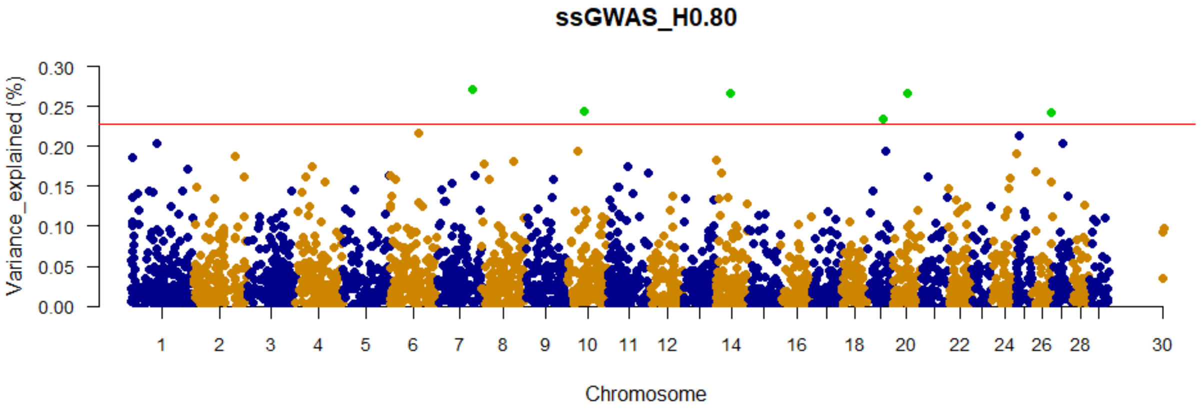

Statistics: percentage variance explained by haplotypes from blocks with LD_0.80 WssGWAS iteration 2 (WssGWAS)

summary statistics
------
min: 0 max: 0.21691
median: 0.02794
mean: 0.03675115
estimated sd: 0.03164835
estimated skewness: 1.533357
estimated kurtosis: 6.171605

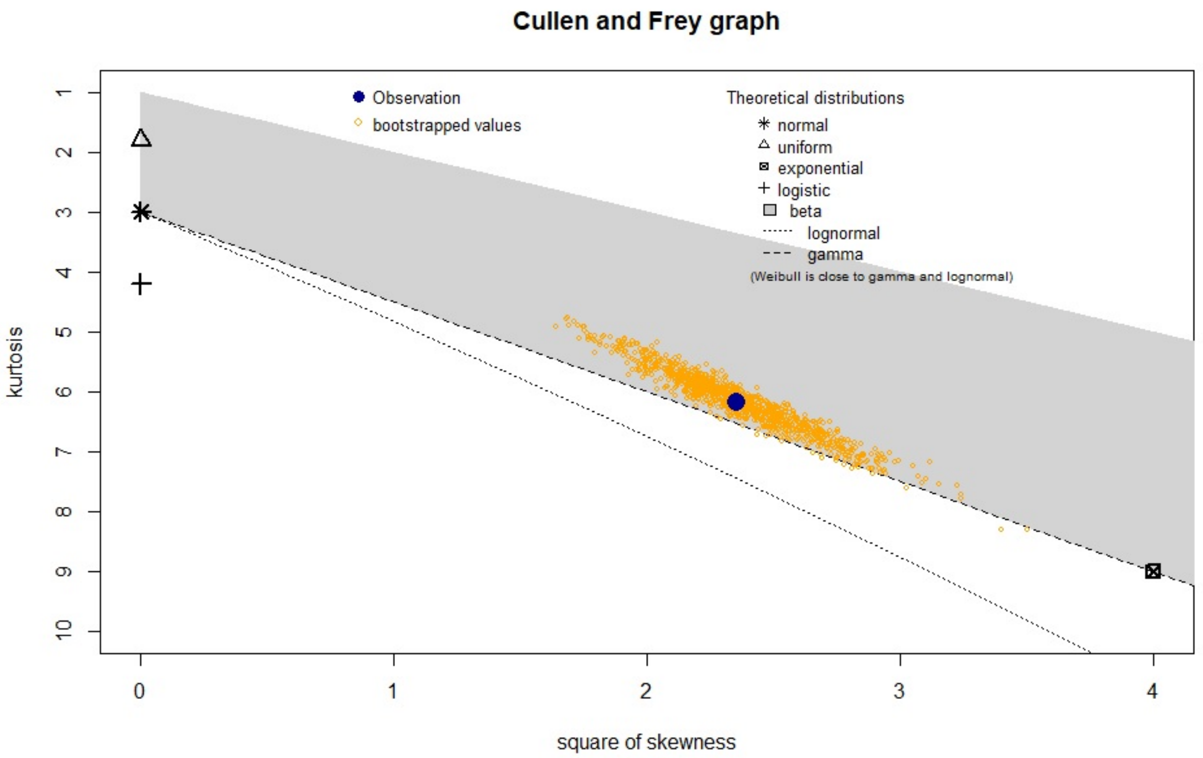

Fitting of the distribution ' beta ' by matching moments
Parameters :

	estimate
shape1	1.262633
shape2	33.093668

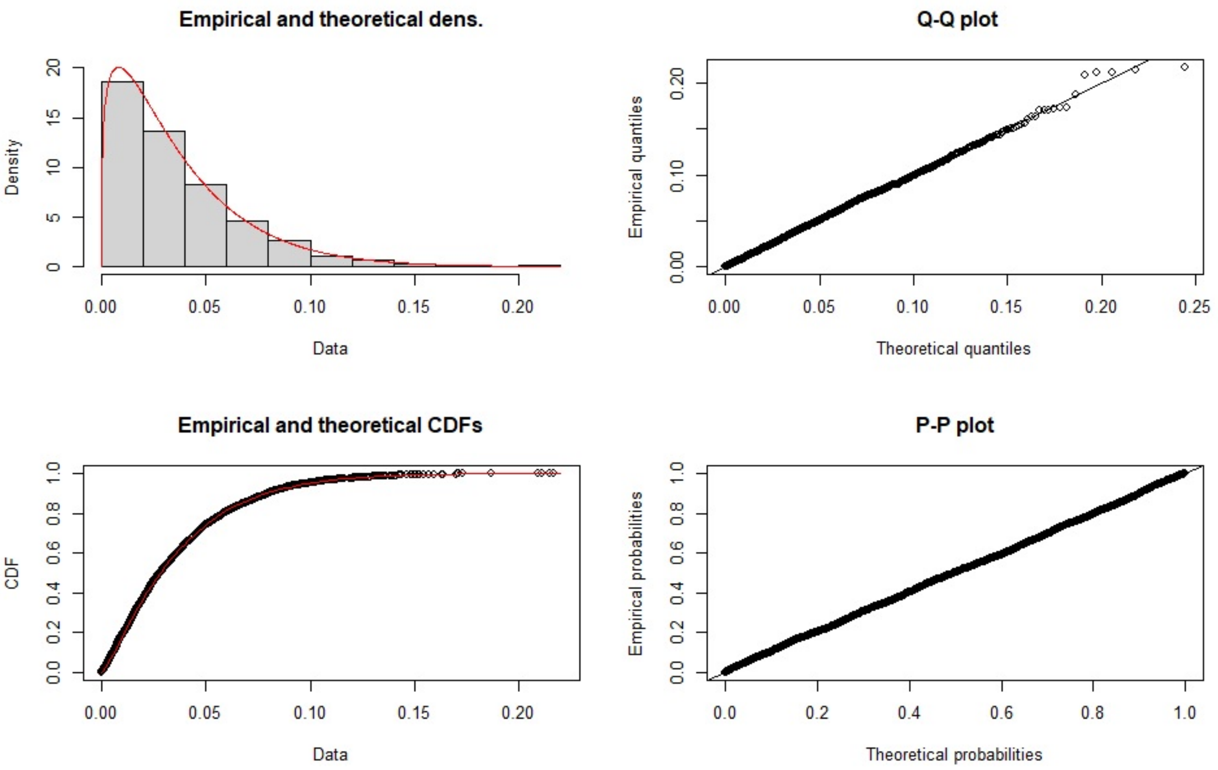

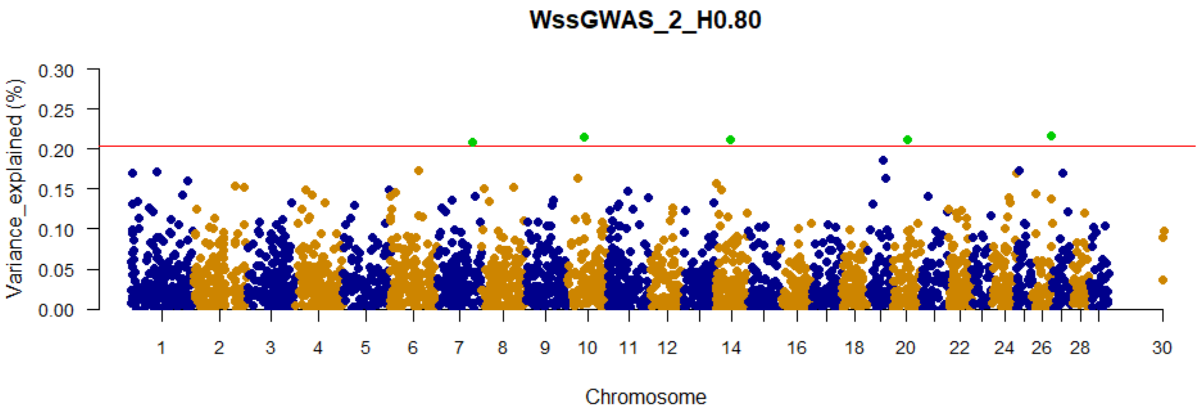

Statistics: percentage variance explained by haplotypes from blocks with LD_0.80
WssGWAS iteration 3 (WssGWAS)

summary statistics
------
min: 0 max: 0.22334
median: 0.02774
mean: 0.03675105
estimated sd: 0.03212226
estimated skewness: 1.619313
estimated kurtosis: 6.679214

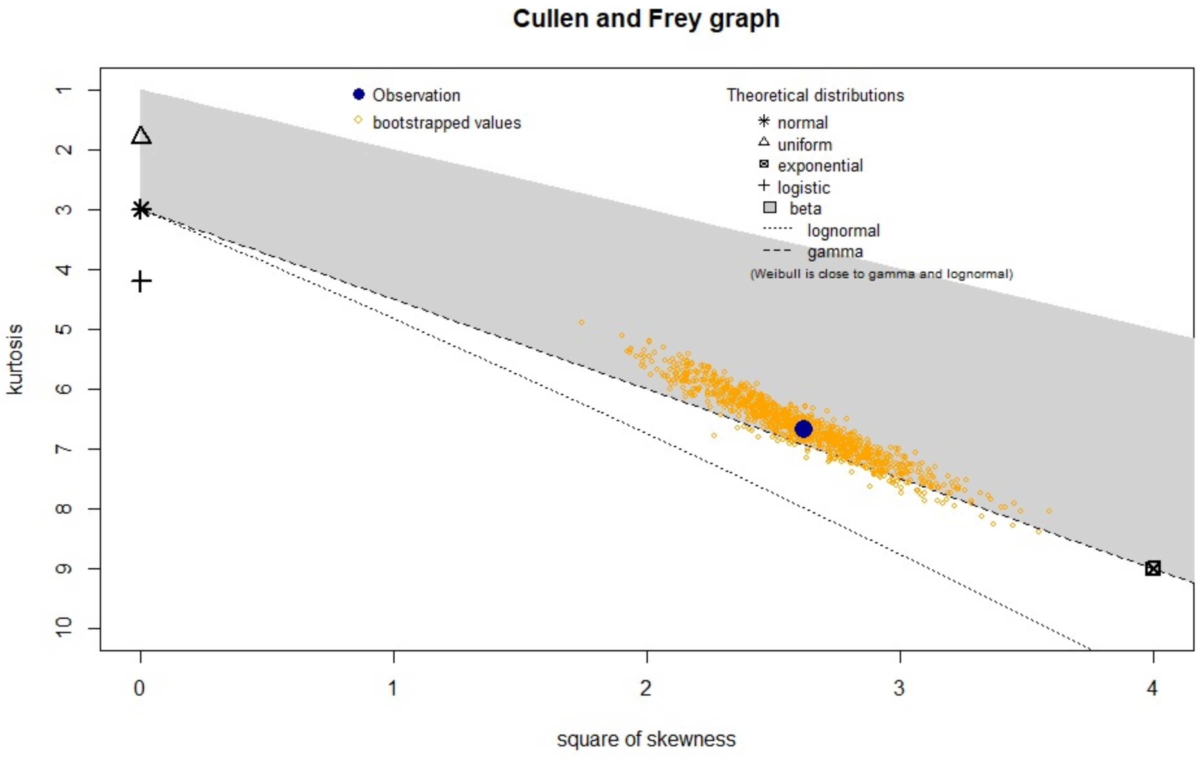

Fitting of the distribution ' beta ' by matching moments
Parameters :
estimate
shape1 1.224569
shape2 32.096091

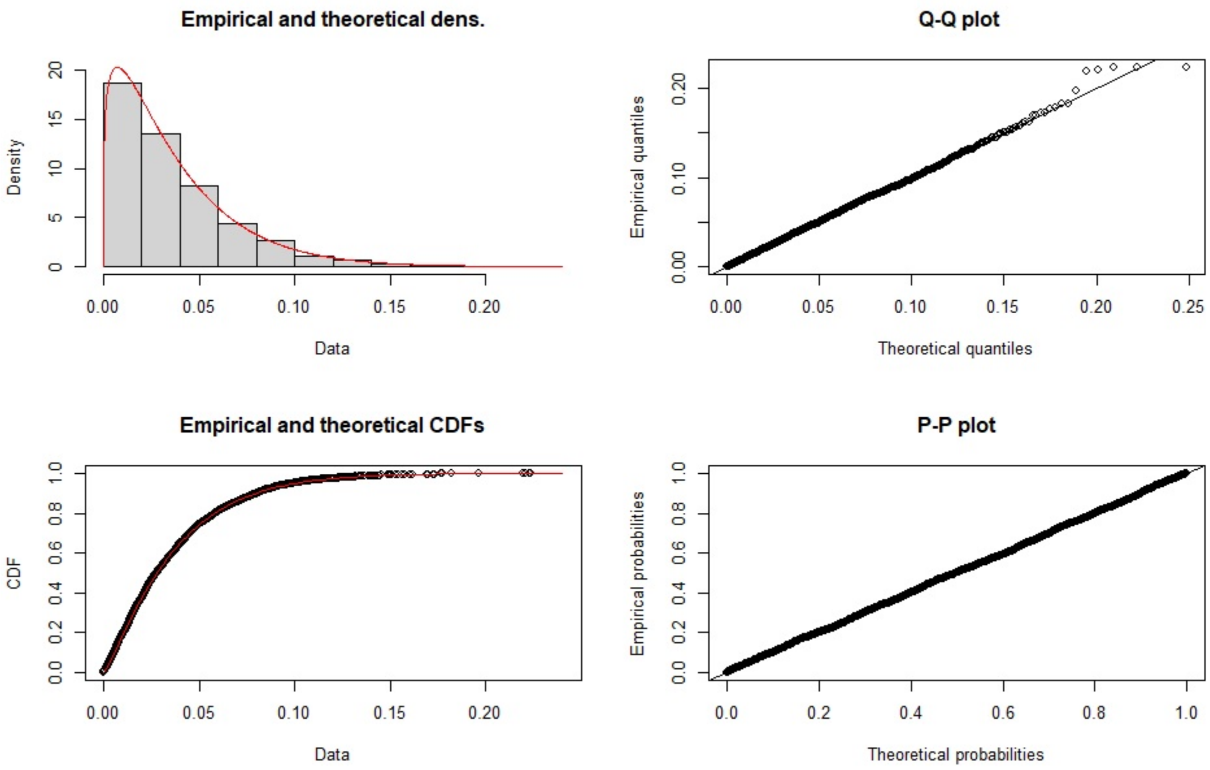

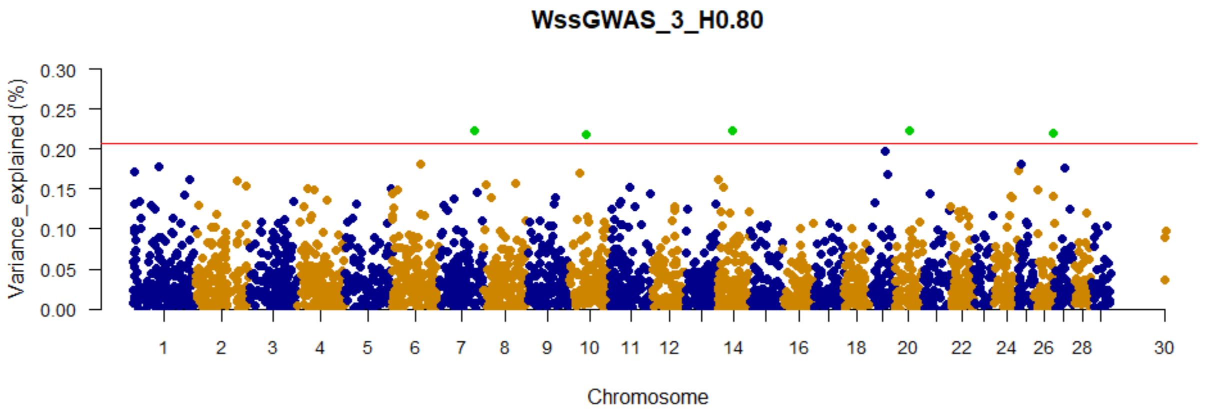

Statistics: percentage variance explained by haplotypes and non-clustered SNPs from blocks with LD_0.15

WssGWAS iteration 1 (ssGWAS)

summary statistics
------
min: 0   max: 0.08996
median: 0.00102
mean: 0.003277807
estimated sd: 0.005750574
estimated skewness: 3.888373
estimated kurtosis: 26.44834

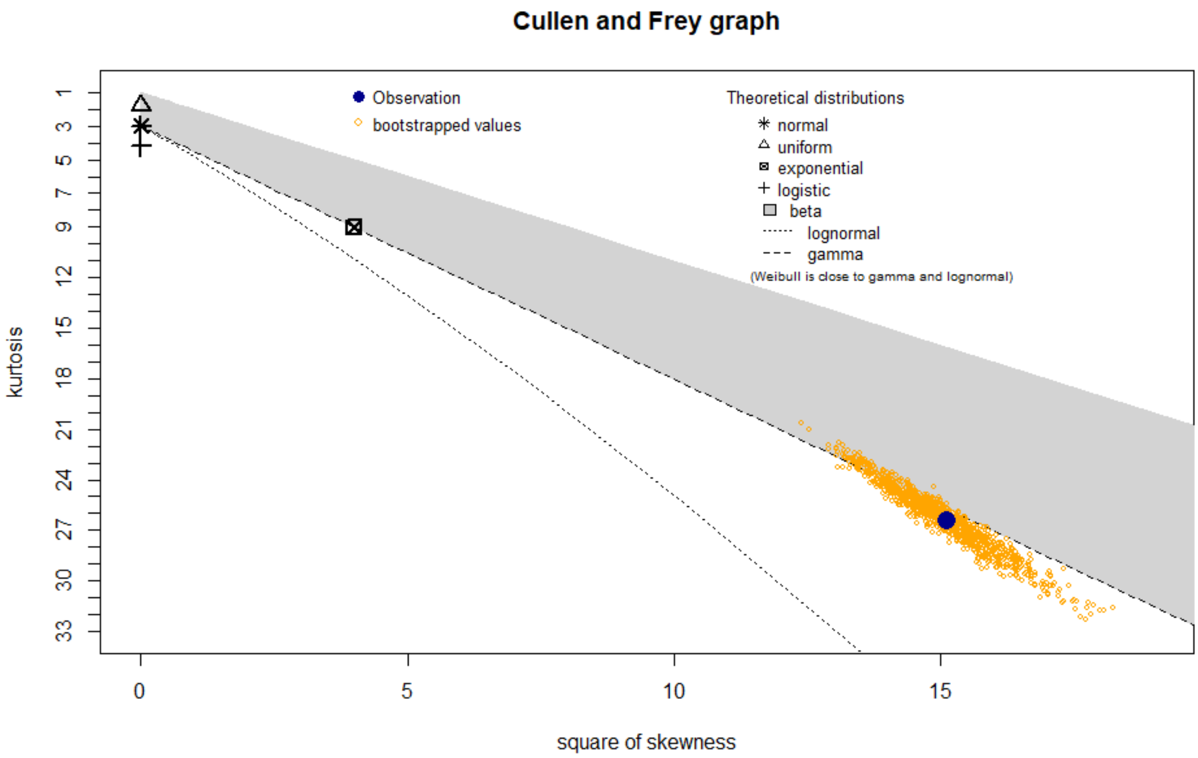

Fitting of the distribution ' gamma ' by matching moments
Parameters :
        estimate
shape 0.3249067
rate 99.1231862

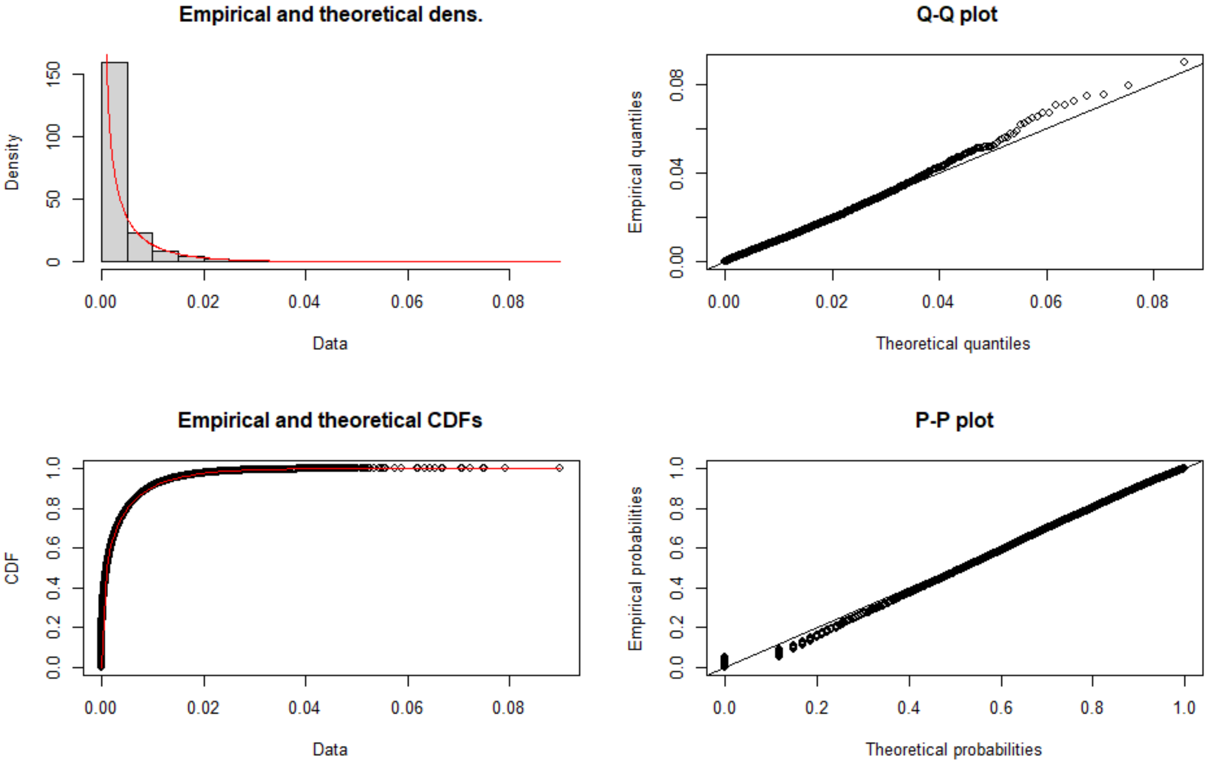

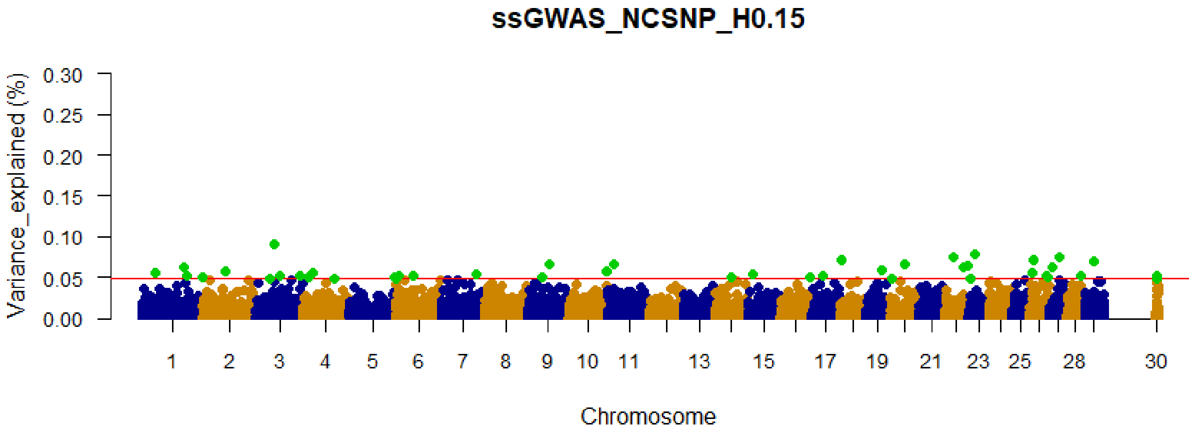

Statistics: percentage variance explained by haplotypes and non-clustered SNPs from blocks with LD_0.15

WssGWAS iteration 2 (WssGWAS)

summary statistics
------
min: 0   max: 0.07945
median: 0.00105
mean: 0.003277817
estimated sd: 0.00553031
estimated skewness: 3.507454
estimated kurtosis: 21.40061

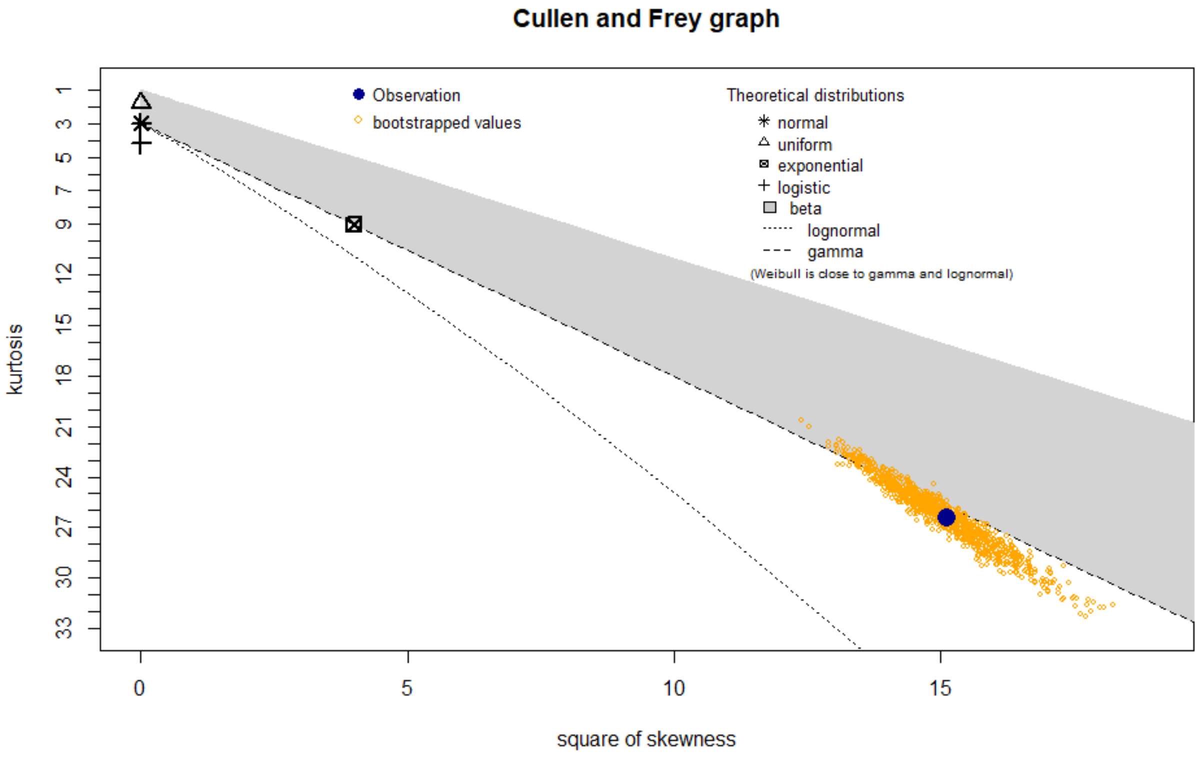

Fitting of the distribution ' gamma ' by matching moments
Parameters :

	estimate
shape	0.3513052
rate	107.176587

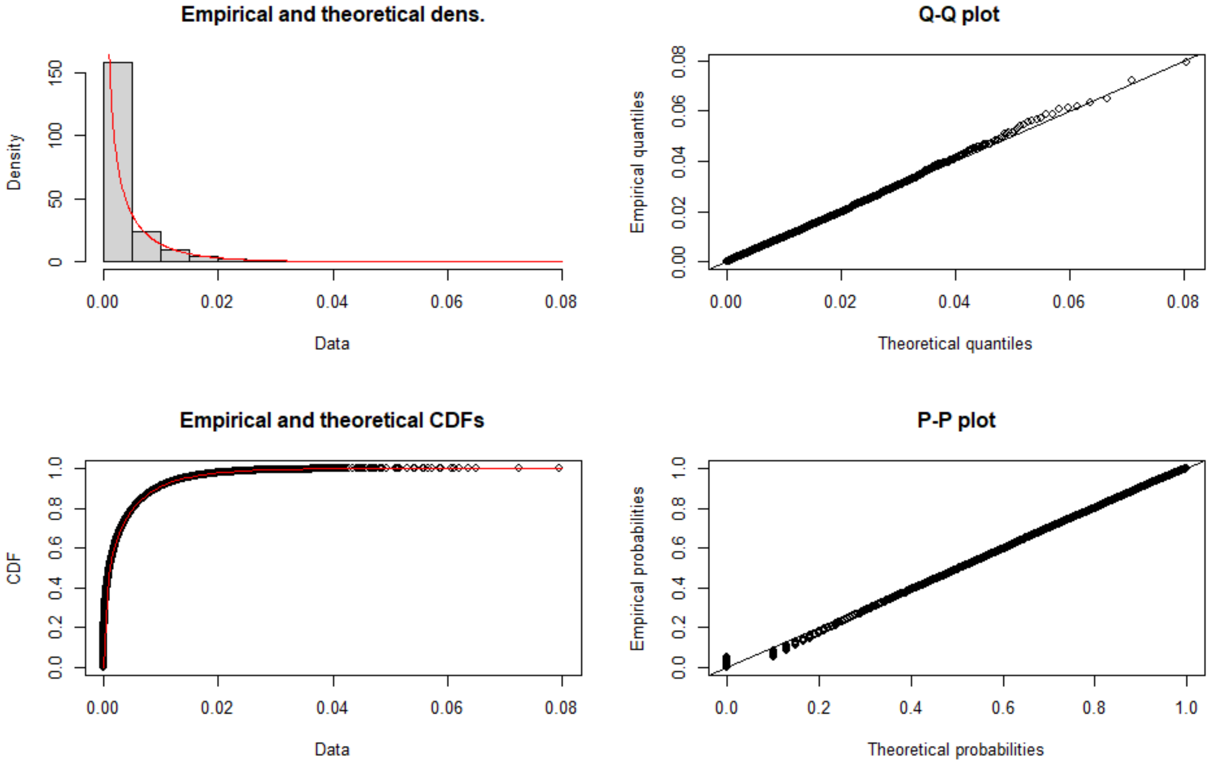
Empirical and theoretical dens.
Density
0
50
100
150
0.00
0.02
0.04
0.06
0.08
Data
Q-Q plot
Empirical quantiles
0.00
0.02
0.04
0.06
0.08
Theoretical quantiles
Empirical and theoretical CDFs
CDF
0.0
0.2
0.4
0.6
0.8
1.0
Data
P-P plot
Empirical probabilities
Theoretical probabilities

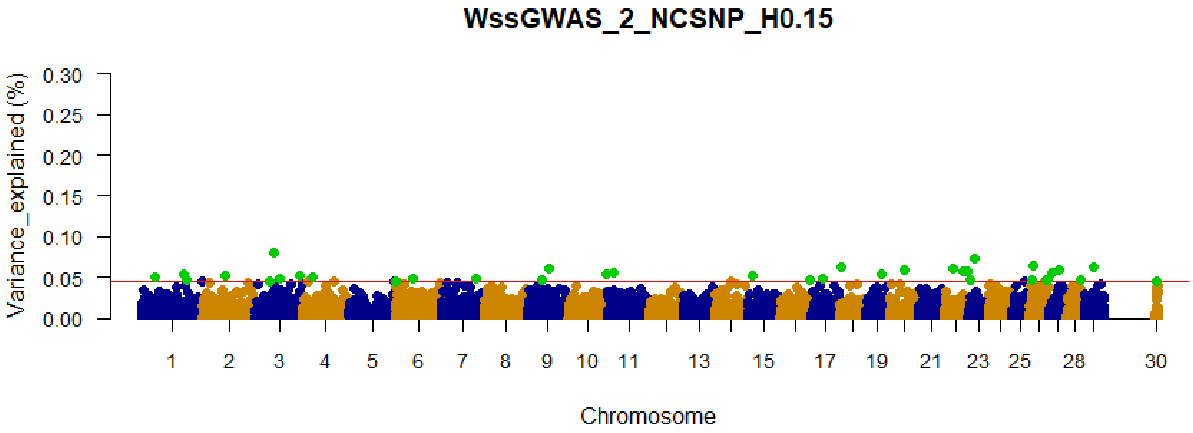
WssGWAS_2_NCSNP_H0.15
Variance_explained (%)
0.00
0.05
0.10
0.15
0.20
0.25
0.30
1
2
3
4
5
6
7
8
9
10
11
13
15
17
19
21
23
25
28
30
Chromosome

Statistics: percentage variance explained by haplotypes and non-clustered SNPs from blocks with LD_0.15
WssGWAS iteration 3 (WssGWAS)

summary statistics
------
min: 0 max: 0.07761
median: 0.00106
mean: 0.003277773
estimated sd: 0.005503608
estimated skewness: 3.448596
estimated kurtosis: 20.62088

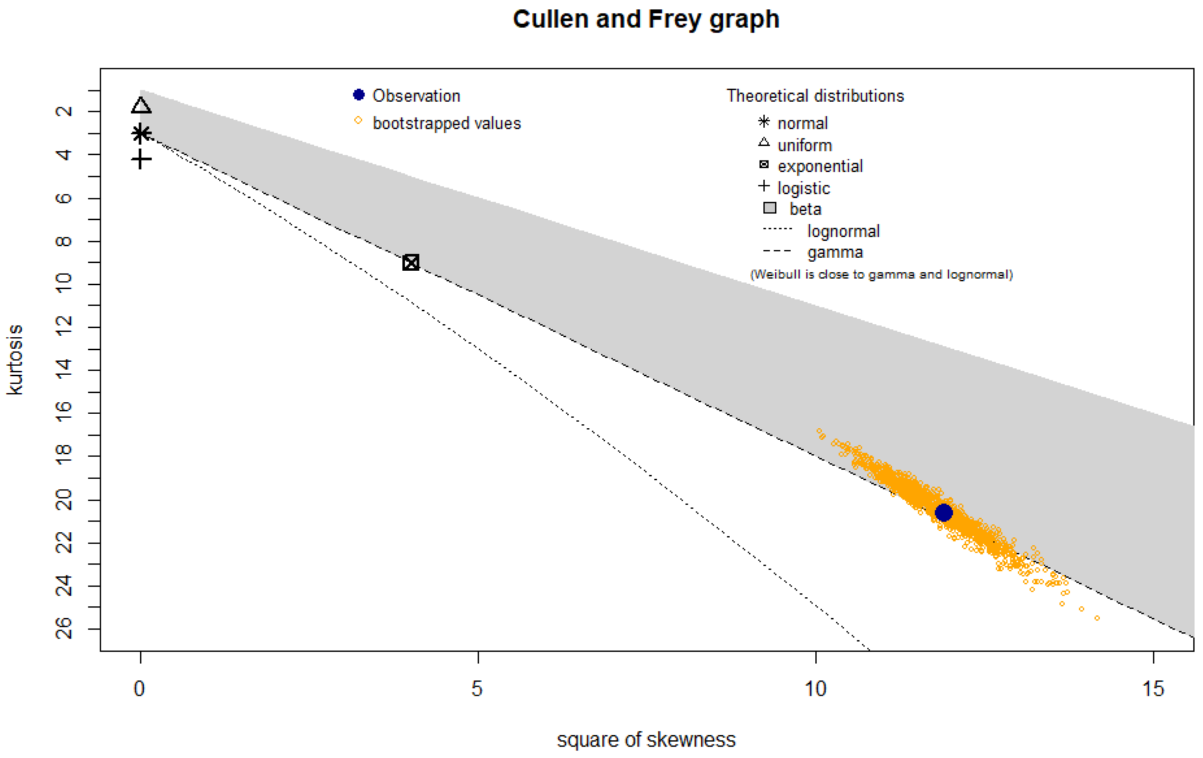

Fitting of the distribution ' gamma ' by matching moments
Parameters :
estimate
shape 0.3547129
rate 108.2176497

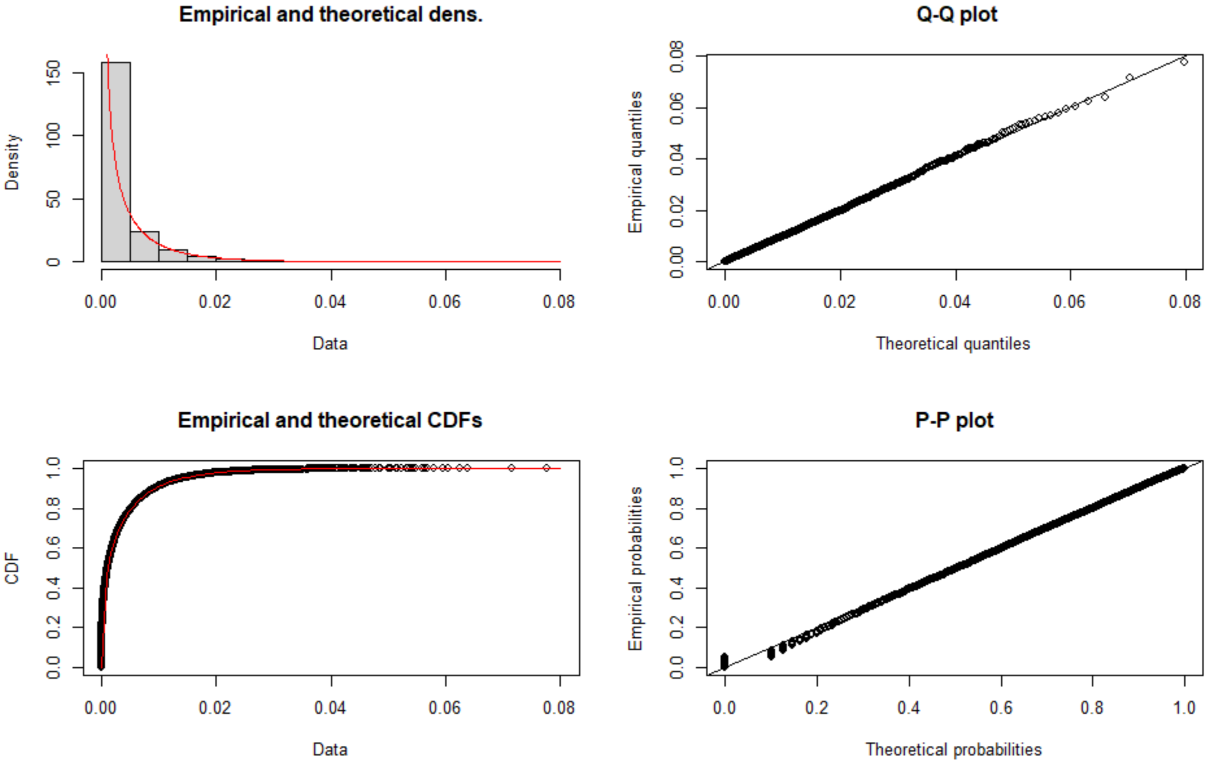

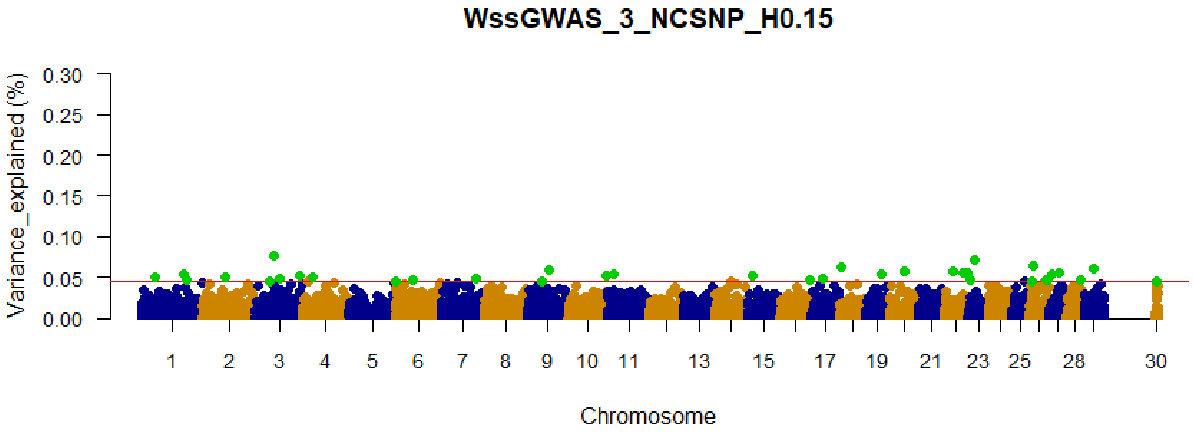

Statistics: percentage variance explained by haplotypes and non-clustered SNPs from blocks with LD_0.50

WssGWAS iteration 1 (ssGWAS)

summary statistics
------
min: 0 max: 0.09132
median: 8e-04
mean: 0.002766918
estimated sd: 0.0051877
estimated skewness: 4.430503
estimated kurtosis: 33.8731

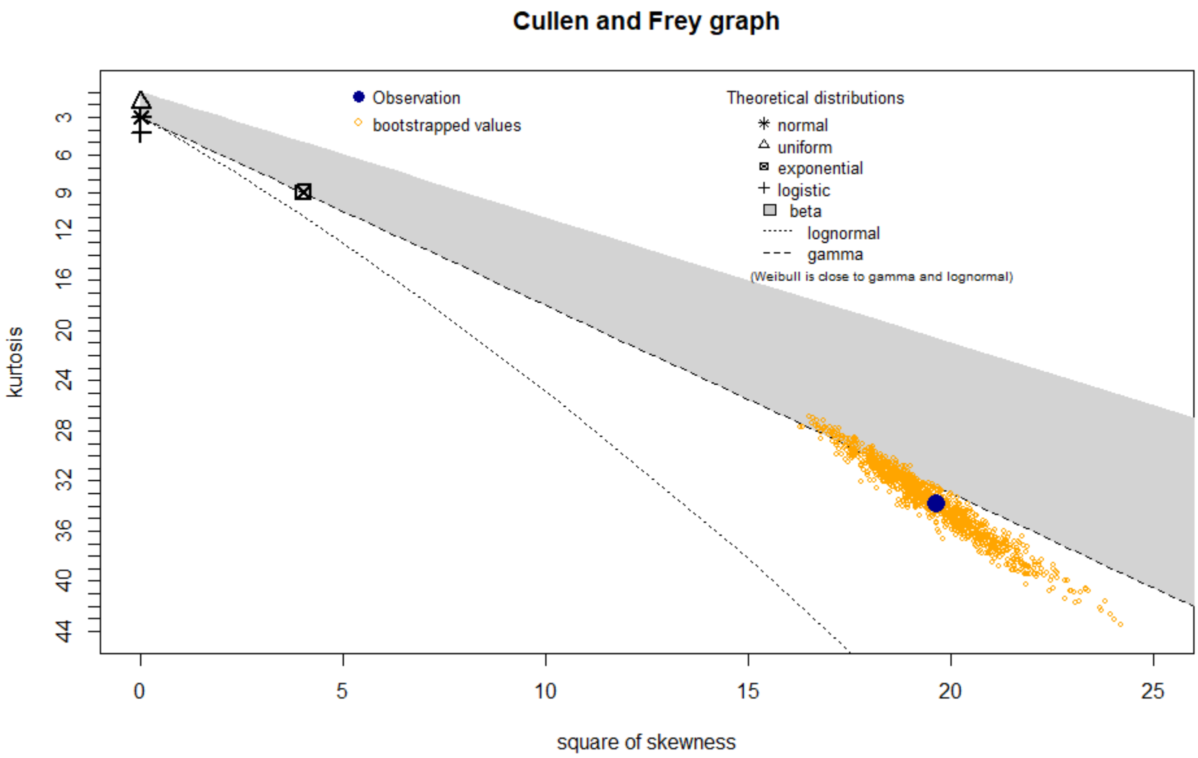

Fitting of the distribution ' gamma ' by matching moments
Parameters :
estimate
shape 0.284482
rate 102.815498

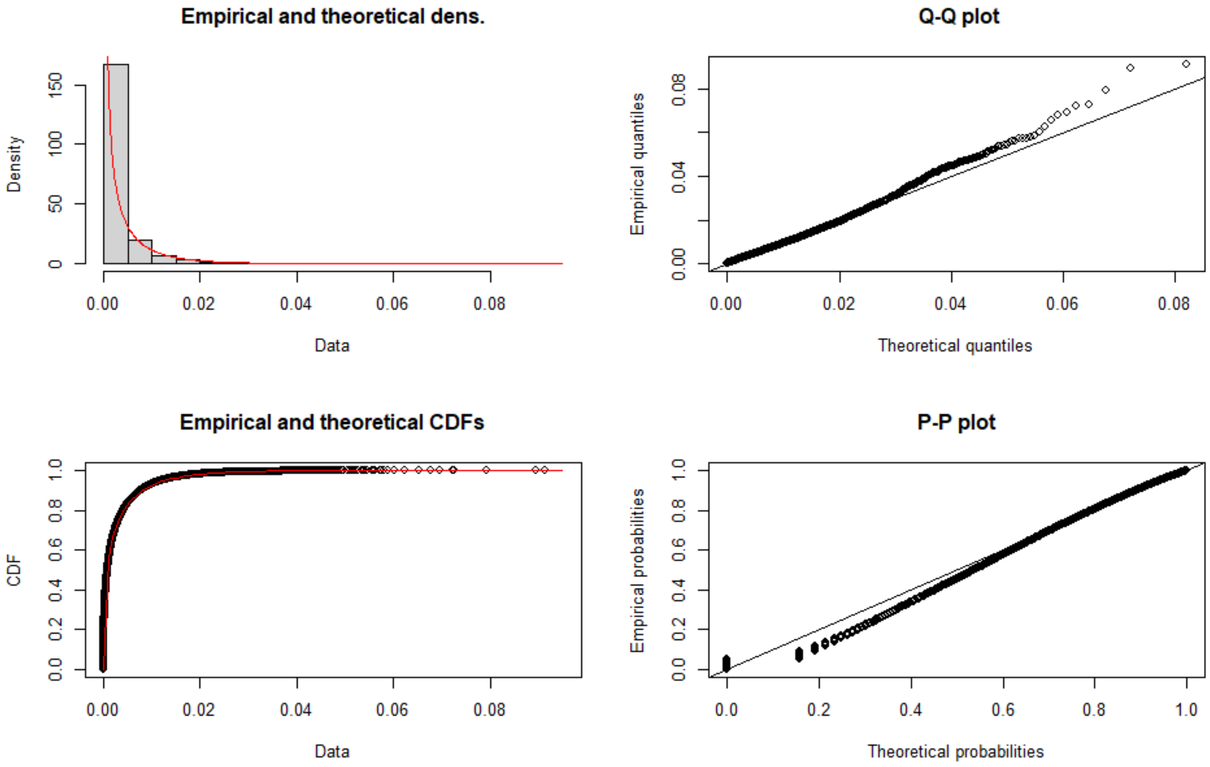
Empirical and theoretical dens.
Density
Data
Q-Q plot
Empirical quantiles
Theoretical quantiles
Empirical and theoretical CDFs
CDF
Data
P-P plot
Empirical probabilities
Theoretical probabilities

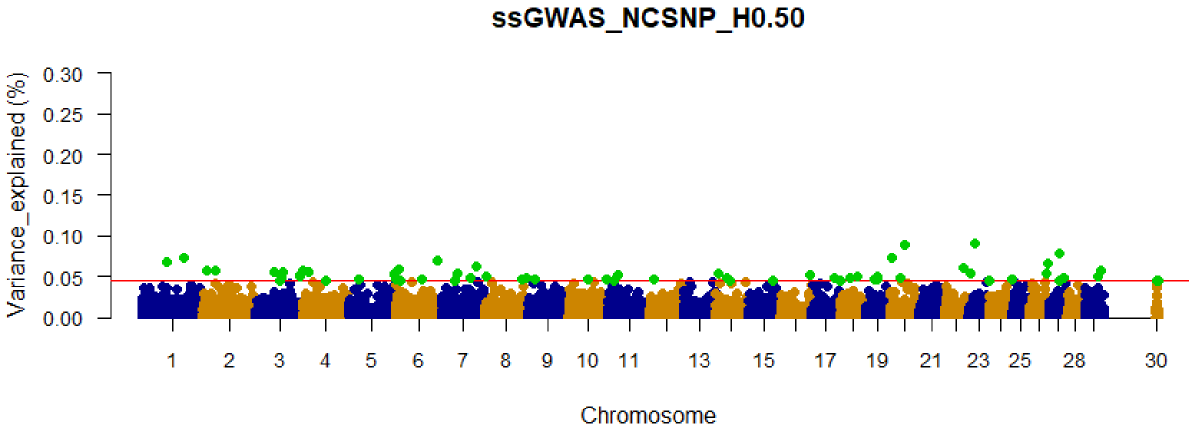
ssGWAS_NCSNP_H0.50
Variance_explained (%)
Chromosome

Statistics: percentage variance explained by haplotypes and non-clustered SNPs from blocks with LD_0.50
WssGWAS iteration 2 (WssGWAS)

summary statistics
------
min: 0   max: 0.0786
median: 0.00084
mean: 0.002766926
estimated sd: 0.004962761
estimated skewness: 3.939182
estimated kurtosis: 26.22129

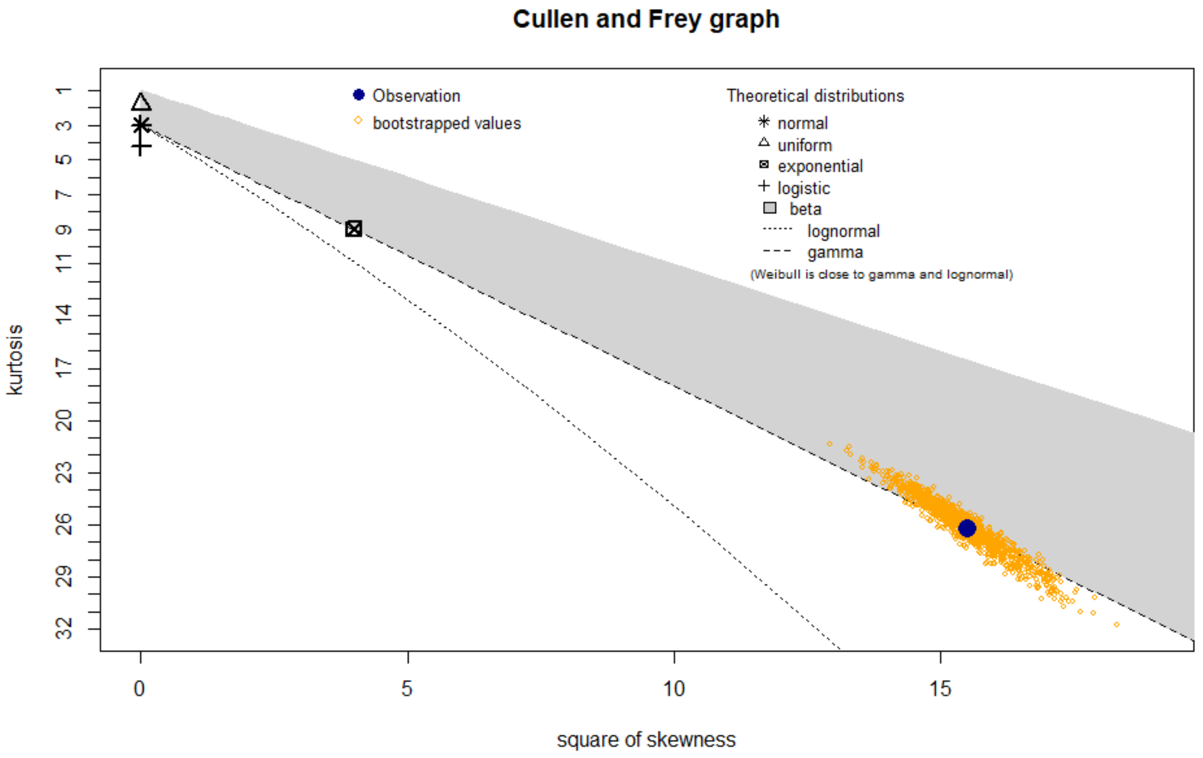

Fitting of the distribution ' gamma ' by matching moments
Parameters :
estimate
shape   0.3108569
rate 112.3473688

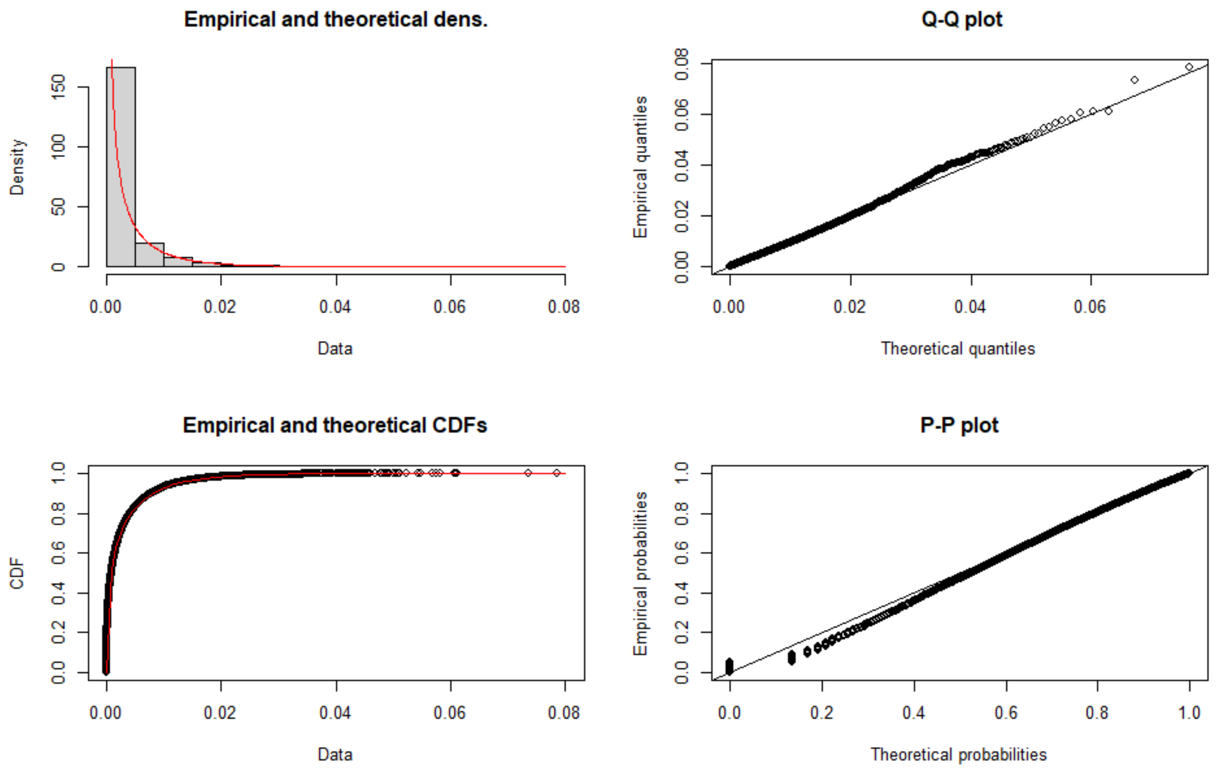
Empirical and theoretical dens.
Density
Data
Q-Q plot
Empirical quantiles
Theoretical quantiles
Empirical and theoretical CDFs
CDF
Data
P-P plot
Empirical probabilities
Theoretical probabilities

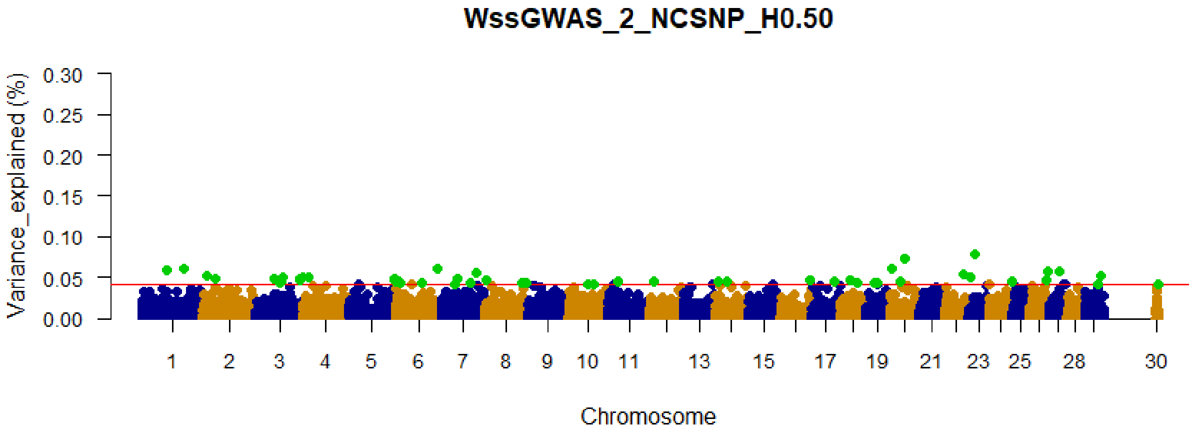
WssGWAS_2_NCSNP_H0.50
Variance_explained (%)
Chromosome

Statistics: percentage variance explained by haplotypes and non-clustered SNPs from blocks with LD_0.50
WssGWAS iteration 3 (WssGWAS)

summary statistics
------
min: 0 max: 0.08061
median: 0.00084
mean: 0.002766898
estimated sd: 0.004986442
estimated skewness: 4.002927
estimated kurtosis: 27.19834

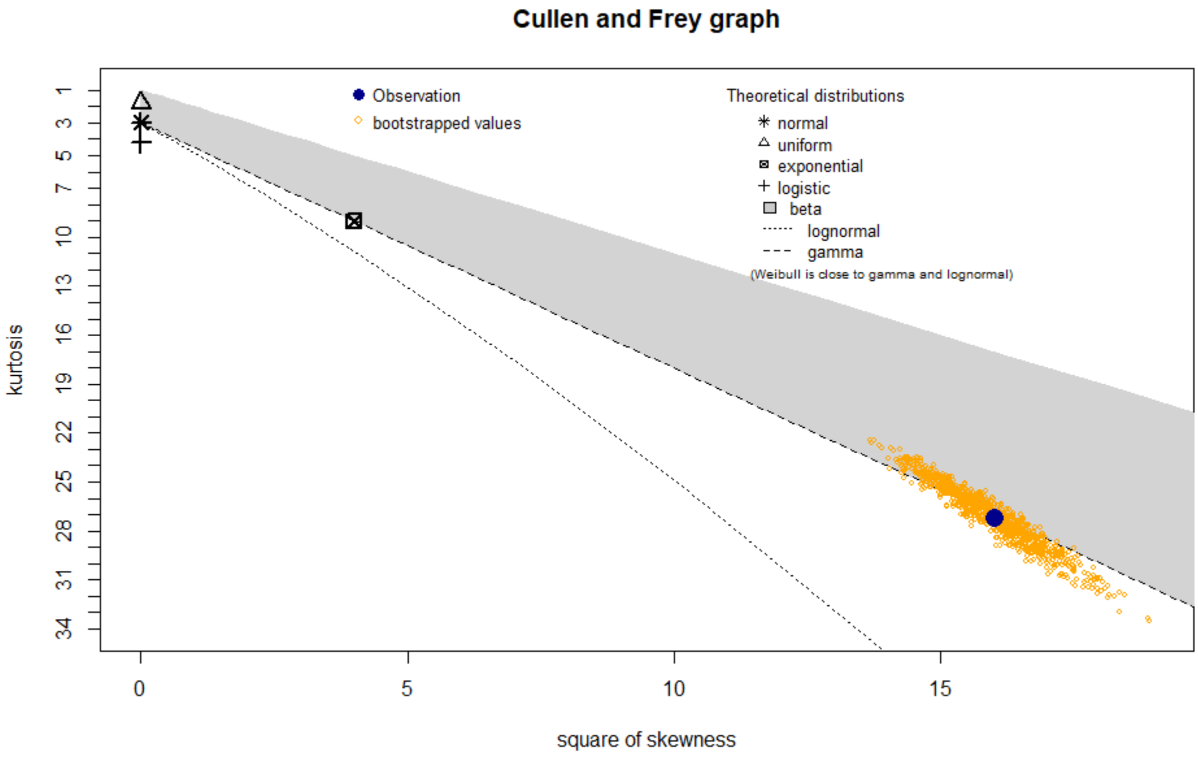

Fitting of the distribution ' gamma ' by matching moments
Parameters :
estimate
shape 0.3079051
rate 111.2816717

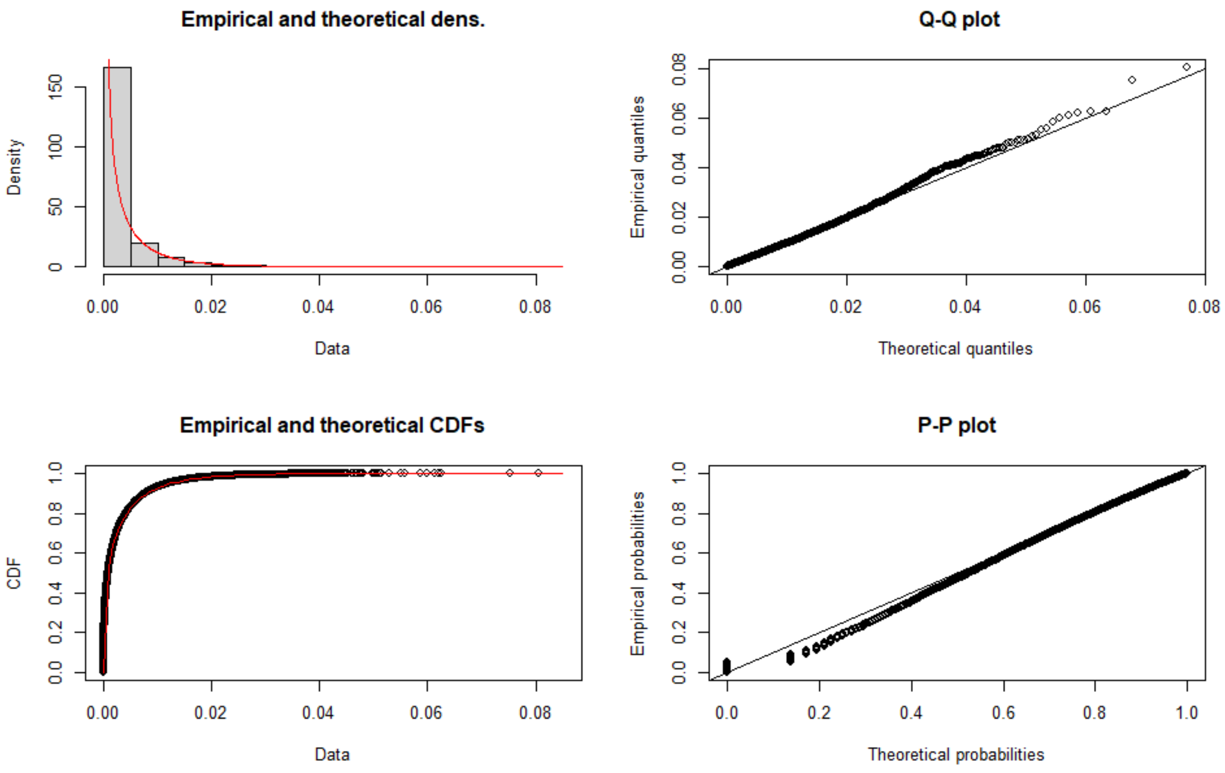

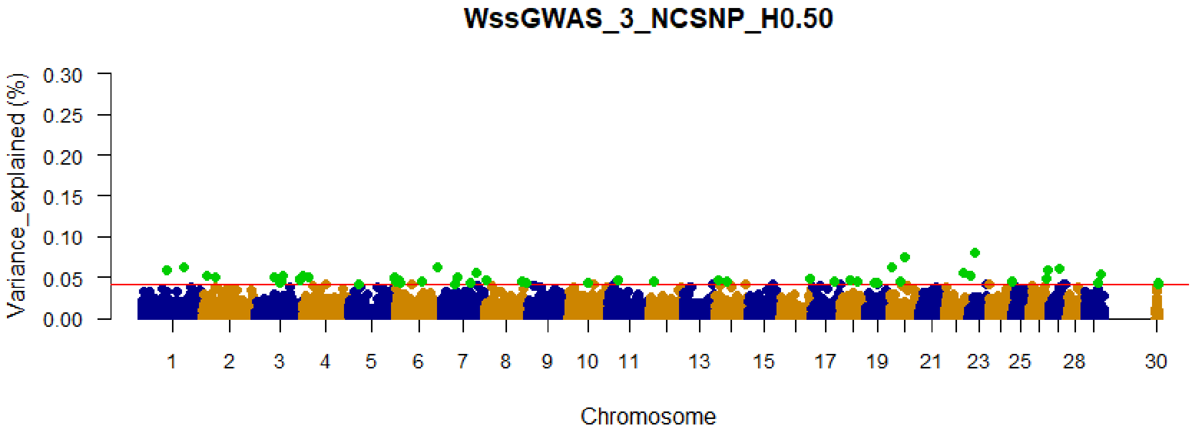

Statistics: percentage variance explained by haplotypes and non-clustered SNPs from blocks with LD_0.80

WssGWAS iteration 1 (ssGWAS)

summary statistics
------
min: 0   max: 0.08546
median: 0.00076
mean: 0.002527993
estimated sd: 0.004725019
estimated skewness: 4.697957
estimated kurtosis: 39.72729

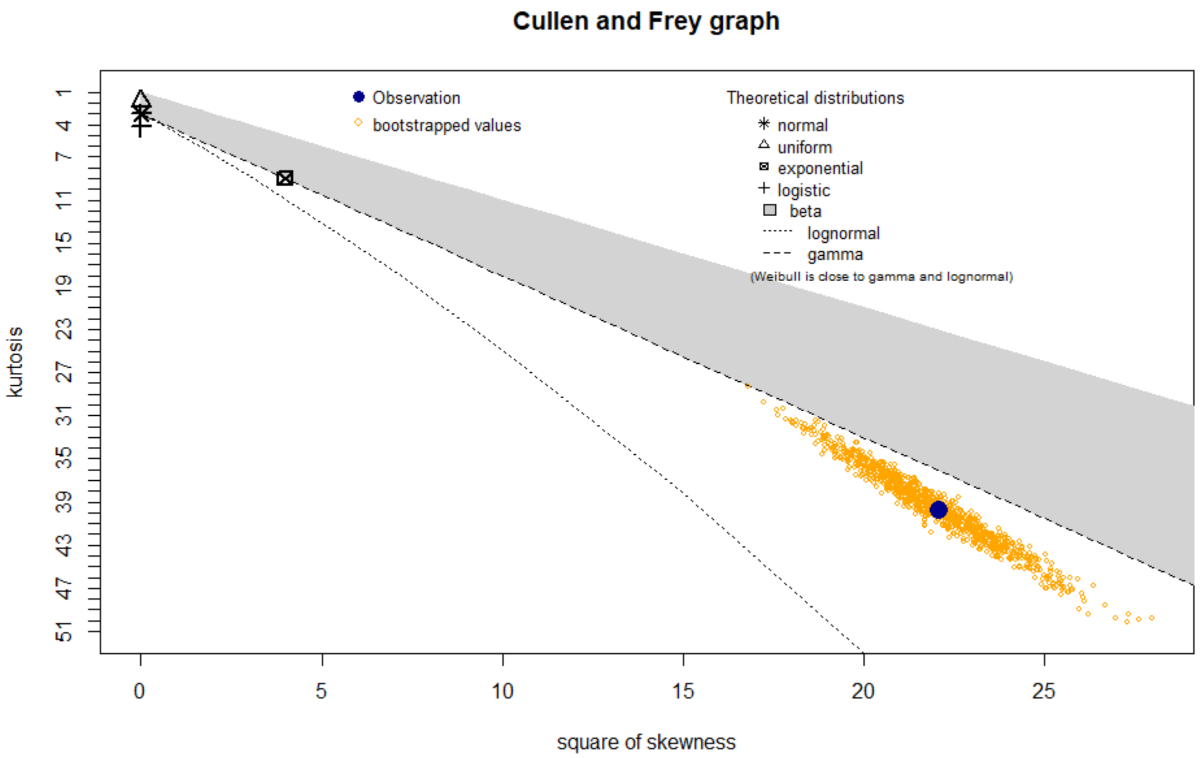

Fitting of the distribution ' gamma ' by matching moments
Parameters :
        estimate
shape   0.2862567
rate 113.2347650

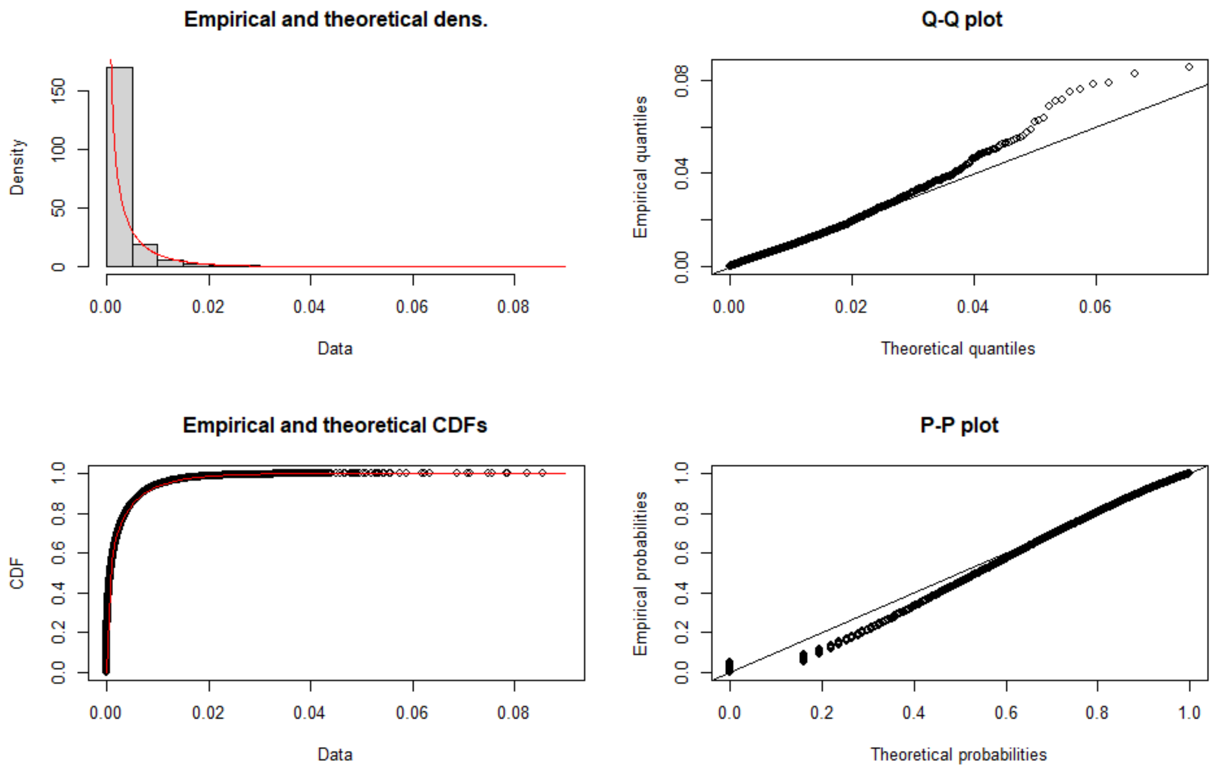
Empirical and theoretical dens.
Density
Data
Q-Q plot
Empirical quantiles
Theoretical quantiles
Empirical and theoretical CDFs
CDF
Data
P-P plot
Empirical probabilities
Theoretical probabilities

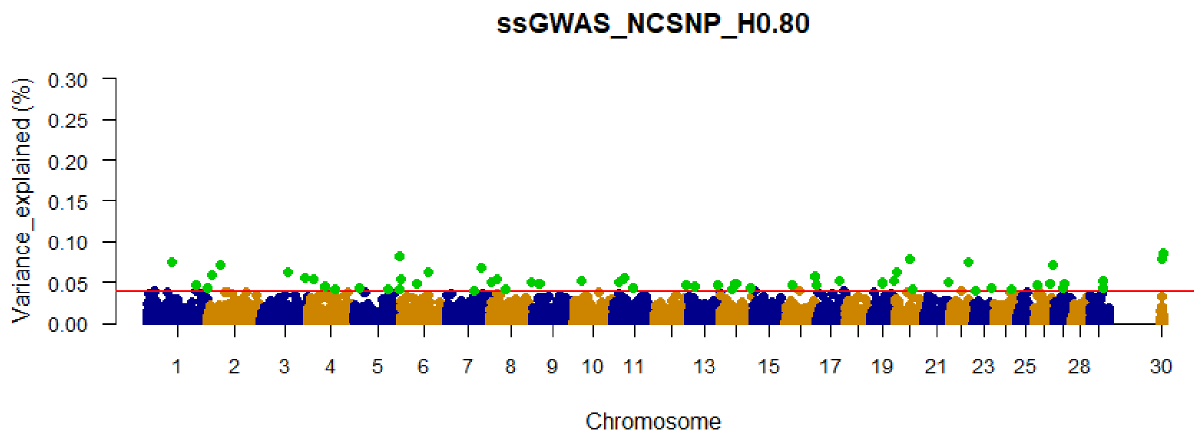
ssGWAS_NCSNP_H0.80
Variance_explained (%)
Chromosome

Statistics: percentage variance explained by haplotypes and non-clustered SNPs from blocks with LD_0.80
WssGWAS iteration 2 (WssGWAS)

summary statistics
------
min: 0 max: 0.07488
median: 8e-04
mean: 0.002527975
estimated sd: 0.00451904
estimated skewness: 4.172307
estimated kurtosis: 30.74078

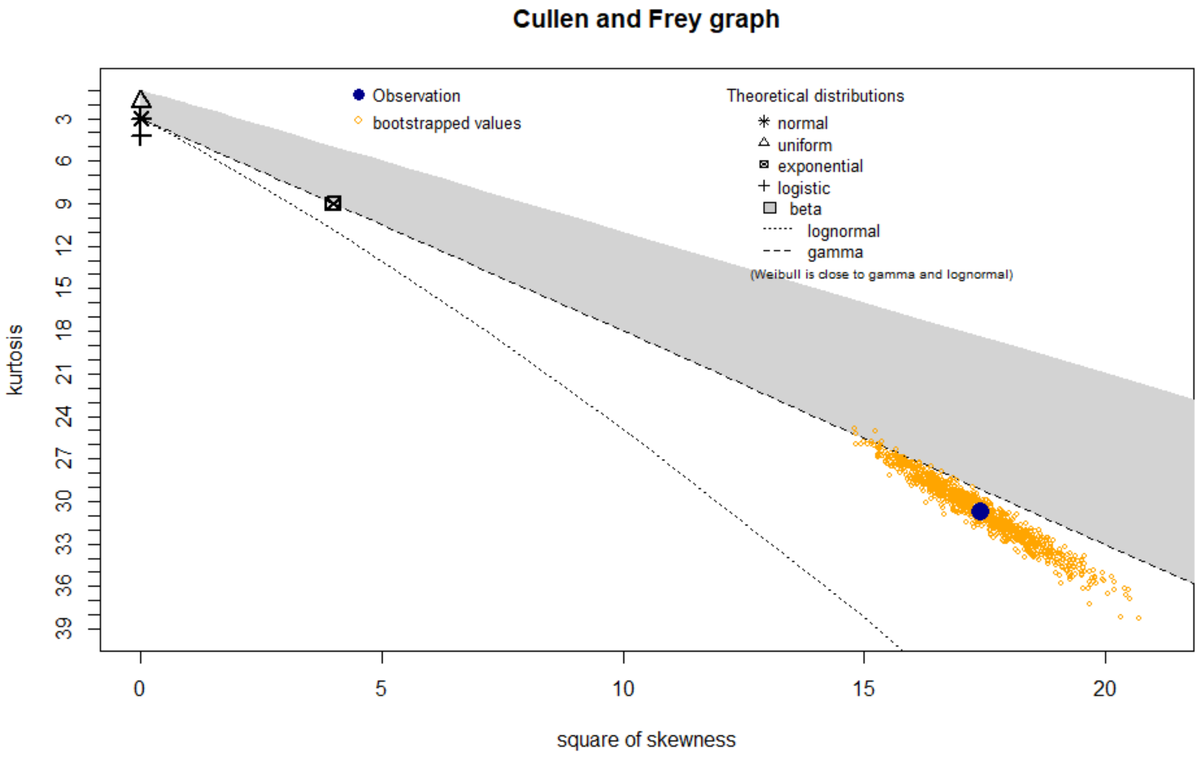

Fitting of the distribution ' gamma ' by matching moments
Parameters :
estimate
shape 0.3129423
rate 123.7916759

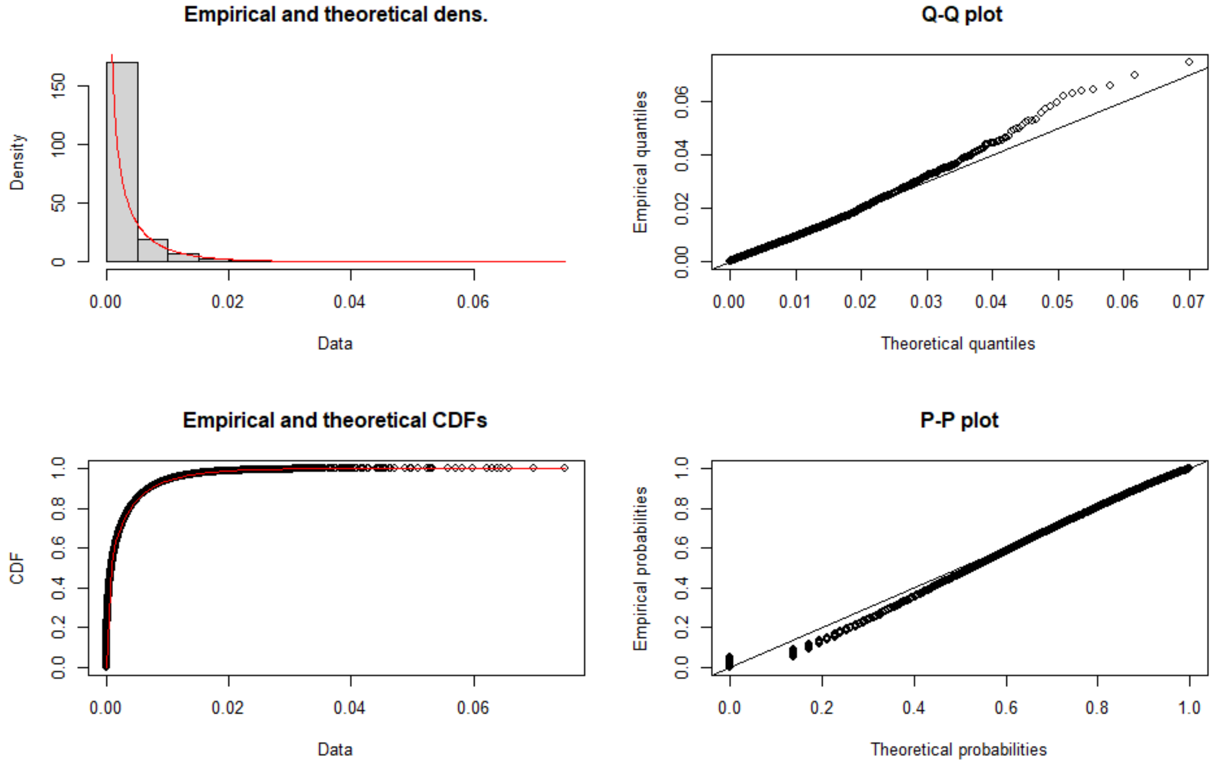
Empirical and theoretical dens.
Density
Data
Q-Q plot
Empirical quantiles
Theoretical quantiles
Empirical and theoretical CDFs
CDF
Data
P-P plot
Empirical probabilities
Theoretical probabilities

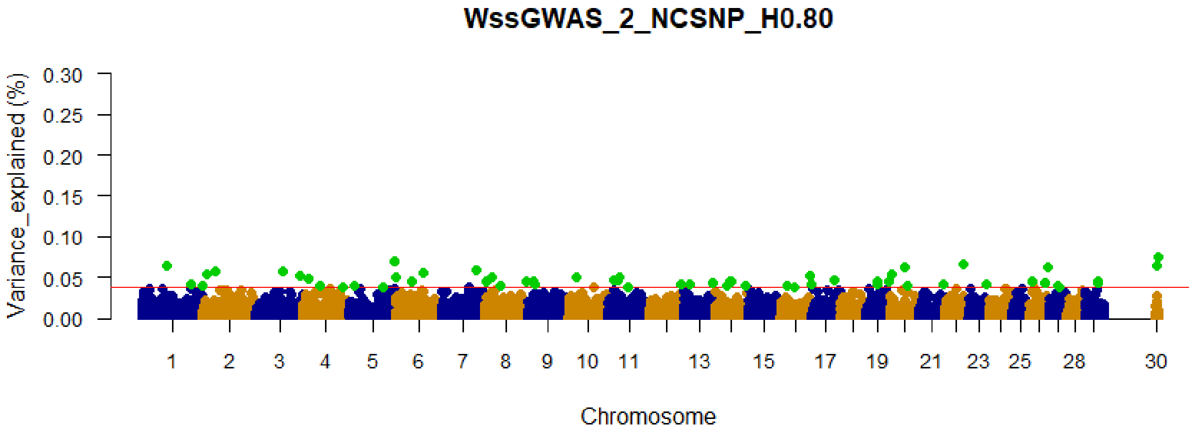
WssGWAS_2_NCSNP_H0.80
Variance_explained (%)
Chromosome

Statistics: percentage variance explained by haplotypes and non-clustered SNPs from blocks with LD_0.80
WssGWAS iteration 3 (WssGWAS)

summary statistics
------
min: 0 max: 0.07663
median: 0.00079
mean: 0.002527986
estimated sd: 0.004541451
estimated skewness: 4.247386
estimated kurtosis: 32.06017

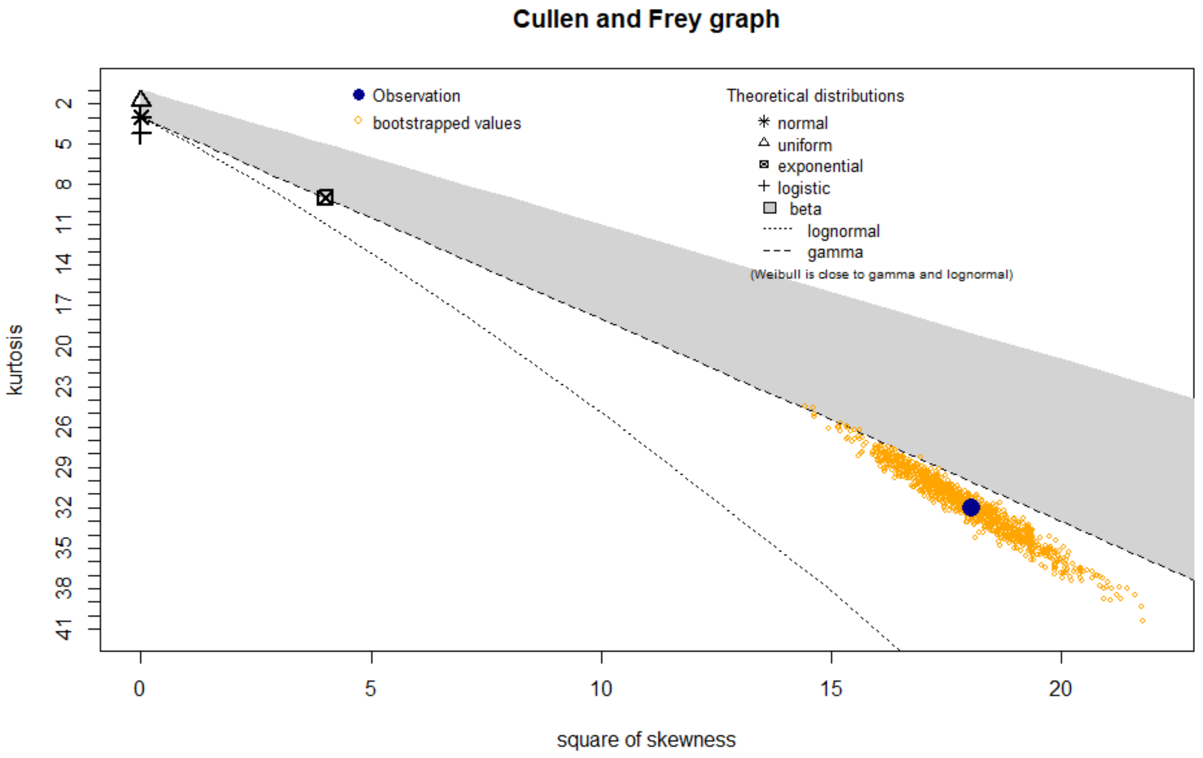

Fitting of the distribution ' gamma ' by matching moments
Parameters :
estimate
shape 0.3098641
rate 122.5734789

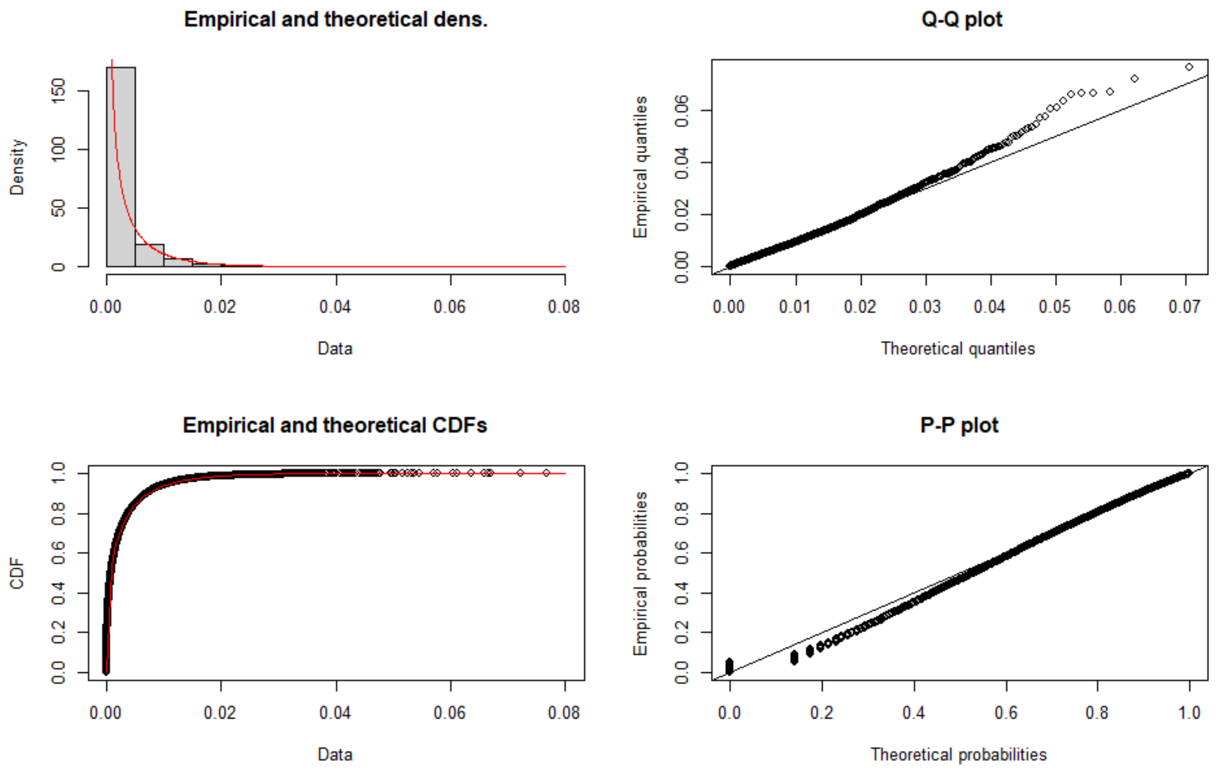
Empirical and theoretical dens.
Density
Data
Q-Q plot
Empirical quantiles
Theoretical quantiles
Empirical and theoretical CDFs
CDF
Data
P-P plot
Empirical probabilities
Theoretical probabilities

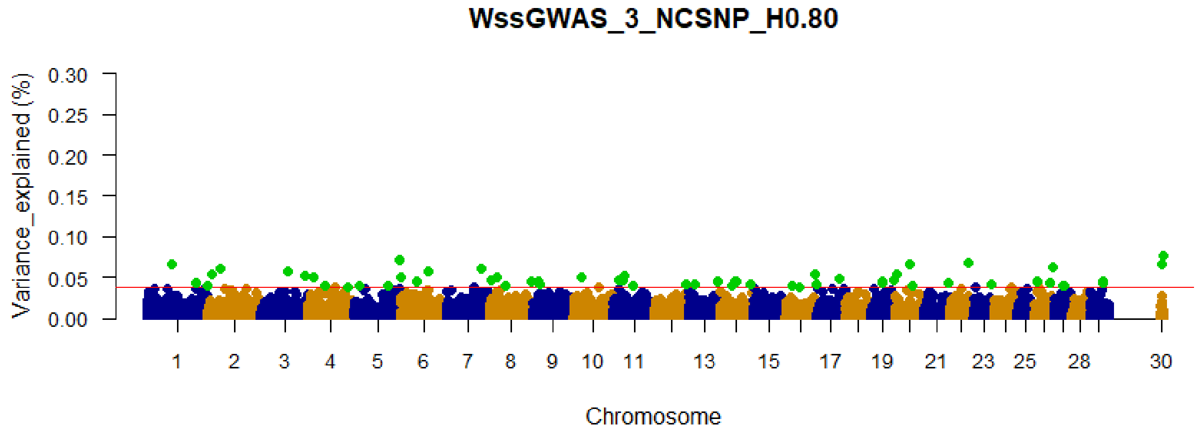
WssGWAS_3_NCSNP_H0.80
Variance_explained (%)
Chromosome
