# Supplementary material for: Haplotype-Based Single-Step GWAS for Yearling Temperament in American Angus Cattle
Source: Genes (Basel). 2021 Dec 22;13(1):17. doi: 10.3390/genes13010017 (PMC8775055; doi:10.3390/genes13010017)
Supplement: Supplementary file 1 [file genes-13-00017-s001.zip › genes-1492001-supplementary/Supplementary_File_S4.pdf]

genes MDPI

Specific and overlapped markers for ssGWAS_H0.15 and ssGWAS_NCSNP_H0.15

- The start SNP names were used to match the overlapped haploblocks across methods because the same region can have different names depending of the LD threshold to create the blocks.

```
$ssGWAS_H0.15
[1] "rs41584803"

$ssGWAS_NCSNP_H0.15
 [1] "rs43227039"  "rs109483880" "rs109668017" "rs109542056" "rs42775050"  "rs110656886" "rs41599805"
 [8] "rs110194000" "rs109134234" "rs42225323"  "rs110710192" "rs41971041"  "rs42814092"  "rs43576496"
[15] "rs41616472"  "rs41664179"  "rs41584520"  "rs43729464"  "rs43350564"  "rs41644452"  "rs110708312"
[22] "rs109450346" "rs110105463" "rs110924756" "rs42437286"  "rs109982860" "rs41580586"  "rs42202984"

$`ssGWAS_H0.15:ssGWAS_NCSNP_H0.15`
 [1] "rs41661378"  "rs42865738"  "rs41624161"  "rs41659143"  "rs29013539"  "rs41605716"  "rs41575824"
 [8] "rs110341524" "rs110151403" "rs42543872"  "rs110904383" "rs42003955"  "rs109152735" "rs41592466"
[15] "rs29013433"  "rs41575769"
```

Specific and overlapped markers for ssGWAS_H0.50 and ssGWAS_NCSNP_H0.50

- The start SNP names were used to match the overlapped haploblocks across methods because the same region can have different names depending of the LD threshold to create the blocks.

```
$ssGWAS_H0.50
[1] "rs42487828"

$ssGWAS_NCSNP_H0.50
 [1] "rs43128174"  "rs43303189"  "rs42775050"  "rs42867969"  "rs42750404"  "rs110783401" "rs110656886"
 [8] "rs109475915" "rs43402375"  "rs110844659" "rs109598646" "rs41654781"  "rs109738132" "rs43015979"
[15] "rs42225323"  "rs110527343" "rs110710192" "rs41575808"  "rs41613284"  "rs110895835" "rs41661749"
[22] "rs29012723"  "rs29012613"  "rs43625164"  "rs110355302" "rs43669894"  "rs110675288" "rs41737077"
[29] "rs41257196"  "rs41664179"  "rs42948217"  "rs109074834" "rs109980096" "rs110250279" "rs109796698"
[36] "rs41620696"  "rs29012545"  "rs43350564"  "rs41644452"  "rs110708312" "rs109152735" "rs109669425"
[43] "rs41668379"  "rs109450346" "rs110105463" "rs41646744"  "rs41584803"  "rs109746481" "rs42187697"
[50] "rs110643010" "rs41579501"  "rs109907786" "rs109460397"

$`ssGWAS_H0.50:ssGWAS_NCSNP_H0.50`
 [1] "rs43238752"  "rs41661378"  "rs43339412"  "rs41659143"  "rs41605716"  "rs42819192"  "rs110572986"
 [8] "rs42382244"  "rs109634189" "rs42543872"
```

Specific and overlapped markers for ssGWAS_H0.80 and ssGWAS_NCSNP_H0.80

- The start SNP names were used to match the overlapped haploblocks across methods because the same region can have different names depending of the LD threshold to create the blocks.

$ssGWAS_H0.80
[1] "rs43626304" "rs110135418" "rs110214513"

$ssGWAS_NCSNP_H0.80
 [1] "rs17871566" "rs43238752" "rs41630032" "rs43128174" "rs43303189" "rs110656886" "rs42750404"
 [8] "rs43389354" "rs41659143" "rs41652995" "rs29018295" "rs109443432" "rs110194000" "rs41577209"
[15] "rs109165576" "rs110710192" "rs41595981" "rs43524272" "rs41620480" "rs43553193" "rs42819192"
[22] "rs29012723" "rs41612816" "rs29012613" "rs109984433" "rs43662934" "rs43669894" "rs43673968"
[29] "rs41588041" "rs110539543" "rs42799141" "rs111003257" "rs41648943" "rs41737077" "rs43576496"
[36] "rs41664179" "rs109346658" "rs41818125" "rs42948217" "rs109796698" "rs110466604" "rs41620696"
[43] "rs41948025" "rs43350564" "rs29011028" "rs41644452" "rs42037449" "rs110708312" "rs110625017"
[50] "rs42082618" "rs110105463" "rs110109654" "rs43331419" "rs41584803" "rs109976529" "rs110643010"
[57] "rs110586411" "rs109460397"

$`ssGWAS_H0.80:ssGWAS_NCSNP_H0.80`
[1] "rs41605716" "rs42382244" "rs42543872"

Specific and overlapped markers considering all ssGWAS using haplotypes only (ssGWAS_H0.15, ssGWAS_H0.50, and ssGWAS_H0.80)

- The start SNP names were used to match the overlapped haploblocks across methods because the same region can have different names depending of the LD threshold to create the blocks.

$ssGWAS_H0.15
 [1] "rs42865738" "rs41624161" "rs29013539" "rs41575824" "rs110341524" "rs110151403" "rs110904383"
 [8] "rs42003955" "rs109152735" "rs41592466" "rs29013433" "rs41584803" "rs41575769"

$ssGWAS_H0.50
[1] "rs43238752" "rs43339412" "rs42487828" "rs42819192" "rs110572986" "rs109634189"

$ssGWAS_H0.80
[1] "rs43626304" "rs110135418" "rs110214513"

$`ssGWAS_H0.15:ssGWAS_H0.50`
[1] "rs41661378" "rs41659143"

$`ssGWAS_H0.50:ssGWAS_H0.80`
[1] "rs42382244"

$`ssGWAS_H0.15:ssGWAS_H0.50:ssGWAS_H0.80`
[1] "rs41605716" "rs42543872"

Specific and overlapped markers considering all ssGWAS using non-clustered SNPs and haplotypes (ssGWAS_NCSNP_H0.15, ssGWAS_NCSNP_H0.50, and ssGWAS_NCSNP_H0.80)

- The start SNP names were used to match the overlapped haploblocks across methods because the same region can have different names depending of the LD threshold to create the blocks.

$ssGWAS_NCSNP_H0.15
 [1] "rs43227039" "rs109483880" "rs109668017" "rs42865738" "rs109542056" "rs41624161" "rs41599805"
 [8] "rs29013539" "rs109134234" "rs41971041" "rs41575824" "rs42814092" "rs110341524" "rs41616472"
[15] "rs41584520" "rs110151403" "rs43729464" "rs110904383" "rs42003955" "rs110924756" "rs41592466"
[22] "rs42437286" "rs29013433" "rs109982860" "rs41575769" "rs41580586" "rs42202984"

$ssGWAS_NCSNP_H0.50
 [1] "rs42867969" "rs110783401" "rs109475915" "rs43339412" "rs43402375" "rs110844659" "rs109598646"
 [8] "rs41654781" "rs109738132" "rs43015979" "rs110527343" "rs41575808" "rs41613284" "rs110895835"
[15] "rs41661749" "rs43625164" "rs110572986" "rs110355302" "rs110675288" "rs109634189" "rs41257196"
[22] "rs109074834" "rs109980096" "rs110250279" "rs29012545" "rs109669425" "rs41668379" "rs41646744"
[29] "rs109746481" "rs42187697" "rs41579501" "rs109907786"

$ssGWAS_NCSNP_H0.80
 [1] "rs17871566" "rs41630032" "rs43389354" "rs41652995" "rs29018295" "rs109443432" "rs41577209"
 [8] "rs109165576" "rs41595981" "rs43524272" "rs41620480" "rs43553193" "rs41612816" "rs109984433"
[15] "rs43662934" "rs43673968" "rs41588041" "rs110539543" "rs42799141" "rs111003257" "rs41648943"
[22] "rs109346658" "rs41818125" "rs110466604" "rs41948025" "rs29011028" "rs42037449" "rs110625017"
[29] "rs42082618" "rs110109654" "rs43331419" "rs109976529" "rs110586411"

$`ssGWAS_NCSNP_H0.15:ssGWAS_NCSNP_H0.50`
[1] "rs41661378" "rs42775050" "rs42225323" "rs109152735" "rs109450346"

$`ssGWAS_NCSNP_H0.15:ssGWAS_NCSNP_H0.80`
[1] "rs110194000" "rs43576496"

$`ssGWAS_NCSNP_H0.50:ssGWAS_NCSNP_H0.80`
 [1] "rs43238752" "rs43128174" "rs43303189" "rs42750404" "rs42819192" "rs29012723" "rs29012613"
 [8] "rs43669894" "rs41737077" "rs42382244" "rs42948217" "rs109796698" "rs41620696" "rs41584803"
[15] "rs110643010" "rs109460397"

$`ssGWAS_NCSNP_H0.15:ssGWAS_NCSNP_H0.50:ssGWAS_NCSNP_H0.80`
 [1] "rs110656886" "rs41659143" "rs110710192" "rs41605716" "rs41664179" "rs42543872" "rs43350564"
 [8] "rs41644452" "rs110708312" "rs110105463"
