# Supplementary material for: Haplotype-Based Single-Step GWAS for Yearling Temperament in American Angus Cattle
Source: Genes (Basel). 2021 Dec 22;13(1):17. doi: 10.3390/genes13010017 (PMC8775055; doi:10.3390/genes13010017)
Supplement: Supplementary file 1 [file genes-13-00017-s001.zip › genes-1492001-supplementary/Supplementary_File_S5.pdf]

genes

MDPI

Specific and overlapped genes for ssGWAS_H0.15 and ssGWAS_NCSNP_H0.15

$`ssGWAS_H0.15`

$ssGWAS_NCSNP_H0.15
[1] "P2RY12" "MED12L" "KCNH8" "SUGCT" "misc_RNA" "IL7" "PTPRC" "KRT31" "KRT34"
[10] "CACNA2D3" "HMGCLL1" "DOCK1" "MGMT" "NRG3"

$`ssGWAS_H0.15:ssGWAS_NCSNP_H0.15`
[1] "5S_rRNA" "UMAD1" "FUT9" "VAX2" "ATP6V1B1" "ANKRD53" "MLKL" "FA2H" "SPEF2"
[10] "PLXNA1" "FOXP4"

Specific and overlapped genes for ssGWAS_H0.50 and ssGWAS_NCSNP_H0.50

$ssGWAS_H0.50
[1] "COMMD10"

$ssGWAS_NCSNP_H0.50
[1] "7SK" "CSMD2" "PPP1R7" "SEPTIN7" "U6" "ATXN10" "ATXN10" "C2CD4C" "BCKDHB"
[10] "ADAM10" "TEX261" "ATP6V1B1" "ANKRD53" "THADA" "AKAP11" "MYBL1" "U6" "ABTB2"
[19] "CAPRIN1" "NAT10" "PTPRC" "KSR2" "UPB1" "SNRPD3" "GUCD1" "ADGRG1" "CEP89"
[28] "ZZEF1" "CACNA2D3" "HMGCLL1" "FOXP4" "DOCK1" "MGMT" "MFHAS1" "SH2D4A" "ADAMTS8"
[37] "SLC3A2" "SNORD25" "SNORD26" "SNORD27" "MXRA5" "CD99"

$`ssGWAS_H0.50:ssGWAS_NCSNP_H0.50`
[1] "RWDD3" "5S_rRNA" "UMAD1" "GALC" "CRISPLD1" "SPEF2"

Specific and overlapped genes for ssGWAS_H0.80 and ssGWAS_NCSNP_H0.80

$ssGWAS_H0.80
[1] "NID2" "PLXDC1" "DOCK1"

$ssGWAS_NCSNP_H0.80
[1] "PCOLCE2" "KCNH8" "7SK" "5S_rRNA" "UMAD1" "FBXL13" "LRRC17" "ATXN10" "GBA3"
[10] "U6" "KANK1" "BCKDHB" "ZNF638" "THADA" "NCOA3" "MYBL1" "U6" "IL7"
[19] "PTPRC" "KSR2" "PPP2R5C" "CACNA2D3" "HMGCLL1" "RREB1" "PRKG1" "MGMT" "DLC1"
[28] "OPCML" "ADAMTS8" "MXRA5"

$`ssGWAS_H0.80:ssGWAS_NCSNP_H0.80`
[1] "CRISPLD1" "SPEF2"

Specific and overlapped genes considering all ssGWAS using haplotypes only (ssGWAS_H0.15, ssGWAS_H0.50, and ssGWAS_H0.80)

$ssGWAS_H0.15
[1] "FUT9" "VAX2" "ATP6V1B1" "ANKRD53" "MLKL" "FA2H" "PLXNA1" "FOXP4"

```
$ssGWAS_H0.50
[1] "RWDD3"   "COMMD10" "GALC"

$ssGWAS_H0.80
[1] "NID2"   "PLXDC1" "DOCK1"

$`ssGWAS_H0.15:ssGWAS_H0.50`
[1] "5S_rRNA" "UMAD1"

$`ssGWAS_H0.50:ssGWAS_H0.80`
[1] "CRISPLD1"

$`ssGWAS_H0.15:ssGWAS_H0.50:ssGWAS_H0.80`
[1] "SPEF2"
```

Specific and overlapped genes considering all ssGWAS using non-clustered SNPs and haplotypes (ssGWAS_NCSNP_H0.15, ssGWAS_NCSNP_H0.50, and ssGWAS_NCSNP_H0.80)

```
$ssGWAS_NCSNP_H0.15
 [1] "P2RY12"   "MED12L"   "SUGCT"    "FUT9"     "VAX2"     "misc_RNA" "MLKL"     "FA2H"     "KRT31"
[10] "KRT34"    "PLXNA1"   "NRG3"

$ssGWAS_NCSNP_H0.50
 [1] "RWDD3"    "CSMD2"    "PPP1R7"   "SEPTIN7" "C2CD4C"   "ADAM10"  "GALC"     "TEX261"  "AKAP11"  "ABTB2"
[11] "CAPRIN1" "NAT10"    "UPB1"     "SNRPD3"  "GUCD1"    "ADGRG1"  "CEP89"    "ZZEF1"    "MFHAS1"  "SH2D4A"
[21] "SLC3A2"  "SNORD25" "SNORD26" "SNORD27" "CD99"

$ssGWAS_NCSNP_H0.80
 [1] "PCOLCE2" "FBXL13"  "LRRC17"  "GBA3"    "KANK1"   "ZNF638"  "NCOA3"   "PPP2R5C" "RREB1"   "PRKG1"
[11] "DLC1"    "OPCML"

$`ssGWAS_NCSNP_H0.15:ssGWAS_NCSNP_H0.50`
[1] "ATP6V1B1" "ANKRD53"  "FOXP4"    "DOCK1"

$`ssGWAS_NCSNP_H0.15:ssGWAS_NCSNP_H0.80`
[1] "KCNH8" "IL7"

$`ssGWAS_NCSNP_H0.50:ssGWAS_NCSNP_H0.80`
 [1] "7SK"      "U6"       "ATXN10"   "ATXN10"   "BCKDHB"   "THADA"    "MYBL1"    "U6"       "CRISPLD1"
[10] "KSR2"     "ADAMTS8"  "MXRA5"

$`ssGWAS_NCSNP_H0.15:ssGWAS_NCSNP_H0.50:ssGWAS_NCSNP_H0.80`
[1] "5S_rRNA"  "UMAD1"    "PTPRC"    "SPEF2"    "CACNA2D3" "HMGCLL1"  "MGMT"
```
