# Supplementary material for: Haplotype-Based Single-Step GWAS for Yearling Temperament in American Angus Cattle
Source: Genes (Basel). 2021 Dec 22;13(1):17. doi: 10.3390/genes13010017 (PMC8775055; doi:10.3390/genes13010017)
Supplement: Supplementary file 1 [file genes-13-00017-s001.zip › genes-1492001-supplementary/Supplementary_File_S8.pdf]

genes

MDPI

Specific and overlapped QTL for ssGWAS_H0.15 and ssGWAS_NCSNP_H0.15

$`ssGWAS_H0.15`

$ssGWAS_NCSNP_H0.15
[1] "QTL_ID=212443" "QTL_ID=25762" "QTL_ID=28109" "QTL_ID=28112" "QTL_ID=64006" "QTL_ID=64007"
[7] "QTL_ID=41728" "QTL_ID=41731" "QTL_ID=41732" "QTL_ID=41737" "QTL_ID=41738" "QTL_ID=41739"
[13] "QTL_ID=41741" "QTL_ID=41742" "QTL_ID=41743" "QTL_ID=41729" "QTL_ID=41733" "QTL_ID=41735"
[19] "QTL_ID=41736" "QTL_ID=41726" "QTL_ID=41730" "QTL_ID=41734" "QTL_ID=41740" "QTL_ID=41727"
[25] "QTL_ID=67176" "QTL_ID=67177" "QTL_ID=67178" "QTL_ID=66530" "QTL_ID=67607" "QTL_ID=67608"
[31] "QTL_ID=130701" "QTL_ID=158090" "QTL_ID=175015" "QTL_ID=200165" "QTL_ID=50404" "QTL_ID=50406"
[37] "QTL_ID=50407" "QTL_ID=50409" "QTL_ID=50413" "QTL_ID=50414" "QTL_ID=50415" "QTL_ID=50416"
[43] "QTL_ID=50417" "QTL_ID=50412" "QTL_ID=164127" "QTL_ID=164206" "QTL_ID=164416" "QTL_ID=164436"
[49] "QTL_ID=164476" "QTL_ID=164535" "QTL_ID=176528" "QTL_ID=186241" "QTL_ID=190174" "QTL_ID=50405"
[55] "QTL_ID=50408" "QTL_ID=50410" "QTL_ID=66693" "QTL_ID=50403" "QTL_ID=50411" "QTL_ID=157031"
[61] "QTL_ID=157030" "QTL_ID=14785" "QTL_ID=196414" "QTL_ID=196415" "QTL_ID=196416" "QTL_ID=32552"
[67] "QTL_ID=32611" "QTL_ID=62834" "QTL_ID=199984" "QTL_ID=199985" "QTL_ID=199982" "QTL_ID=199983"
[73] "QTL_ID=199980" "QTL_ID=199981" "QTL_ID=197373" "QTL_ID=197374" "QTL_ID=202582" "QTL_ID=208787"
[79] "QTL_ID=205130" "QTL_ID=211354" "QTL_ID=201804" "QTL_ID=207725" "QTL_ID=202223" "QTL_ID=208286"
[85] "QTL_ID=204991" "QTL_ID=211233" "QTL_ID=196073" "QTL_ID=113783" "QTL_ID=116938" "QTL_ID=131376"
[91] "QTL_ID=131377" "QTL_ID=131378"

$`ssGWAS_H0.15:ssGWAS_NCSNP_H0.15`
[1] "QTL_ID=39799" "QTL_ID=39795" "QTL_ID=39796" "QTL_ID=39798" "QTL_ID=105587" "QTL_ID=39797"
[7] "QTL_ID=5321" "QTL_ID=39794" "QTL_ID=39800" "QTL_ID=26726" "QTL_ID=43369" "QTL_ID=43370"
[13] "QTL_ID=43372" "QTL_ID=43373" "QTL_ID=43374" "QTL_ID=43375" "QTL_ID=43376" "QTL_ID=43378"
[19] "QTL_ID=43379" "QTL_ID=43380" "QTL_ID=43381" "QTL_ID=43383" "QTL_ID=43384" "QTL_ID=43386"
[25] "QTL_ID=43387" "QTL_ID=43388" "QTL_ID=43389" "QTL_ID=43390" "QTL_ID=43392" "QTL_ID=43393"
[31] "QTL_ID=43394" "QTL_ID=43395" "QTL_ID=43368" "QTL_ID=43371" "QTL_ID=43377" "QTL_ID=43382"
[37] "QTL_ID=43385" "QTL_ID=43391" "QTL_ID=49435" "QTL_ID=49436" "QTL_ID=49439" "QTL_ID=49445"
[43] "QTL_ID=49446" "QTL_ID=49449" "QTL_ID=49450" "QTL_ID=49437" "QTL_ID=49440" "QTL_ID=49443"
[49] "QTL_ID=49444" "QTL_ID=49438" "QTL_ID=49441" "QTL_ID=49442" "QTL_ID=106866" "QTL_ID=49434"
[55] "QTL_ID=49447" "QTL_ID=49448" "QTL_ID=65985" "QTL_ID=198366" "QTL_ID=196694" "QTL_ID=201952"
[61] "QTL_ID=197280" "QTL_ID=202502" "QTL_ID=198163" "QTL_ID=203349" "QTL_ID=199795" "QTL_ID=204890"
[67] "QTL_ID=62078"

Specific and overlapped QTL for ssGWAS_H0.50 and ssGWAS_NCSNP_H0.50

$`ssGWAS_H0.50`

$ssGWAS_NCSNP_H0.50
 [1] "QTL_ID=32557" "QTL_ID=2443"   "QTL_ID=66997" "QTL_ID=64006" "QTL_ID=64007" "QTL_ID=24712"
 [7] "QTL_ID=24781" "QTL_ID=160062" "QTL_ID=32345" "QTL_ID=41728" "QTL_ID=41731" "QTL_ID=41732"
[13] "QTL_ID=41737" "QTL_ID=41738" "QTL_ID=41739" "QTL_ID=41741" "QTL_ID=41742" "QTL_ID=41743"
[19] "QTL_ID=41729" "QTL_ID=41733" "QTL_ID=41735" "QTL_ID=41736" "QTL_ID=41726" "QTL_ID=41730"
[25] "QTL_ID=41734" "QTL_ID=41740" "QTL_ID=41727" "QTL_ID=167101" "QTL_ID=67657" "QTL_ID=37348"
[31] "QTL_ID=37364" "QTL_ID=37349" "QTL_ID=37365" "QTL_ID=119123" "QTL_ID=157531" "QTL_ID=106980"
[37] "QTL_ID=107136" "QTL_ID=158047" "QTL_ID=174291" "QTL_ID=174292" "QTL_ID=68759" "QTL_ID=49619"
[43] "QTL_ID=49627" "QTL_ID=49625" "QTL_ID=193757" "QTL_ID=49622" "QTL_ID=49623" "QTL_ID=49618"
[49] "QTL_ID=49620" "QTL_ID=49621" "QTL_ID=49624" "QTL_ID=49626" "QTL_ID=15493" "QTL_ID=15494"
[55] "QTL_ID=50404" "QTL_ID=50406" "QTL_ID=50407" "QTL_ID=50409" "QTL_ID=50413" "QTL_ID=50414"
[61] "QTL_ID=50415" "QTL_ID=50416" "QTL_ID=50417" "QTL_ID=50412" "QTL_ID=164127" "QTL_ID=164206"
[67] "QTL_ID=164416" "QTL_ID=164436" "QTL_ID=164476" "QTL_ID=164535" "QTL_ID=176528" "QTL_ID=186241"
[73] "QTL_ID=190174" "QTL_ID=50405" "QTL_ID=50408" "QTL_ID=50410" "QTL_ID=66693" "QTL_ID=50403"
[79] "QTL_ID=50411" "QTL_ID=157031" "QTL_ID=157030" "QTL_ID=14785" "QTL_ID=65985" "QTL_ID=28176"
[85] "QTL_ID=52253" "QTL_ID=52255" "QTL_ID=52259" "QTL_ID=32336" "QTL_ID=52258" "QTL_ID=52256"
[91] "QTL_ID=52257" "QTL_ID=52254" "QTL_ID=52260" "QTL_ID=52261" "QTL_ID=113783" "QTL_ID=116938"
[97] "QTL_ID=62078" "QTL_ID=148239" "QTL_ID=148375" "QTL_ID=35479" "QTL_ID=35948" "QTL_ID=35698"
[103] "QTL_ID=35967" "QTL_ID=143428" "QTL_ID=143467" "QTL_ID=143466" "QTL_ID=143487" "QTL_ID=143497"
[109] "QTL_ID=143499" "QTL_ID=143522" "QTL_ID=143525" "QTL_ID=143526" "QTL_ID=143528" "QTL_ID=126011"

$`ssGWAS_H0.50:ssGWAS_NCSNP_H0.50`
 [1] "QTL_ID=21147" "QTL_ID=29831" "QTL_ID=29833" "QTL_ID=164356" "QTL_ID=43369" "QTL_ID=43370"
 [7] "QTL_ID=43372" "QTL_ID=43373" "QTL_ID=43374" "QTL_ID=43375" "QTL_ID=43376" "QTL_ID=43378"
[13] "QTL_ID=43379" "QTL_ID=43380" "QTL_ID=43381" "QTL_ID=43383" "QTL_ID=43384" "QTL_ID=43386"
[19] "QTL_ID=43387" "QTL_ID=43388" "QTL_ID=43389" "QTL_ID=43390" "QTL_ID=43392" "QTL_ID=43393"
[25] "QTL_ID=43394" "QTL_ID=43395" "QTL_ID=43368" "QTL_ID=43371" "QTL_ID=43377" "QTL_ID=43382"
[31] "QTL_ID=43385" "QTL_ID=43391" "QTL_ID=65486" "QTL_ID=27090" "QTL_ID=33590" "QTL_ID=33591"
[37] "QTL_ID=33592" "QTL_ID=33593" "QTL_ID=200486" "QTL_ID=205641" "QTL_ID=205642" "QTL_ID=205643"
[43] "QTL_ID=205644" "QTL_ID=205645" "QTL_ID=33759" "QTL_ID=23204" "QTL_ID=14991" "QTL_ID=161733"
[49] "QTL_ID=173725" "QTL_ID=25571" "QTL_ID=25985" "QTL_ID=26607" "QTL_ID=35367" "QTL_ID=35573"
[55] "QTL_ID=24605"

Specific and overlapped QTL for ssGWAS_H0.80 and ssGWAS_NCSNP_H0.80

$ssGWAS_H0.80
[1] "QTL_ID=113783" "QTL_ID=116938"

$ssGWAS_NCSNP_H0.80
 [1] "QTL_ID=21147" "QTL_ID=29831" "QTL_ID=29833" "QTL_ID=32557" "QTL_ID=64006" "QTL_ID=64007"
 [7] "QTL_ID=125442" "QTL_ID=41728" "QTL_ID=41731" "QTL_ID=41732" "QTL_ID=41737" "QTL_ID=41738"
[13] "QTL_ID=41739" "QTL_ID=41741" "QTL_ID=41742" "QTL_ID=41743" "QTL_ID=41729" "QTL_ID=41733"
[19] "QTL_ID=41735" "QTL_ID=41736" "QTL_ID=41726" "QTL_ID=41730" "QTL_ID=41734" "QTL_ID=41740"
[25] "QTL_ID=41727" "QTL_ID=177018" "QTL_ID=42094" "QTL_ID=42095" "QTL_ID=42098" "QTL_ID=42099"
[31] "QTL_ID=42105" "QTL_ID=42106" "QTL_ID=42110" "QTL_ID=42111" "QTL_ID=42112" "QTL_ID=42115"
[37] "QTL_ID=42116" "QTL_ID=42119" "QTL_ID=42120" "QTL_ID=42126" "QTL_ID=42127" "QTL_ID=42131"

[43] "QTL_ID=42132" "QTL_ID=42133" "QTL_ID=42096" "QTL_ID=42100" "QTL_ID=42103" "QTL_ID=42104"
[49] "QTL_ID=42117" "QTL_ID=42121" "QTL_ID=42124" "QTL_ID=42125" "QTL_ID=42092" "QTL_ID=42097"
[55] "QTL_ID=42101" "QTL_ID=42102" "QTL_ID=42107" "QTL_ID=42113" "QTL_ID=42118" "QTL_ID=42122"
[61] "QTL_ID=42123" "QTL_ID=42128" "QTL_ID=181936" "QTL_ID=182379" "QTL_ID=184562" "QTL_ID=185307"
[67] "QTL_ID=185502" "QTL_ID=185746" "QTL_ID=185966" "QTL_ID=187366" "QTL_ID=187474" "QTL_ID=188492"
[73] "QTL_ID=188805" "QTL_ID=189289" "QTL_ID=189510" "QTL_ID=189637" "QTL_ID=42093" "QTL_ID=42108"
[79] "QTL_ID=42109" "QTL_ID=42114" "QTL_ID=42129" "QTL_ID=42130" "QTL_ID=212624" "QTL_ID=178966"
[85] "QTL_ID=68117" "QTL_ID=68118" "QTL_ID=106980" "QTL_ID=107136" "QTL_ID=68310" "QTL_ID=135799"
[91] "QTL_ID=68559" "QTL_ID=176799" "QTL_ID=177143" "QTL_ID=158047" "QTL_ID=158090" "QTL_ID=175015"
[97] "QTL_ID=15493" "QTL_ID=15494" "QTL_ID=50404" "QTL_ID=50406" "QTL_ID=50407" "QTL_ID=50409"
[103] "QTL_ID=50413" "QTL_ID=50414" "QTL_ID=50415" "QTL_ID=50416" "QTL_ID=50417" "QTL_ID=50412"
[109] "QTL_ID=164127" "QTL_ID=164206" "QTL_ID=164416" "QTL_ID=164436" "QTL_ID=164476" "QTL_ID=164535"
[115] "QTL_ID=176528" "QTL_ID=186241" "QTL_ID=190174" "QTL_ID=50405" "QTL_ID=50408" "QTL_ID=50410"
[121] "QTL_ID=66693" "QTL_ID=50403" "QTL_ID=50411" "QTL_ID=165364" "QTL_ID=157031" "QTL_ID=157030"
[127] "QTL_ID=14785" "QTL_ID=107642" "QTL_ID=35479" "QTL_ID=35948"

$`ssGWAS_H0.80:ssGWAS_NCSNP_H0.80`
[1] "QTL_ID=43369" "QTL_ID=43370" "QTL_ID=43372" "QTL_ID=43373" "QTL_ID=43374" "QTL_ID=43375" "QTL_ID=43376"
[8] "QTL_ID=43378" "QTL_ID=43379" "QTL_ID=43380" "QTL_ID=43381" "QTL_ID=43383" "QTL_ID=43384" "QTL_ID=43386"
[15] "QTL_ID=43387" "QTL_ID=43388" "QTL_ID=43389" "QTL_ID=43390" "QTL_ID=43392" "QTL_ID=43393" "QTL_ID=43394"
[22] "QTL_ID=43395" "QTL_ID=43368" "QTL_ID=43371" "QTL_ID=43377" "QTL_ID=43382" "QTL_ID=43385" "QTL_ID=43391"

Specific and overlapped QTL considering all ssGWAS using haplotypes only (ssGWAS_H0.15, ssGWAS_H0.50, and ssGWAS_H0.80)

$ssGWAS_H0.15
[1] "QTL_ID=39799" "QTL_ID=39795" "QTL_ID=39796" "QTL_ID=39798" "QTL_ID=105587" "QTL_ID=39797"
[7] "QTL_ID=5321" "QTL_ID=39794" "QTL_ID=39800" "QTL_ID=26726" "QTL_ID=49435" "QTL_ID=49436"
[13] "QTL_ID=49439" "QTL_ID=49445" "QTL_ID=49446" "QTL_ID=49449" "QTL_ID=49450" "QTL_ID=49437"
[19] "QTL_ID=49440" "QTL_ID=49443" "QTL_ID=49444" "QTL_ID=49438" "QTL_ID=49441" "QTL_ID=49442"
[25] "QTL_ID=106866" "QTL_ID=49434" "QTL_ID=49447" "QTL_ID=49448" "QTL_ID=65985" "QTL_ID=198366"
[31] "QTL_ID=196694" "QTL_ID=201952" "QTL_ID=197280" "QTL_ID=202502" "QTL_ID=198163" "QTL_ID=203349"
[37] "QTL_ID=199795" "QTL_ID=204890" "QTL_ID=62078"

$ssGWAS_H0.50
[1] "QTL_ID=21147" "QTL_ID=29831" "QTL_ID=29833" "QTL_ID=164356" "QTL_ID=65486" "QTL_ID=27090"
[7] "QTL_ID=33590" "QTL_ID=33591" "QTL_ID=33592" "QTL_ID=33593" "QTL_ID=200486" "QTL_ID=205641"
[13] "QTL_ID=205642" "QTL_ID=205643" "QTL_ID=205644" "QTL_ID=205645" "QTL_ID=33759" "QTL_ID=23204"
[19] "QTL_ID=14991" "QTL_ID=161733" "QTL_ID=173725" "QTL_ID=25571" "QTL_ID=25985" "QTL_ID=26607"
[25] "QTL_ID=35367" "QTL_ID=35573" "QTL_ID=24605"

$ssGWAS_H0.80

[1] "QTL_ID=113783" "QTL_ID=116938"

$`ssGWAS_H0.15:ssGWAS_H0.50`

$`ssGWAS_H0.15:ssGWAS_H0.80`

$`ssGWAS_H0.50:ssGWAS_H0.80`

$`ssGWAS_H0.15:ssGWAS_H0.50:ssGWAS_H0.80`
[1] "QTL_ID=43369" "QTL_ID=43370" "QTL_ID=43372" "QTL_ID=43373" "QTL_ID=43374" "QTL_ID=43375" "QTL_ID=43376"
[8] "QTL_ID=43378" "QTL_ID=43379" "QTL_ID=43380" "QTL_ID=43381" "QTL_ID=43383" "QTL_ID=43384" "QTL_ID=43386"
[15] "QTL_ID=43387" "QTL_ID=43388" "QTL_ID=43389" "QTL_ID=43390" "QTL_ID=43392" "QTL_ID=43393" "QTL_ID=43394"
[22] "QTL_ID=43395" "QTL_ID=43368" "QTL_ID=43371" "QTL_ID=43377" "QTL_ID=43382" "QTL_ID=43385" "QTL_ID=43391"

Specific and overlapped QTL considering all ssGWAS using non-clustered SNPs and haplotypes (ssGWAS_NCSNP_H0.15, ssGWAS_NCSNP_H0.50, and ssGWAS_NCSNP_H0.80)

$ssGWAS_NCSNP_H0.15
[1] "QTL_ID=212443" "QTL_ID=25762" "QTL_ID=28109" "QTL_ID=28112" "QTL_ID=39799" "QTL_ID=39795"
[7] "QTL_ID=39796" "QTL_ID=39798" "QTL_ID=105587" "QTL_ID=39797" "QTL_ID=5321" "QTL_ID=39794"
[13] "QTL_ID=39800" "QTL_ID=26726" "QTL_ID=67176" "QTL_ID=67177" "QTL_ID=67178" "QTL_ID=66530"
[19] "QTL_ID=67607" "QTL_ID=67608" "QTL_ID=130701" "QTL_ID=49435" "QTL_ID=49436" "QTL_ID=49439"
[25] "QTL_ID=49445" "QTL_ID=49446" "QTL_ID=49449" "QTL_ID=49450" "QTL_ID=49437" "QTL_ID=49440"
[31] "QTL_ID=49443" "QTL_ID=49444" "QTL_ID=49438" "QTL_ID=49441" "QTL_ID=49442" "QTL_ID=106866"
[37] "QTL_ID=49434" "QTL_ID=49447" "QTL_ID=49448" "QTL_ID=200165" "QTL_ID=196414" "QTL_ID=196415"
[43] "QTL_ID=196416" "QTL_ID=32552" "QTL_ID=32611" "QTL_ID=62834" "QTL_ID=199984" "QTL_ID=199985"
[49] "QTL_ID=199982" "QTL_ID=199983" "QTL_ID=199980" "QTL_ID=199981" "QTL_ID=197373" "QTL_ID=197374"
[55] "QTL_ID=202582" "QTL_ID=208787" "QTL_ID=205130" "QTL_ID=211354" "QTL_ID=201804" "QTL_ID=207725"
[61] "QTL_ID=202223" "QTL_ID=208286" "QTL_ID=204991" "QTL_ID=211233" "QTL_ID=196073" "QTL_ID=198366"
[67] "QTL_ID=196694" "QTL_ID=201952" "QTL_ID=197280" "QTL_ID=202502" "QTL_ID=198163" "QTL_ID=203349"
[73] "QTL_ID=199795" "QTL_ID=204890" "QTL_ID=131376" "QTL_ID=131377" "QTL_ID=131378"

$ssGWAS_NCSNP_H0.50
[1] "QTL_ID=164356" "QTL_ID=2443" "QTL_ID=66997" "QTL_ID=24712" "QTL_ID=24781" "QTL_ID=160062"
[7] "QTL_ID=32345" "QTL_ID=167101" "QTL_ID=67657" "QTL_ID=37348" "QTL_ID=37364" "QTL_ID=37349"
[13] "QTL_ID=37365" "QTL_ID=119123" "QTL_ID=157531" "QTL_ID=65486" "QTL_ID=27090" "QTL_ID=33590"
[19] "QTL_ID=33591" "QTL_ID=33592" "QTL_ID=33593" "QTL_ID=200486" "QTL_ID=205641" "QTL_ID=205642"
[25] "QTL_ID=205643" "QTL_ID=205644" "QTL_ID=205645" "QTL_ID=33759" "QTL_ID=23204" "QTL_ID=14991"
[31] "QTL_ID=161733" "QTL_ID=173725" "QTL_ID=25571" "QTL_ID=25985" "QTL_ID=26607" "QTL_ID=35367"

[37] "QTL_ID=35573" "QTL_ID=24605" "QTL_ID=174291" "QTL_ID=174292" "QTL_ID=68759" "QTL_ID=49619"
[43] "QTL_ID=49627" "QTL_ID=49625" "QTL_ID=193757" "QTL_ID=49622" "QTL_ID=49623" "QTL_ID=49618"
[49] "QTL_ID=49620" "QTL_ID=49621" "QTL_ID=49624" "QTL_ID=49626" "QTL_ID=28176" "QTL_ID=52253"
[55] "QTL_ID=52255" "QTL_ID=52259" "QTL_ID=32336" "QTL_ID=52258" "QTL_ID=52256" "QTL_ID=52257"
[61] "QTL_ID=52254" "QTL_ID=52260" "QTL_ID=52261" "QTL_ID=148239" "QTL_ID=148375" "QTL_ID=35698"
[67] "QTL_ID=35967" "QTL_ID=143428" "QTL_ID=143467" "QTL_ID=143466" "QTL_ID=143487" "QTL_ID=143497"
[73] "QTL_ID=143499" "QTL_ID=143522" "QTL_ID=143525" "QTL_ID=143526" "QTL_ID=143528" "QTL_ID=126011"

$ssGWAS_NCSNP_H0.80
[1] "QTL_ID=125442" "QTL_ID=177018" "QTL_ID=42094" "QTL_ID=42095" "QTL_ID=42098" "QTL_ID=42099"
[7] "QTL_ID=42105" "QTL_ID=42106" "QTL_ID=42110" "QTL_ID=42111" "QTL_ID=42112" "QTL_ID=42115"
[13] "QTL_ID=42116" "QTL_ID=42119" "QTL_ID=42120" "QTL_ID=42126" "QTL_ID=42127" "QTL_ID=42131"
[19] "QTL_ID=42132" "QTL_ID=42133" "QTL_ID=42096" "QTL_ID=42100" "QTL_ID=42103" "QTL_ID=42104"
[25] "QTL_ID=42117" "QTL_ID=42121" "QTL_ID=42124" "QTL_ID=42125" "QTL_ID=42092" "QTL_ID=42097"
[31] "QTL_ID=42101" "QTL_ID=42102" "QTL_ID=42107" "QTL_ID=42113" "QTL_ID=42118" "QTL_ID=42122"
[37] "QTL_ID=42123" "QTL_ID=42128" "QTL_ID=181936" "QTL_ID=182379" "QTL_ID=184562" "QTL_ID=185307"
[43] "QTL_ID=185502" "QTL_ID=185746" "QTL_ID=185966" "QTL_ID=187366" "QTL_ID=187474" "QTL_ID=188492"
[49] "QTL_ID=188805" "QTL_ID=189289" "QTL_ID=189510" "QTL_ID=189637" "QTL_ID=42093" "QTL_ID=42108"
[55] "QTL_ID=42109" "QTL_ID=42114" "QTL_ID=42129" "QTL_ID=42130" "QTL_ID=212624" "QTL_ID=178966"
[61] "QTL_ID=68117" "QTL_ID=68118" "QTL_ID=68310" "QTL_ID=135799" "QTL_ID=68559" "QTL_ID=176799"
[67] "QTL_ID=177143" "QTL_ID=165364" "QTL_ID=107642"

$`ssGWAS_NCSNP_H0.15:ssGWAS_NCSNP_H0.50`
[1] "QTL_ID=65985" "QTL_ID=113783" "QTL_ID=116938" "QTL_ID=62078"

$`ssGWAS_NCSNP_H0.15:ssGWAS_NCSNP_H0.80`
[1] "QTL_ID=158090" "QTL_ID=175015"

$`ssGWAS_NCSNP_H0.50:ssGWAS_NCSNP_H0.80`
[1] "QTL_ID=21147" "QTL_ID=29831" "QTL_ID=29833" "QTL_ID=32557" "QTL_ID=106980" "QTL_ID=107136"
[7] "QTL_ID=158047" "QTL_ID=15493" "QTL_ID=15494" "QTL_ID=35479" "QTL_ID=35948"

$`ssGWAS_NCSNP_H0.15:ssGWAS_NCSNP_H0.50:ssGWAS_NCSNP_H0.80`
[1] "QTL_ID=64006" "QTL_ID=64007" "QTL_ID=41728" "QTL_ID=41731" "QTL_ID=41732" "QTL_ID=41737"
[7] "QTL_ID=41738" "QTL_ID=41739" "QTL_ID=41741" "QTL_ID=41742" "QTL_ID=41743" "QTL_ID=41729"
[13] "QTL_ID=41733" "QTL_ID=41735" "QTL_ID=41736" "QTL_ID=41726" "QTL_ID=41730" "QTL_ID=41734"
[19] "QTL_ID=41740" "QTL_ID=41727" "QTL_ID=43369" "QTL_ID=43370" "QTL_ID=43372" "QTL_ID=43373"
[25] "QTL_ID=43374" "QTL_ID=43375" "QTL_ID=43376" "QTL_ID=43378" "QTL_ID=43379" "QTL_ID=43380"
[31] "QTL_ID=43381" "QTL_ID=43383" "QTL_ID=43384" "QTL_ID=43386" "QTL_ID=43387" "QTL_ID=43388"
[37] "QTL_ID=43389" "QTL_ID=43390" "QTL_ID=43392" "QTL_ID=43393" "QTL_ID=43394" "QTL_ID=43395"
[43] "QTL_ID=43368" "QTL_ID=43371" "QTL_ID=43377" "QTL_ID=43382" "QTL_ID=43385" "QTL_ID=43391"
[49] "QTL_ID=50404" "QTL_ID=50406" "QTL_ID=50407" "QTL_ID=50409" "QTL_ID=50413" "QTL_ID=50414"
[55] "QTL_ID=50415" "QTL_ID=50416" "QTL_ID=50417" "QTL_ID=50412" "QTL_ID=164127" "QTL_ID=164206"
[61] "QTL_ID=164416" "QTL_ID=164436" "QTL_ID=164476" "QTL_ID=164535" "QTL_ID=176528" "QTL_ID=186241"
[67] "QTL_ID=190174" "QTL_ID=50405" "QTL_ID=50408" "QTL_ID=50410" "QTL_ID=66693" "QTL_ID=50403"
[73] "QTL_ID=50411" "QTL_ID=157031" "QTL_ID=157030" "QTL_ID=14785"
