# Supplementary material for: Haplotype-Based Single-Step GWAS for Yearling Temperament in American Angus Cattle
Source: Genes (Basel). 2021 Dec 22;13(1):17. doi: 10.3390/genes13010017 (PMC8775055; doi:10.3390/genes13010017)
Supplement: Supplementary file 1 [file genes-13-00017-s001.zip › genes-1492001-supplementary/Supplementary_File_S9.pdf]

genes

MDPI

Supplementary Table S9. Correlations between the genomic estimated breeding values (GEBV) used in the single-step GWAS (ssGWAS) and weighted ssGWAS in the second (WssGWAS_2) and third (WssGWAS_3) iterations with haplotypes and non-clustered SNPs for yearling temperament in American Angus.

Marker Information	LD[1]	WssGWAS_2	WssGWAS_3
Haplotypes	0.15	0.988	0.982
	0.50	0.990	0.984
	0.80	0.987	0.981
NCSNP and Haplotypes	0.15	0.992	0.989
	0.50	0.994	0.991
	0.80	0.989	0.985

[1] Linkage disequilibrium.
